# Evaluating Models of Cellulose Degradation by *Fibrobacter succinogenes* S85

**DOI:** 10.1371/journal.pone.0143809

**Published:** 2015-12-02

**Authors:** Meagan C. Burnet, Alice C. Dohnalkova, Anthony P. Neumann, Mary S. Lipton, Richard D. Smith, Garret Suen, Stephen J. Callister

**Affiliations:** 1 Biological Sciences Division, Pacific Northwest National Laboratory, Richland, Washington, 99352, United States of America; 2 Environmental Molecular Sciences Laboratory, Pacific Northwest National Laboratory, Richland, Washington, 99352, United States of America; 3 Department of Bacteriology, University of Wisconsin-Madison, Madison, Wisconsin, 53706, United States of America; Centre National de la Recherche Scientifique, Aix-Marseille Université, FRANCE

## Abstract

*Fibrobacter succinogenes* S85 is an anaerobic non-cellulosome utilizing cellulolytic bacterium originally isolated from the cow rumen microbial community. Efforts to elucidate its cellulolytic machinery have resulted in the proposal of numerous models which involve cell-surface attachment via a combination of cellulose-binding fibro-slime proteins and pili, the production of cellulolytic vesicles, and the entry of cellulose fibers into the periplasmic space. Here, we used a combination of RNA-sequencing, proteomics, and transmission electron microscopy (TEM) to further clarify the cellulolytic mechanism of *F*. *succinogenes*. Our RNA-sequence analysis shows that genes encoding type II and III secretion systems, fibro-slime proteins, and pili are differentially expressed on cellulose, relative to glucose. A subcellular fractionation of cells grown on cellulose revealed that carbohydrate active enzymes associated with cellulose deconstruction and fibro-slime proteins were greater in the extracellular medium, as compared to the periplasm and outer membrane fractions. TEMs of samples harvested at mid-exponential and stationary phases of growth on cellulose and glucose showed the presence of grooves in the cellulose between the bacterial cells and substrate, suggesting enzymes work extracellularly for cellulose degradation. Membrane vesicles were only observed in stationary phase cultures grown on cellulose. These results provide evidence that *F*. *succinogenes* attaches to cellulose fibers using fibro-slime and pili, produces cellulases, such as endoglucanases, that are secreted extracellularly using type II and III secretion systems, and degrades the cellulose into cellodextrins that are then imported back into the periplasm for further digestion by β-glucanases and other cellulases.

## Introduction

The Gram-negative, obligate anaerobic bacterium, *Fibrobacter succinogenes*, is an important degrader of lignocellulosic plant material in the herbivore gut, making it of special interest for biofuel production [1, 2]. However, the mechanism employed by the type strain S85 (*F*. *succinogenes* ATCC 19169) for lignocellulose deconstruction and the proteins involved in this enzymatic function have not been clearly delineated. Unlike other model anaerobic cellulolytic microorganisms that degrade cellulose using cellulosomes, evidence suggests that *F*. *succinogenes* does not contain the signature proteins of a cellulosome, such as scaffoldins and dockerin-binding domains [3]. In addition, predicted cellulase genes did not contain carbohydrate binding modules that are affiliated with binding to crystalline cellulose. This suggests that *F*. *succinogenes* likely uses an alternate mechanism for degrading cellulose.

Many enzymatic assays of *F*. *succinogenes* grown on cellulose as the sole carbon source have been done previously. Based on these assays, a high proportion of the endoglucanases, xylanases, and cellulases produced by these cells were found to be released from the cells into the extracellular medium in addition to membrane vesicles, which were thought to be involved in cellulose degradation [4–7]. These membrane vesicles found in *F*. *succinogenes* cellulose cultures were later suggested not to have a role in cellulose degradation, but were observed as a sign of aging cells [8]. In addition, outer membrane, extracellular proteins and membrane vesicles from cellulose grown cells showed higher acetylesterase, endoglucanase and xylanase activities than the cytoplasm, inner membrane, and periplasm [7].

A key step in understanding this mechanism was elucidated by Gong and colleagues, who identified a 180 kDa cellulose binding protein with a role in adhesion to cellulose [9]. Since most anaerobic cellulose degrading bacteria rely upon strict binding of the cell to the cellulose fiber, this discovery led to the proposal of a class of binding proteins termed “fibro-slime” proteins that are specific to *F*. *succinogenes*, and thought to be localized to the outer membrane. These fibro-slime proteins were also shown to be involved in adhesion to and/or degradation of cellulose [10]. Further analysis of the *F*. *succinogenes* genome sequence led to a proposed mechanism for cellulose deconstruction that involves both fibro-slime and type IV pilin proteins as a means of attaching the outer membrane to the cellulose fiber. Under this model, the individual cellulose chains would be transported through the outer membrane via ABC transporters and degraded in the periplasmic space [3, 11].

To further investigate these proposed mechanisms, we employed a combination of transcriptomics, proteomics, and transmission electron microscopy (TEM) on *F*. *succinogenes* cells. RNA was sequenced from *F*. *succinogenes* grown on glucose and cellulose to observe changes in gene expression between cells grown on the two substrates. Subcellular fractionation of *F*. *succinogenes* cells grown on cellulose was used to extract proteins from the outer membrane, periplasm, and extracellular medium, and the total number and abundance of cellulose degrading proteins were compared. TEMs were also performed on samples grown to mid-exponential and stationary phase on glucose and cellulose to observe the adherence patterns of bacterial cells to cellulose and ascertain the presence of membrane vesicles. Taken together, our data provides evidence that cellulose degradation by *F*. *succinogenes* involves the use of both fibro-slime and pilin proteins for cell attachment and the production of extracellular enzymes to degrade cellulose into smaller polysaccharides, which are then imported back into the periplasm for further deconstruction.

## Materials and Methods

### Bacterial Cultures

*Fibrobacter succinogenes* S85 (ATCC 19169) cultures were grown at 37°C in a defined medium supplemented with either 4 g L^-1^ microcrystalline cellulose or 4 g L^-1^ glucose as the primary carbon source in either 18 x 150 mm anaerobic tubes or 200 ml serum vials with gas impermeable butyl rubber stoppers and 20 mm aluminum crimp seals (Bellco Glass, Vineland, NJ). Inoculations were performed using a sterile needle and syringe in order to maintain strict anaerobic conditions. A slightly modified version of a medium originally described by Scott and Dehority was used [12, 13]. Briefly, a basal medium containing NaCl, (NH_4_)_2_SO_4_, CaCl_2_, MgCl_2_, MnCl_2_, FeSO_4_, ZnCl_2_, and CoCl_2_ was prepared and dispensed into culture vessels under CO_2_. The culture vessels were sealed with butyl rubber stoppers and aluminum crimp seals and sterilized by autoclaving. Separate sterile solutions of Na_2_CO_3_, KH_2_PO_4_, volatile fatty acids (VFAs), vitamins, and the appropriate carbon source were added individually to the culture vessels via sterile needle and syringe immediately prior to use. Lastly, sterile L-Cysteine-HCl was added in the same manner to reduce the medium before inoculation. Resazurin sodium salt was included in the medium as an indicator of anaerobic conditions. The exact concentrations and components in the final medium are listed in S5 Table. *F*. *succinogenes* reached mid-exponential phase at 27 hours and stationary phase after 36 hours of growth on cellulose. The glucose cultures reached mid-exponential phase at 12 hours and stationary phase after 17 hours of growth.

### RNA Sequencing and Analysis

Ten mL from each of three replicate batch cultures for each carbon source, Sigmacell 50 (Sigma-Aldrich, St. Louis, MO) or glucose, was collected during mid-exponential phase growth for RNA-Seq [14] to determine differences in expression between the carbon sources. RNA was recovered using phenol-chloroform extraction followed by ethanol precipitation [15]. Residual DNA was degraded using TURBO DNA-free (Life Technologies, Carlsbad, CA) and rRNA reduced with a Ribo-Zero Magnetic kit (Epicentre, Madison, WI). RNA was evaluated prior to and following rRNA reduction using an Agilent 2100 Bioanalyzer (Agilent Technologies Inc., Santa Clara, CA) to assess integrity before proceeding with cDNA library preparation and sequencing. cDNA libraries were prepared using a TruSeq RNA sample preparation kit (Illumina, San Diego, CA) and sequenced using an Illumina HiSeq 2000 at the University of Wisconsin-Madison Biotechnology Center.

Read mapping, normalization, quantification, and testing for differential expression between the carbon sources was achieved using the freely available software package Rockhopper [16], which is specifically designed for analyzing RNA-sequence data from prokaryotic sources. Significant differences in gene expression were determined by calculating *q*-values based on the Benjamini and Hochberg correction with a false discovery rate < 1% [17]. Fastq files for the six samples have been deposited at the National Center for Biotechnology Information (NCBI) Short Read Archive (http://www.ncbi.nlm.nih.gov/sra) and can be found under project accession PRJNA287715.

A Clusters of Orthologous Groups (COG) analysis was performed on those open reading frames (ORFs) differentially expressed on cellulose, relative to glucose by comparing them against all predicted ORFs in the genome. Total counts for differentially expressed ORFs according to each COG category were determined, and over- or under-enrichment relative to all ORFs in the genome was calculated using a two-tailed Fisher’s Exact Test. COG categories statistically significant for over- or under-enrichment were considered at a *P*-value < 0.05.

### Subcellular Fractionation

A subcellular fractionation procedure was performed on *F*. *succinogenes* using a protocol we adapted from Miron and Forsberg [18]. Harvested *F*. *succinogenes* cellulose cultures were centrifuged at 650 x *g* for 5 minutes at 4°C to remove cellulose [7]. The removed cellulose was not processed and the cellulose depleted medium was centrifuged at 8,000 x *g* for 20 minutes at 4°C to separate the cells from the extracellular medium. The extracellular medium was separated from the cell pellet and centrifuged at 14,000 x *g* for 20 minutes at 4°C to pellet any remaining cells. The cell pellet was resuspended in 4.5 mL of 0.5 M NaCl, 20 mM PIPES pH 6.7, shaken at 150 rpm for 5 minutes at 4°C and then centrifuged at 23,000 x *g* for 20 minutes at 4°C. The wash was collected and the pellet was resuspended, shaken and centrifuged as stated above. The wash was collected with the previous washes and the pellet was resuspended in 2.25 mL of 24% w/v sucrose/1 mM EDTA in 20 mM PIPES pH 6.8. Samples were shaken at 150 rpm for 10 minutes at 4°C. An additional 2.25 mL of 20 mM PIPES pH 6.7 was added and the sample was centrifuged at 23,000 x *g* for 30 minutes at 4°C. The wash was collected with the previous washes. The 0.5 M NaCl, 20 mM PIPES pH 6.7 and 24% w/v sucrose/1 mM EDTA in 20 mM PIPES pH 6.8 washes were centrifuged at 100,000 x *g* for 1 hour at 4°C. The pellet, which consisted of the outer membrane proteins, was resuspended in 600 uL of 100 mM ammonium bicarbonate pH 8. The supernatant contained the periplasmic proteins.

The extracellular medium and periplasmic protein samples were concentrated using 3K Amicon Ultra Centrifugal Filter devices (Merck Millipore Ltd., Tullagreen, Carrigtwohill, C. Cork, IRL.) and exchanged into 100 mM ammonia bicarbonate pH 8. Protein concentration was determined for the outer membrane, periplasmic and extracellular medium protein samples by a BCA protein assay (Thermo Scientific, Rockford, IL) then samples were denatured and reduced by adding a final concentration of 8 M urea and fresh dithiothreitol (DTT) to a final concentration of 5 mM. The samples were incubated at 60°C for 30 minutes with 850 rpm shaking then diluted 10 fold with 100 mM ammonium bicarbonate pH 8. CaCl_2_ was added to a final concentration of 1 mM and samples were digested for 3 hours at 37°C using USB Trypsin at a concentration of 1 ug trypsin/50 ug protein. After trypsin incubation, digested samples were desalted using 1 mL Discovery C18 SPE columns (Supelco, Bellefonte, PA) and dried to 50 uL using a speedvac concentrator (Savant, Holbrook, NY). Samples were centrifuged at 10,000 x *g* for 5 minutes and peptide concentration was determined by a BCA protein assay. Samples were diluted to 0.1 ug/uL and placed in vials for MS analysis.

### Liquid Chromatography-Mass Spectrometry based Proteome Analysis

The following liquid chromatography tandem mass spectrometry analysis (LC-MS/MS) was adapted from the procedure described in Robidart et al., 2013 [19]. High resolution, reversed-phase, constant-pressure capillary liquid chromatography peptide separations used in-house manufactured columns (60 cm x 360 μm o.d. 75 μm i.d. fused silica capillary tubing) packed with 3 μm Jupiter C18 stationary phase (Phenomenex, Torrence, CA). Columns were equilibrated for a minimum of 100 minutes, preceding sample injection, with 100% mobile phase A (0.1% formic acid in water). Reverse-phase separation ensued by means of fifty minutes after injection, mobile phase B (0.1% formic acid in acetonitrile) was introduced to transpose mobile phase A, which created an exponential gradient. A 40-cm length of 360 μm o.d. x 15 μm i.d. fused silica tubing was used to split approximately 20 μL/min flow before it entered the injection value (5 μL sample loop). The split flow restrained the gradient speed under a pressure operation of 10 K psi. Flow through the capillary HPLC column when equilibrated to 100% mobile phase A was approximately 400 nL min^-1^. Mass spectrometry analysis was performed with a Thermo Electron ion trap LTQ Orbitrap MS (Thermo Scientific, San Jose, CA) equipped with an ion funnel and electrospray ionization (ESI) interface. Orbitrap spectra were collected from 400 to 2,000 m/z at a resolution of 100k followed by data-dependent ion trap MS/MS spectra of the six most abundant ions using 35% collision energy. A 30 second dynamic exclusion was used to discriminate against previously analyzed ions.

MS/MS raw data was analyzed using the MSGF+ database search algorithm [20] and the *F*. *succinogenes* S85 annotated genome (*F*. *succinogenes* S85 ATCC 19169 Genbank Accession CP001792.1; downloaded_2012_04_07). Only peptides with a spectral probability less than 1 x 10^−10^ and 6 amino acids or longer in length with a false discovery rate (FDR) of <1% were retained. Peptide redundancy was removed so that each peptide sequence was unique to a single protein. Only proteins identified by ≥ 2 unique peptides were evaluated further. Spectral counting was used to estimate protein abundance. Enzymes were identified as carbohydrate active enzymes (CAZymes) by searching the CAZy [21] protein databases.

### Transmission Electron Microscopy (TEM)

Harvested *F*. *succinogenes* mid-exponential and stationary phase cultures grown on glucose and cellulose were gently pelleted by centrifugation (2,500 x *g*, 1 min), fixed in 2.5% glutaraldehyde (Electron Microscopy Sciences (EMS), Harfield, PA) and processed by washing the cells 3x with dH_2_O before incubation in 1% osmium tetraoxide for 2 hours at room temperature. Cells were washed with dH_2_O and dehydrated by using a gradual ethanol series (25, 33, 50, 75, and 3x 100% ethanol). Samples were then gradually infiltrated in LR White acrylic resin (EMS). After polymerization at 60°C for 24 hours, the hardened resin blocks were sectioned on a Leica EM UC6 ultramicrotome using a 45° diamond knife (Diatome). Seventy-nanometer ultrathin sections were post-stained with 2% uranyl acetate and Reinold’s lead citrate (7 and 3 min, respectively), and imaged in a Tecnai T-12 TEM (FEI) with a LaB6 filament, operating at 120 kV. Images were collected digitally with a 2x2K Ultrascan 1000 CCD (Gatan).

## Results

### Genes Exhibiting Differential Expression

A total of 1,907 genes in *F*. *succinogenes* were found to be differentially expressed (q-value <0.01) in either glucose or cellulose cultures. This observation represents 62% of predicted protein coding genes in the genome. Out of the 1,907 transcripts, 1,886 genes were observed in common to both cultures, a 99% overlap, while 7 gene transcripts were uniquely observed in the cellulose cultures and 9 in the glucose cultures (S1 Table). To determine the broad scale transcriptional changes that occur as a result of growth on cellulose, we subjected our RNA sequence data to a COG enrichment analysis as shown in S2 Table. A number of COG categories were found to be enriched in our RNA sequence data, relative to all genes in the genome including Transcription, Signal Transduction Mechanisms, Cell Motility, and Carbohydrate Transport and Metabolism.

To gain a better understanding of the cellulolytic response of *F*. *succinogenes*, we examined the transcripts of those genes annotated as carbohydrate active enzymes (CAZymes). Genes for 134 CAZymes were expressed by *F*. *succinogenes* in the presence of microcrystalline cellulose, representing 71% of the CAZymes predicted for this organism [21]. Many of the observed CAZymes are annotated as hemicellulases (S1 Table), suggesting that these genes are under similar regulatory control to those genes involved in cellulose deconstruction. Interestingly, 133 of 134 CAZymes were also observed in the glucose cultures. We also found that expression of 1 CAZymes gene was uniquely observed in the cellulose cultures, which was a mannosidase. There were no CAZymes genes found to be expressed exclusively in the glucose cultures.

**Fig 1:**
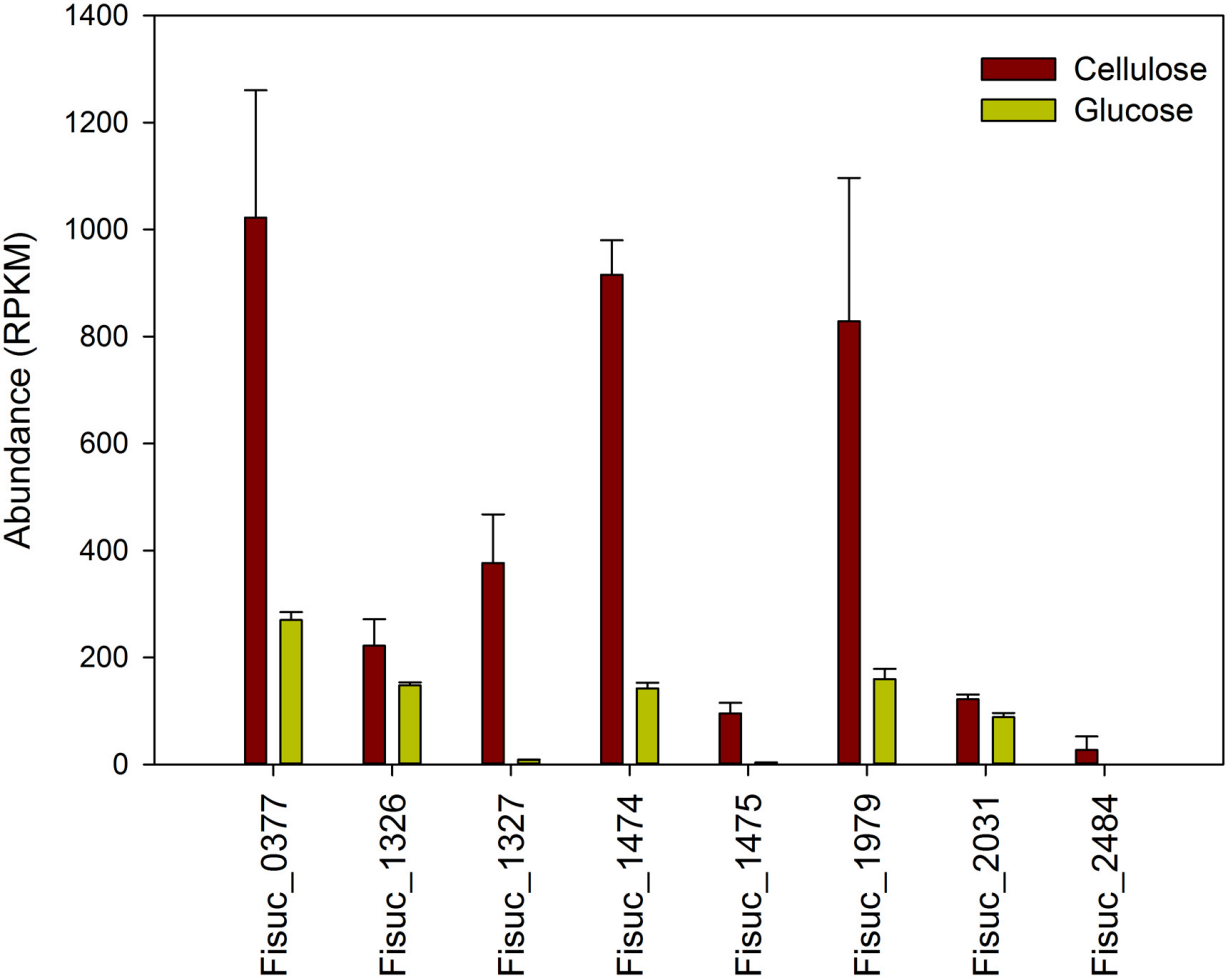
Expression of fibro-slime genes in *F*. *succinogenes* S85 cellulose and glucose cultures. The cellulose cultures showed an increased expression for 8 out of 10 fibro-slime proteins relative to the glucose cultures. RPKM = Reads Per Kilobase per Million mapped reads.

**Fig 2:**
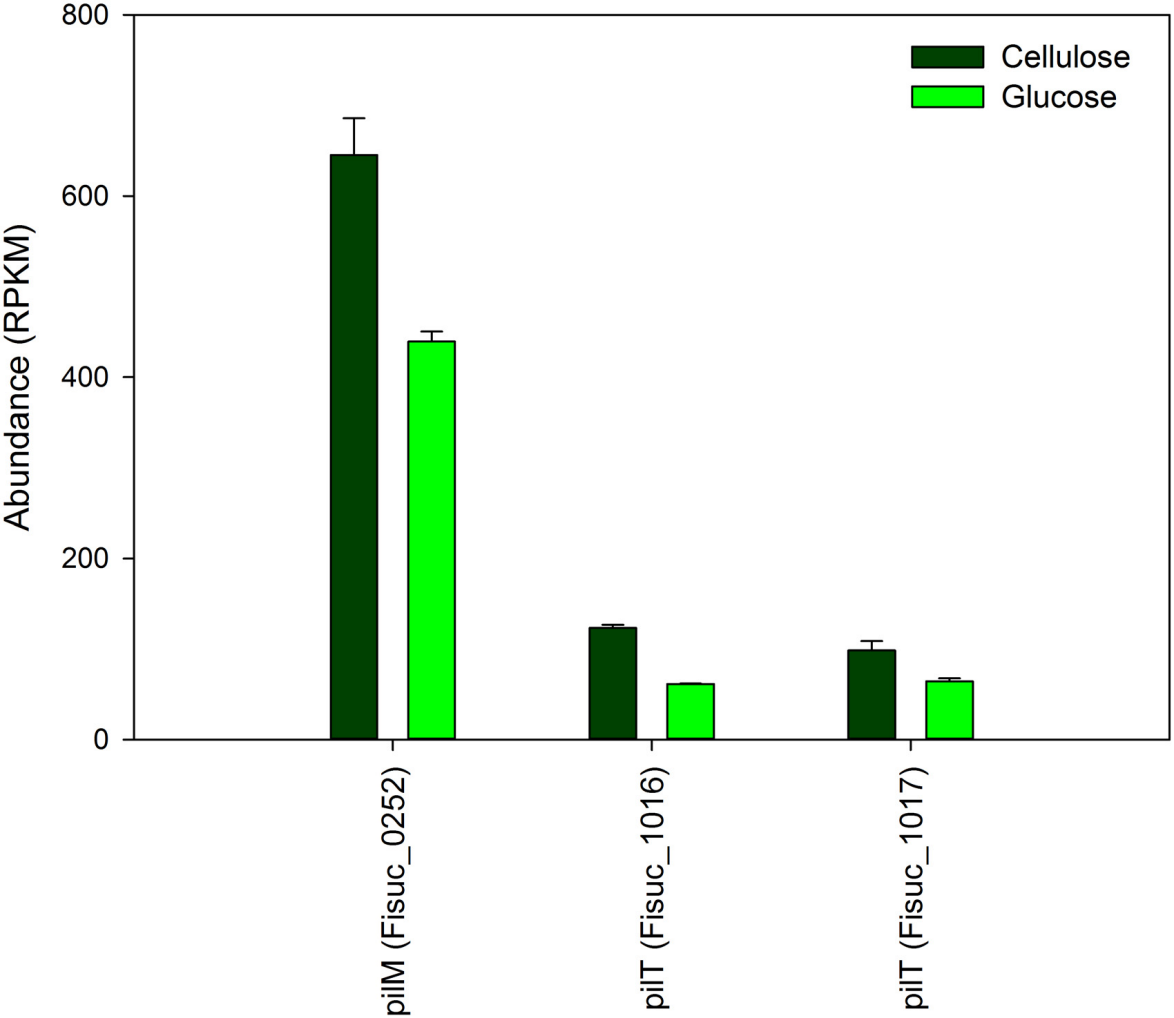
Expression of pilin transcripts in *F*. *succinogenes* S85 cellulose and glucose cultures. There is a higher expression of pilin proteins in the cellulose cultures than the glucose cultures for 3 out of the 4 pilin proteins observed. RPKM = Reads Per Kilobase per Million mapped reads.

**Fig 3:**
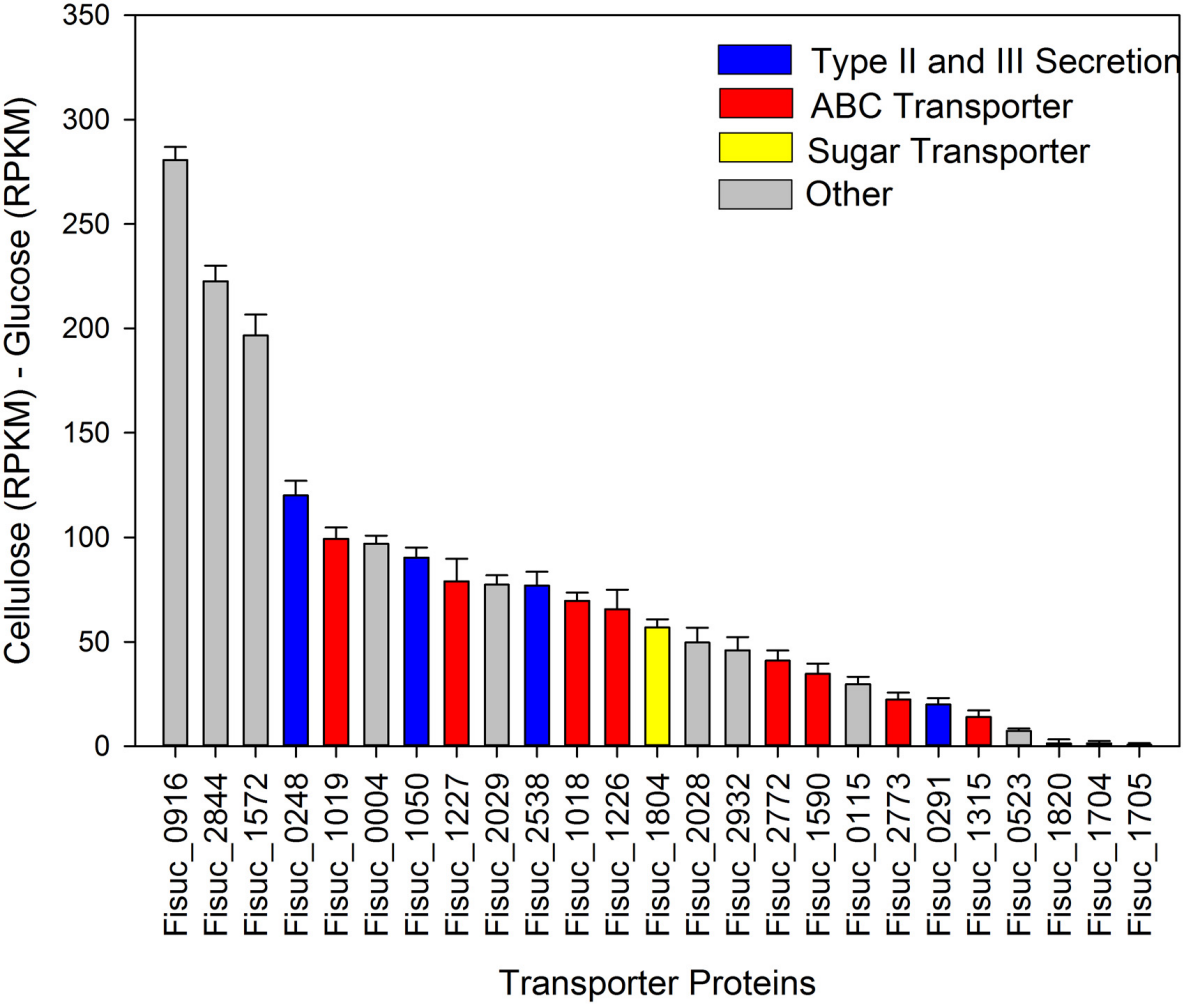
Difference in expression between cellulose and glucose cultures for transporters, secretion systems, effluxes, and exporters genes. All type II and III secretion systems had higher expression in the cellulose cultures than the glucose cultures suggesting they may have a role in secreting the carbohydrate active enzymes into the extracellular medium. RPKM = Reads Per Kilobase per Million mapped reads.

We also evaluated the expression of non-CAZymes protein coding genes predicted to be involved in cellulose deconstruction, such as fibro-slime proteins, pilin and transporters [3, 11]. For the fibro-slime genes, 8 out of 10 showed increased expression in the cellulose cultures relative to the glucose cultures (Fig 1). Fisuc_2293 and Fisuc_2471 exhibited relatively higher expression in the glucose cultures, but this was not considered significant as their q-values were greater than 0.01. Three out of the four annotated pilin genes also showed an increase in relative expression in the cellulose cultures; although, the difference was less than 2-fold, on average (Fig 2). Transcripts were observed for 55 transporter associated genes including ABC transporters, secretion system genes, efflux pumps, and exporters in both cellulose and glucose cultures (Fig 3). The expression of ABC transporter genes varied between the cellulose and glucose cultures. Ten transcripts of ABC transporter associated genes had a higher expression in cellulose than glucose. However, all the type II and III secretion systems had higher expression in the cellulose compared to the glucose cultures. Fisuc_1804, an annotated sugar transporter, was found to have higher expression in the cellulose cultures, relative to glucose (Fig 3).

### Observed Proteins within Subcellular Fractions

**Fig 4:**
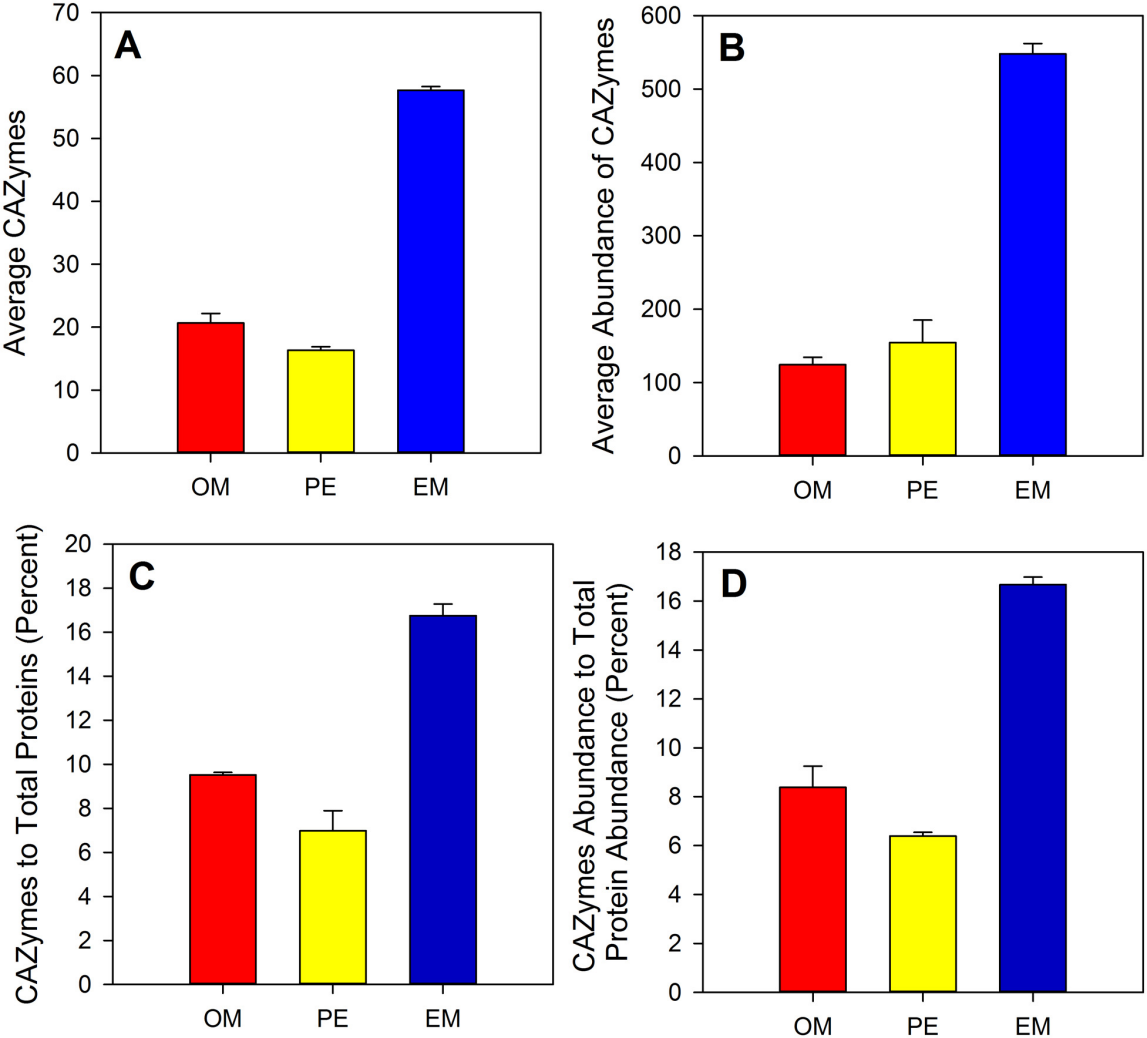
(A) Average number of carbohydrate active enzymes (CAZymes) and (B) average abundance of CAZymes observed from a given fraction. (C) Percent of CAZymes to total proteins and (D) percent CAZymes abundance to total protein abundance. The number and percentages of CAZymes and abundance of CAZymes was largest for the extracellular medium fraction. EM = extracellular medium, OM = outer membrane, PE = periplasm.

To further understand the cellulolytic machinery of *F*. *succinogenes*, we conducted a proteomics analysis of proteins obtained from a subcellular fractionation of cells grown to mid-exponential phase on cellulose. A total of 590 proteins identified by 2 or more unique peptides were observed from the extracellular growth medium, outer membrane and periplasm fractions originating from microcrystalline cellulose cultures. The selection of these fractions for analysis is consistent with other studies focused on cellulose deconstruction by *F*. *succinogenes* [7, 10, 11]. A comparison between fractions revealed a greater number of proteins associated with the extracellular medium (355 ± 10) than the periplasm (236 ± 30) or outer membrane (217 ± 18) fractions. In regard to CAZymes the largest number, on average (58 ± 1), was also extracted from the extracellular medium (Fig 4A). A similar observation was found when evaluating the abundance of CAZymes within each fraction (Fig 4B), and the proportion of CAZymes to total proteins and total protein abundance (Fig 4C and 4D).

**Fig 5:**
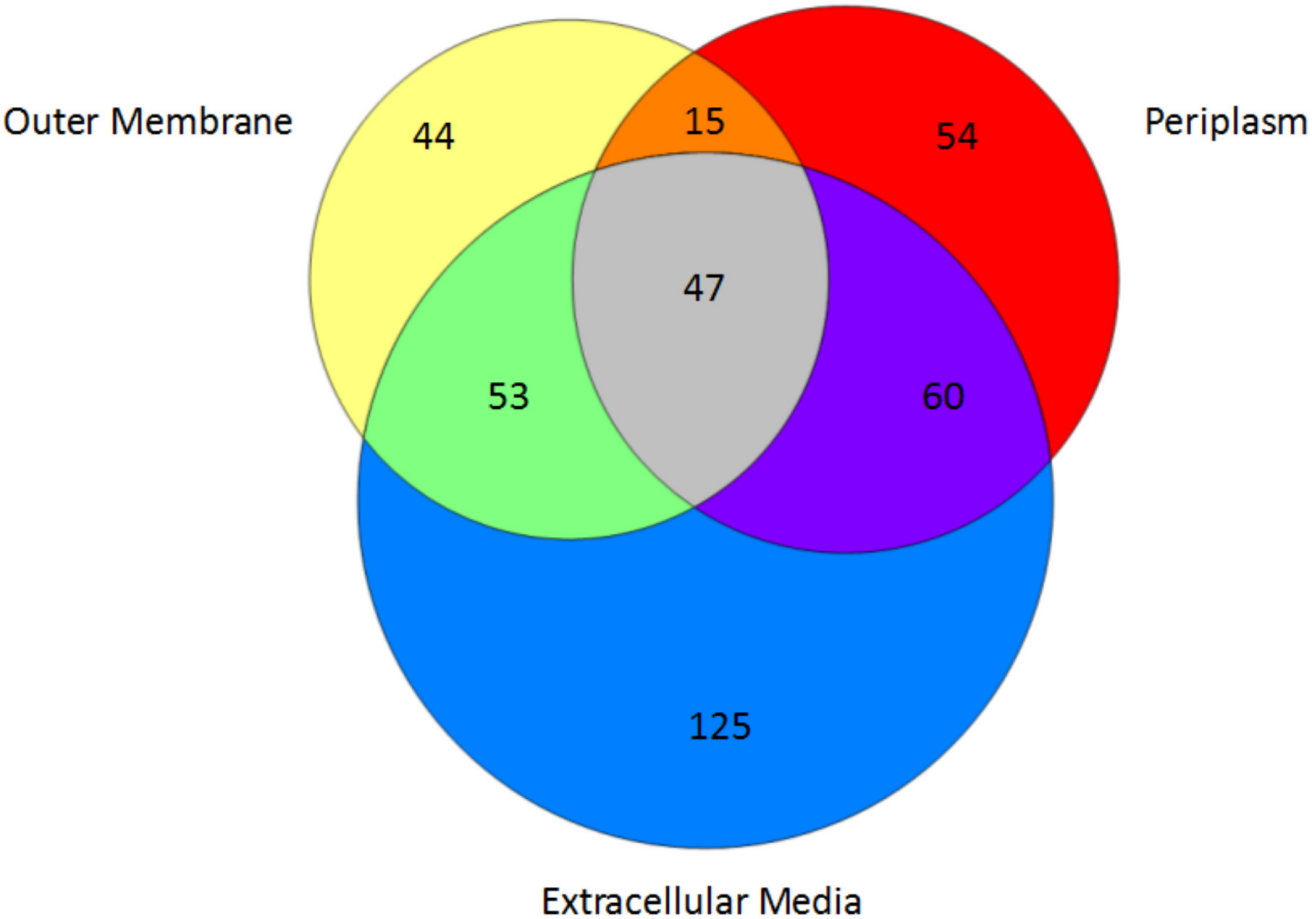
Comparison of the number of identified proteins and their overlap observed in the outer membrane, periplasm and extracellular medium fractions.

While many proteins including the CAZymes above were observed as unique to a given fraction, we also observed several proteins associated with more than one fraction, possibly from the challenge of separating pure fractions [22, 23]. To add confidence to our analysis we narrowed our list of proteins to only those observed in all biological replicates, reducing the total number of proteins from 590 to 398 (Fig 5). A portion of these proteins (56%) were observed solely within a single fraction, while 43% of the proteins were observed in two or more fractions, such as Fisuc_3111 (CBM11) observed in both the outer membrane and periplasm fractions and Fisuc_0377 (fibro-slime protein) observed in both the extracellular medium and outer membrane fraction. Examples of proteins observed in all three fractions include the GH5 (Fisuc_2364) and a GH8 (Fisuc_1802). In the instances where proteins were observed in multiple fractions (175 proteins), their significance within a given fraction was determined by the Kruskal-Wallis statistical hypothesis test (p-value < 0.05) [24] using measured relative protein abundances (S3 Table).

We found 60 CAZymes out of 398 total proteins remained after the statistical filtering (S3 Table). Thirteen CAZymes were not retained after filtering on three biological replicates. The extracellular medium contained the largest number of CAZymes with a total of 52. Thirty-six of these were uniquely observed in the extracellular medium, while an additional twelve were statistically assigned (four CAZymes had p-values > 0.05). The unique CAZymes observed included highly modular proteins containing multiple CBMs with an associated GH catalytic domain, e.g. Fisuc_1763—GH43, CBM6, CBM6, CBMnc; Fisuc_1793 –GH10, CBM6, CBMnc. The periplasm and outer membrane fractions contained the least amount of CAZymes with a total of 13 and 17, respectively. Our statistical analysis assigned 2 of the 13 CAZymes to the periplasm fraction including a cellodextrin-phosphorylase (Fisuc_2900), and a carbohydrate binding family 11 (Fisuc_3111), while an additional 5 of the 13 were observed as unique to this fraction (Table 1), including Fisuc_1641, annotated as a hypothetical protein but also classified as containing a carbohydrate esterase catalytic domain (CE2) [3]. Each of the 17 outer membrane observed CAZymes were also observed in more than one fraction, and we could not assign any of these to the outer membrane using our statistical analysis. After applying our stringent filter, the percentage of CAZymes to total proteins increased in the extracellular medium to 28% and the percentage of CAZymes abundance to total protein abundance increased to 21%.

**Table 1:** Carbohydrate active enzymes (CAZymes) and fibro-slime proteins observed from proteomes extracted from extracellular medium, outer membrane, and periplasm fractions.

Observed Fraction	Fisuc ID	Protein Description	Signal Peptide	Pvalue	CAZy Family
EM	Fisuc_1802	glycoside hydrolase family 8	NO	0.023	GH8
EM	Fisuc_2364	Cellulase	YES	0.027	GH5
EM	Fisuc_2031	fibro-slime family protein	YES	0.031	
EM	Fisuc_0786	Cellulase	YES	0.034	GH5
EM	Fisuc_1232	glycosyl transferase, family 2	NO	0.034	
EM	Fisuc_1530	glycoside hydrolase family 18	YES	0.043	GH18
EM	Fisuc_2362	glycoside hydrolase family 9	YES	0.043	GH9
EM	Fisuc_0377	fibro-slime family protein	YES	0.046	
EM	Fisuc_2579	glycoside hydrolase family 8	YES	0.046	GH8
EM	Fisuc_0394	glycoside hydrolase family 9	NO	0.046	GH9
EM	Fisuc_2250	O-Glycosyl hydrolase-like protein	YES	0.046	GH30
EM	Fisuc_1224	Cellulase	YES	0.050	GH5
EM	Fisuc_1474	fibro-slime family protein	YES	0.050	
EM	Fisuc_1979	fibro-slime family protein	YES	0.050	
EM	Fisuc_0393	glycoside hydrolase family 9	NO	0.050	GH9
EM	Fisuc_1859	glycoside hydrolase family 9	YES	0.050	GH9
EM	Fisuc_3103	1,4-alpha-glucan branching enzyme	NO	UNIQUE	CBM48,GH13
EM	Fisuc_0860	4-alpha-glucanotransferase	NO	UNIQUE	GH77
EM	Fisuc_1773	Alpha-galactosidase	YES	UNIQUE	GH27,CBM6
EM	Fisuc_1763	Carbohydrate binding family 6	YES	UNIQUE	GH43,CBM6,CBM6,CBMnc
EM	Fisuc_1764	Carbohydrate binding family 6	YES	UNIQUE	GH43,CBM6,CBM6
EM	Fisuc_1767	Carbohydrate binding family 6	YES	UNIQUE	CE6,CBM6,CBMnc
EM	Fisuc_1790	Carbohydrate binding family 6	YES	UNIQUE	GHnc,CBM6
EM	Fisuc_1793	Carbohydrate binding family 6	YES	UNIQUE	GH10,CBM6,CBMnc
EM	Fisuc_2478	Carbohydrate binding family 6	YES	UNIQUE	CE12,CBM35
EM	Fisuc_2477	Carbohydrate binding family 6	YES	UNIQUE	CBM35, PL11
EM	Fisuc_1931	Carbohydrate-binding CenC domain protein	NO	UNIQUE	CBM4
EM	Fisuc_1426	Cellulase	NO	UNIQUE	GH45
EM	Fisuc_2011	Cellulase	YES	UNIQUE	GH5
EM	Fisuc_0897	Cellulase	YES	UNIQUE	GH5
EM	Fisuc_2317	endo-1,4-beta-glucanase/xyloglucanase, putative, gly74A	YES	UNIQUE	GH74
EM	Fisuc_0362	Endo-1,4-beta-xylanase	YES	UNIQUE	GH11
EM	Fisuc_2442	Endo-1,4-beta-xylanase	YES	UNIQUE	GH11
EM	Fisuc_1326	fibro-slime family protein	YES	UNIQUE	
EM	Fisuc_1327	fibro-slime family protein	YES	UNIQUE	
EM	Fisuc_2293	fibro-slime family protein	NO	UNIQUE	
EM	Fisuc_1765	Glucuronoarabinoxylan endo-1,4-beta-xylanase	YES	UNIQUE	GH30,CBM6,CBMnc
EM	Fisuc_2424	glycoside hydrolase family 16	YES	UNIQUE	GH16
EM	Fisuc_1788	glycoside hydrolase family 2 TIM barrel	YES	UNIQUE	CBMnc, GH2
EM	Fisuc_0323	glycoside hydrolase family 44 domain protein	YES	UNIQUE	GH44
EM	Fisuc_0471	glycoside hydrolase family 8	YES	UNIQUE	GH8
EM	Fisuc_1219	glycoside hydrolase family 8	YES	UNIQUE	GH8
EM	Fisuc_1860	glycoside hydrolase family 9	YES	UNIQUE	GH9
EM	Fisuc_2033	glycoside hydrolase family 9	YES	UNIQUE	GH9
EM	Fisuc_1525	hypothetical protein	YES	UNIQUE	CBM30
EM	Fisuc_2012	hypothetical protein	YES	UNIQUE	PL1
EM	Fisuc_2479	lipolytic protein G-D-S-L family	NO	UNIQUE	CE12,CBM35
EM	Fisuc_0727	Mannan endo-1,4-beta-mannosidase	YES	UNIQUE	CBM35,GH26
EM	Fisuc_0728	Mannan endo-1,4-beta-mannosidase	YES	UNIQUE	GH5
EM	Fisuc_0729	Mannan endo-1,4-beta-mannosidase	YES	UNIQUE	CBM35,GH26
EM	Fisuc_0730	Mannan endo-1,4-beta-mannosidase	YES	UNIQUE	CBMnc,GH26
EM	Fisuc_2919	O-Glycosyl hydrolase-like protein	YES	UNIQUE	GH30
EM	Fisuc_2363	Pectate lyase/Amb allergen	YES	UNIQUE	PL1
EM	Fisuc_1991	Pectate lyase-like protein	YES	UNIQUE	CBM67,PL1
EM	Fisuc_0679	Pectinesterase	NO	UNIQUE	CE8,CBM35
PE	Fisuc_3111	Carbohydrate binding family 11	YES	0.046	CBM30,CBM30,CBM11,GH51
PE	Fisuc_2900	cellodextrin-phosphorylase	NO	0.046	GH94
PE	Fisuc_3049	Beta-galactosidase	NO	UNIQUE	GH2
PE	Fisuc_1531	Cellulase	YES	UNIQUE	GH9
PE	Fisuc_2065	glycoside hydrolase family 3 domain protein	YES	UNIQUE	GH3
PE	Fisuc_1641	hypothetical protein	YES	UNIQUE	CE2
PE	Fisuc_1000	Lytic transglycosylase catalytic	YES	UNIQUE	GH23

EM = extracellular medium, PE = periplasm, UNIQUE = observed in only one fraction and in all of the three replicates for that fraction.

**Fig 6:**
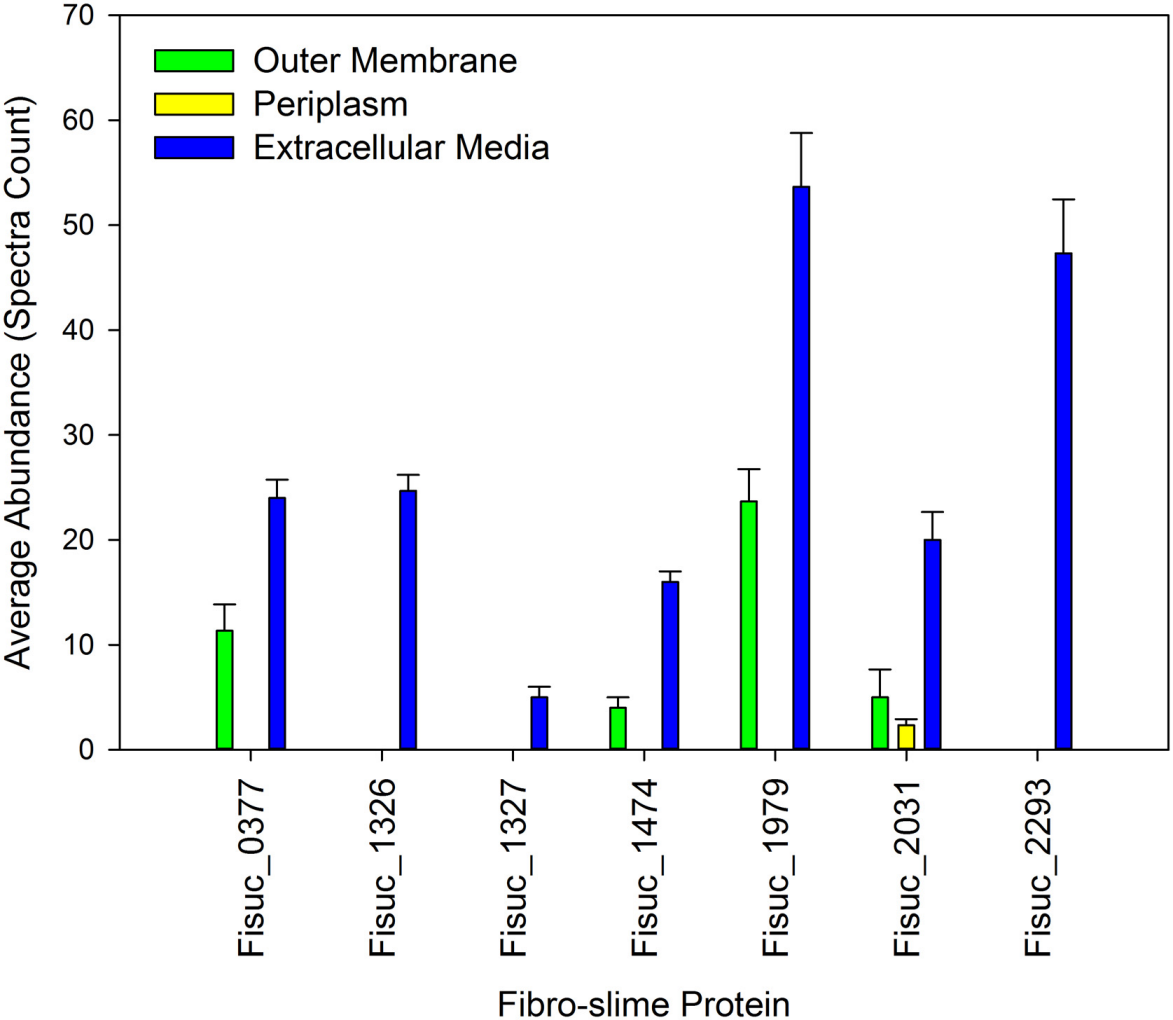
Average abundance of fibro-slime proteins identified from each fractionation. The average abundance for all fibro-slime proteins was the highest in the extracellular medium.

Seven of the ten predicted fibro-slime proteins (Table 1) were measured within the fractions (Fisuc_0377, 1326, 1327, 1474, 1979, 2031, 2293), with three of these (Fisuc_1326, 1327, 2293) uniquely observed in the extracellular medium. Three (Fisuc_0377, 1474, 1979) of the remaining four fibro-slime proteins were observed in the outer membrane and extracellular medium and one (Fisuc_2031) observed in all three fractions. The three fibro-slime proteins observed in both outer membrane and extracellular medium fractions had relatively higher abundances in the extracellular medium. Fisuc_2031, observed in all three fractions, also had a higher relative abundance in the extracellular medium than in the outer membrane or periplasm (Fig 6). Although all observed fibro-slime proteins were unique or statistically assigned to the extracellular medium, the observation of some of these within multiple fractions may indicate their association with more than one fraction (see Discussion).

A total of 122 hypothetical proteins were observed in the fractions. The extracellular medium fraction contained the largest number with 92, of which 51 (55%) were unique to this fraction. A total of 27 hypothetical proteins were associated with the periplasm, with 11 (41%) being unique. The least number of hypothetical proteins was assigned to the outer membrane fraction, with 17(30%) of 56 being unique. Gene expression for these hypothetical proteins was examined, and 23 of the 122 exhibited both a 2-fold increase in relative transcript abundance in the cellulose cultures and also passed our statistical significance filter (q-value < 0.01) (S1 Table). We found 8 of these hypothetical proteins assigned to the outer membrane, 4to the periplasm, and 22 to the extracellular medium. Interestingly, 13 of the 22 were solely observed within the extracellular medium.

### Transmission Electron Microscopy

**Fig 7:**
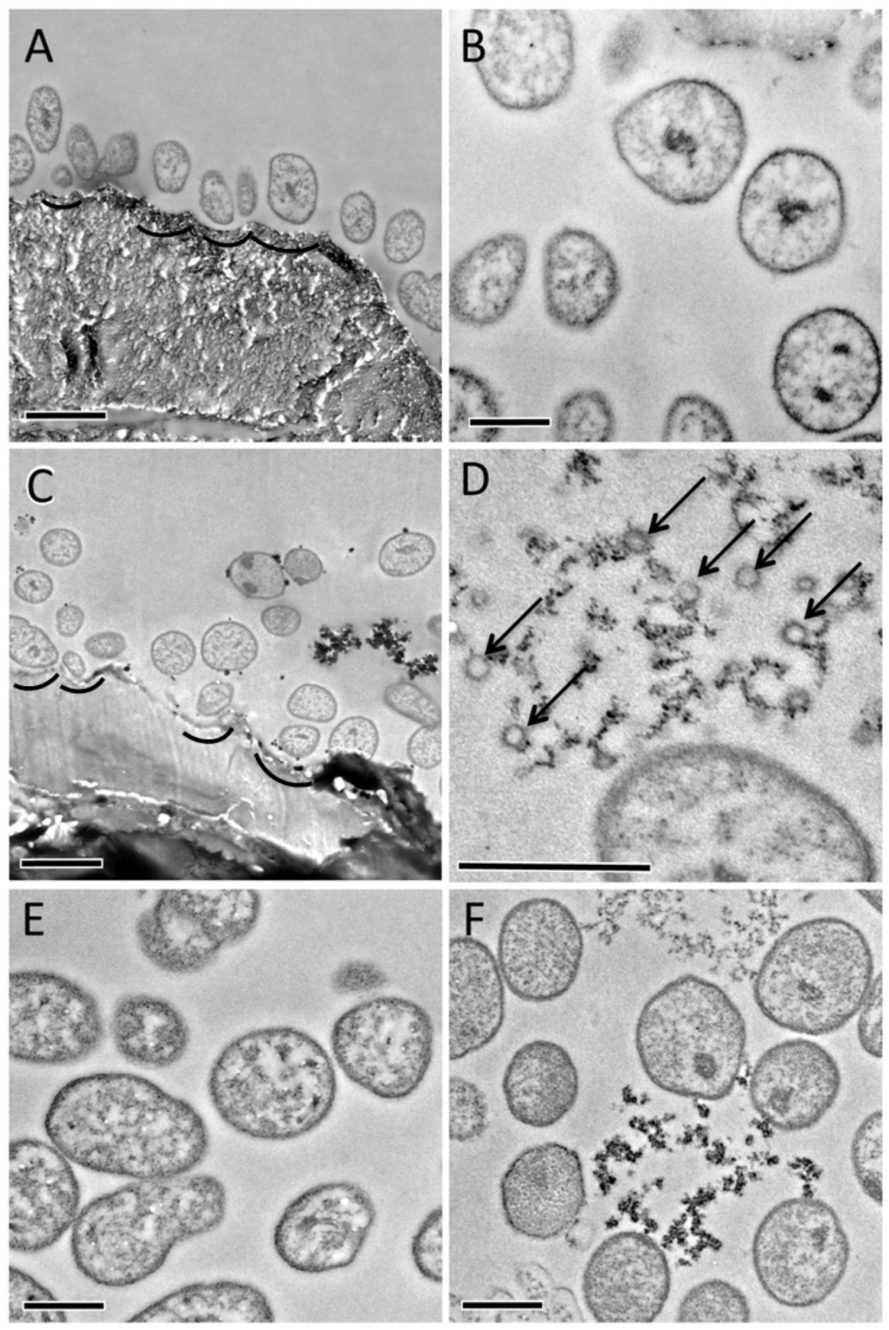
TEM images of *F*. *succinogenes* S85 harvested during mid-exponential growth on cellulose (A, B), cellulose stationary growth (C, D), glucose mid-exponential growth (E) and glucose stationary growth (F). Grooves in the cellulose were observed in the cellulose mid-exponential and stationary phases. Vesicles were present in only the cellulose stationary phase (arrows in D). Scale bars are 1 μm (A,C) and 0.5 μm (B-F). Vesicles are indicated by arrows. Grooves are indicated by curves.

A proposed model for *F*. *succinogenes* cellulose degradation involves the direct attachment of cells to cellulose fibers coupled to the production of extracellular vesicles. TEM images of *F*. *succinogenes* harvested at mid-exponential and stationary phases, grown under both glucose and cellulose, were examined for the presence of vesicles and cell orientation (Fig 7). No vesicles were observed from TEM images of cells from glucose mid-exponential and stationary phase and cellulose mid-exponential growth cultures. However, evidence of vesicles was observed in the cellulose stationary culture (Fig 7C and 7D). These potential vesicles were primarily observed unattached within the external medium, and no evidence of association with the microcrystalline cellulose was evident. Grooves were also observed within the cellulose in proximity to the cell surface, but no evidence of direct contact was found. However, the formation of such grooves does suggest a mechanism of association with the cellulose. We also observed thickening of the cell membrane for some cells on the side of the membrane facing towards the cellulose. Both cellulose mid-exponential and stationary cultures showed grooves in the cellulose with a thickening of the cell membrane in some instances. There was no apparent direct interaction of the cell outer membrane to the cellulose matrix but they appeared to be closely associated.

## Discussion

We investigated the mechanism used by *F*. *succinogenes* to degrade cellulose. Using a combination of transcriptomics, proteomics, and TEM analysis, we provide a global view into the underlying machinery that drives the prolific cellulolytic ability of this important rumen bacterium. Our results suggest that the majority of *F*. *succinogenes*’ cellulolytic activity occurs extracellularly, and that microcrystalline cellulose does not directly interact with the outer membrane of *F*. *succinogenes*. These findings are in contrast to a recent model that proposes a mechanism where polysaccharide fibers spanning the outer membrane are hydrolyzed within the periplasm [3, 11]. As a result, we propose a modification to this model whereby *F*. *succinogenes* cells attach to cellulose fibers using a combination of fibro-slime and pilin proteins coupled to the extracellular secretion of endoglucanases using type II or type III secretion systems.

This model is supported by our analysis of the global transcriptome of *F*. *succinogenes* cells, which revealed higher differential expression of genes encoding for fibro-slime, pilin, endoglucanases, and type II and III secretion proteins in cellulose cultures, relative to glucose. The pilin, type II and type III secretion system genes were also assigned to COG categories (categories “N” and” U” for pilin genes; “U” for type II and “N” and “U” for type III secretion system genes) that were over-enriched on cellulose relative to glucose (S2 Table). The genes encoding fibro-slime proteins were not assigned a COG category. Moreover, we found higher levels of expression for xylanases in cellulose cultures, relative to glucose, even though xylan was not present in the medium. This is consistent with the hypothesis that *F*. *succinogenes* may upregulate the transcription of these genes as part of a global response to the presence of plant polysaccharides, as xylan is an integral component of this substrate that would be encountered in its natural environment. We acknowledge that for our experimental set up, it was difficult to control for differences in gene expression resulting from differences in growth rates during mid-exponential growth on the two substrates. Although a systematic difference in gene expression was observed when evaluating ribosomal proteins (S1 Table), this difference was not found globally.

Our transcriptional data was further resolved through our proteomics analysis of the subcellular fractionation components of *F*. *succinogenes*. Importantly, we found that the majority of the CAZymes identified across all fractions were unique to the extracellular medium, and many of these are known endoglucanases. Given that all but one of these endoglucanases have predicted signal peptides and previous reports of greater enzymatic activity in the extracellular medium, relative to the cell pellet [5, 7], we conclude that *F*. *succinogenes* excretes many of its cellulolytic enzymes extracellularly. In addition to these endoglucanases, we found numerous other proteins in the extracellular medium fraction including 13 hypothetical proteins (S3 Table) that also exhibited > 2-fold expression in our transcriptomics analysis. We speculate that these proteins may play a role in cellulose deconstruction and warrant further investigation.

Previous work by Jun and colleagues (10) found numerous proteins in the outer membrane of *F*. *succinogenes* that may play a role in cellulose adhesion and deconstruction. We found none of these proteins to be statistically significant in the outer membrane fraction in our study, although some were found in the extracellular medium. For example, a GH9 (Fisuc_0393) and GH18 (Fisuc_1530) were found in both our outer membrane and extracellular medium fractions. However, the relative abundance was almost 6-fold higher in the extracellular medium than the outer membrane for both enzymes. Both of these enzymes have been previously identified as carbohydrate binding proteins in the outer membrane (10). This suggests that proteins involved in cellulose adhesion and deconstruction are secreted from the cell into the extracellular medium, or are loosely associated with the outer membrane [3]. A possible explanation for observing a CAZyme in multiple fractions would be due to the difficulty of obtaining pure fractions [22].

Our results also support a model whereby pilin and fibro-slime proteins facilitate cell-surface attachment to the cellulose and are involved in the binding of glycoside hydrolases to the substrate [3]. Previous work has shown that *F*. *succinogenes’* cell surface hydrophobicity was found to decrease when exposed to cellulose [25], similar to what has been observed for *Ruminococcus albus* [26, 27]. This finding in *R*. *albus* is thought to be associated with cell surface adhesion and involve the use of pili and we speculate that a similar mechanism may be employed by *F*. *succinogenes* given that both bacteria are found in the same rumen environment. In addition to pili, fibro-slime proteins have also been demonstrated to have a role in cellulose adhesion by Gong and colleagues [9]. In that study, *F*. *succinogenes* was treated with antibodies against the fibro-slime proteins, which blocked adhesion of the cell to cellulose by 62%. Importantly, an aggregation of immunoreactive fibro-slime proteins at the site of cellulose attachment was observed, implying that they have a role in adhesion to cellulose; however, the mechanism of their particular roles remains unknown [9]. More recently, Raut and colleagues also suggested the involvement of fibro-slime proteins in facilitating close contact of CBMs and GHs to cellulose for cellulose degradation after discovering that localization of these proteins on the cell surface increased when *F*. *succinogenes* was exposed to cellulose [25]. Our results also corroborate these findings, as we found eight of the genes encoding for fibro-slime proteins had higher expression on cellulose, relative to the glucose (Fig 1). Moreover, all seven of the fibro-slime proteins observed in our subcellular fractionation were present in the extracellular medium with four present in the outer membrane and one in the periplasm, but with lower abundance than those in the extracellular medium (Fig 6). These findings support the proposed mechanism of fibro-slime proteins being preferentially extracted into the extracellular medium or loosely affiliated with the outer membrane for the purpose of binding to cellulose.

Following secretion into the external medium, endoglucanases cleaves internal bonds of the cellulose chain with the aid of CBMs (Fisuc_1763, 1764, 1767, 1790, 1793, 2478), freeing up cellodextrins that would be transported into the periplasm for further degradation by CAZymes found to be specific to the periplasm. For example, Fisuc_3111, a predicted β-glucanase and Fisuc_1531, a predicted GH9 cellulase [3], were both observed in the periplasm. In addition, we also found the presence of a cellodextrin phosphorylase (Fisuc_2900) in the periplasmic space, indicating that cellodextrins transported into the cell could be phosphorylated and cleaved for direct entry into glycolysis, as has been previously suggested (3). This cellodextrin phosphorylase was found exclusively in cells grown on cellulose [25] and is also known to degrade and synthesize cellodextrins reversibly [28]. Although transporters for cellodextrins within *F*. *succinogenes* have not been specifically delineated, we found type II and III secretion systems exhibiting larger relative gene expression in the cellulose cultures, relative to the glucose cultures (Fig 3), that may serve a role in providing active transport. We did not observe these transporters in our proteomics data and evidence for this hypothesis is based solely on our RNA expression data.

An important question raised by the data presented here is the fate of the vesicles that are extracellularly released by *F*. *succinogenes*. Forsberg and colleagues suggested the use of these vesicles in cellulose degradation [5], and under this model, vesicles containing cellulases are released from the outer membrane via bleb formation. They posited that these vesicles could be released into the area between the cellulose and the cell, and in some instances, vesicles might also be generated by unattached cells. Additionally, they saw vesicles adhering to cellulose and in the extracellular medium. In this study, we did not observe vesicles attached to cellulose particles (Fig 7), or within the grooves between the cellulose and the cell, where cellulose degradation is thought to occur. However, we did observe vesicles either attached to the outer membrane or in the extracellular medium, but only in the stationary cultures. This is consistent with a previous report showing that the presence of cellulose induces vesicle formation [8]. They also found an absence of vesicles in younger cultures, suggesting that they do not directly impact the cellulolytic activity of *F*. *succinogenes* at that phase in growth. They further postulated that vesicle formation may be associated with aging cells. Based on these observations and the data presented here, we posit that early phase cells secrete cellulases into the external environment and possibly embed cellulases into their outer membrane to facilitate cellulose degradation (Fig 4). Pilin and fibro-slime proteins would be used to strongly anchor the cell to cellulose, thereby bringing cellulose fibers closer to these membrane-bound cellulases. As cellulose is degraded, the resulting cellodextrins would be transported into the cell for further breakdown and metabolized for ATP production via substrate-level phosphorylation through glycolysis. As attached cells reach stationary phase, the outer membrane blebs off and forms vesicles in order to continue cellulose degradation in a nutritionally limited environment [29]. Because *F*. *succinogenes* cells are non-motile, they would presumably continue to bore into the cellulose fiber at their initial site of attachment, and the formation of vesicles might help them remain attached to the fiber as it is degraded. This proposed mechanism would help explain the grooves in cellulose fibers that we observed in our TEM images (Fig 7). The use of vesicles during stationary phase may act as a delivery mechanism for a concentrated cocktail of cellulases to actively degrade cellulose at the site of attachment, as has been suggested (5).

This model also supports previous proposals regarding the role of *F*. *succinogenes* within the rumen ecosystem. These bacteria are known to synergistically work with other ruminal microbes to increase substrate degradation and volatile fatty acid production. A key aspect of this interaction includes the production of xylanases but the inability of this organism to utilize the resulting breakdown products [30, 31]. Presumably, these nutrients would be made available to other ruminal microbes for growth. The formation of vesicles by aging *F*. *succinogenes* cells may also play a similar role, as the free release of cellulolytic vesicles into the extracellular matrix could result in the production of mono- and di-saccharides to organisms lacking cellulose degrading enzymes [5].

## Supporting Information

S1 Table *Fibrobacter succinogenes* S85 transcripts with differential expression (q-value < 0.01). (DOCX)

S2 Table Clusters of orthologous (COGs) analysis for *Fibrobacter succinogenes* S85. (DOCX)

S3 Table Proteins observed in the subcellular fractionation of *Fibrobacter succinogenes* S85. (DOCX)

S4 Table Total and aligned RNA-Seq Reads. (DOCX)

S5 Table Components and conentrations of defined medium for *Fibrobacter succinogenes* S85. (DOCX)
