# Supplementary material for: Evaluating Models of Cellulose Degradation by Fibrobacter succinogenes S85
Source: PLoS One. 2015 Dec 2;10(12):e0143809. doi: 10.1371/journal.pone.0143809 (PMC4668043; doi:10.1371/journal.pone.0143809)
Supplement: S1 Table — (DOCX) [file pone.0143809.s001.docx]

| Protein ID | Description | Cellulose RPKM MEAN | Cellulose STDEV | Glucose RPKM MEAN | Glucose STDEV | qValue Cellulose vs Glucose | CAZy |
| --- | --- | --- | --- | --- | --- | --- | --- |
| Fisuc_0002 | diaminopimelate dehydrogenase | 313 | 88 | 479 | 40 | 4.20E-03 |  |
| Fisuc_0003 | hypothetical protein | 124 | 12 | 8 | 2 | 0 |  |
| Fisuc_0004 | exporter of the RND superfamily protein-like protein | 103 | 14 | 6 | 2 | 0 |  |
| Fisuc_0005 | hypothetical protein | 102 | 14 | 8 | 2 | 0 |  |
| Fisuc_0006 | hypothetical protein | 134 | 11 | 66 | 4 | 2.21E-23 |  |
| Fisuc_0010 | hypothetical protein | 95 | 10 | 64 | 5 | 5.73E-09 |  |
| Fisuc_0011 | hypothetical protein | 138 | 11 | 116 | 8 | 6.98E-03 |  |
| Fisuc_0012* | hypothetical protein | 101 | 4 | 65 | 5 | 2.61E-12 |  |
| Fisuc_0013 | hypothetical protein | 202 | 34 | 88 | 6 | 2.17E-28 |  |
| Fisuc_0015* | Holliday junction resolvase YqgF | 185 | 16 | 144 | 6 | 9.25E-05 |  |
| Fisuc_0016 | GTP-binding protein TypA | 382 | 21 | 836 | 11 | 1.10E-10 |  |
| Fisuc_0017 | phosphomethylpyrimidine kinase | 121 | 4 | 71 | 3 | 3.33E-15 |  |
| Fisuc_0018 | hypothetical protein | 83 | 6 | 29 | 3 | 3.86E-62 |  |
| Fisuc_0019 | diguanylate cyclase | 131 | 5 | 84 | 1 | 2.42E-12 |  |
| Fisuc_0021 | hypothetical protein | 62 | 22 | 94 | 14 | 6.34E-06 |  |
| Fisuc_0022 | bis(5'-nucleosyl)-tetraphosphatase | 51 | 19 | 114 | 12 | 2.98E-27 |  |
| Fisuc_0023 | FKBP-type peptidylprolyl isomerase | 219 | 13 | 401 | 28 | 3.08E-11 |  |
| Fisuc_0024 | ribose 5-phosphate isomerase | 284 | 11 | 427 | 31 | 1.30E-05 |  |
| Fisuc_0025 | alpha/beta hydrolase | 75 | 18 | 20 | 1 | 2.69E-182 |  |
| Fisuc_0026 | hypothetical protein | 162 | 33 | 125 | 1 | 1.09E-03 |  |
| Fisuc_0027 | hypothetical protein | 43 | 7 | 32 | 1 | 3.48E-08 |  |
| Fisuc_0028 | hypothetical protein | 22 | 2 | 19 | 3 | 3.95E-05 |  |
| Fisuc_0029 | lipoprotein | 27 | 4 | 65 | 22 | 5.34E-33 |  |
| Fisuc_0030 | hypothetical protein | 17 | 2 | 47 | 13 | 3.34E-45 |  |
| Fisuc_0033 | hypothetical protein | 685 | 76 | 1423 | 67 | 2.53E-12 |  |
| Fisuc_0034 | hypothetical protein | 147 | 5 | 236 | 21 | 5.69E-07 |  |
| Fisuc_0035 | hypothetical protein | 49 | 5 | 30 | 3 | 1.66E-20 |  |
| Fisuc_0036 | peptidase U32 | 73 | 7 | 56 | 3 | 3.41E-05 |  |
| Fisuc_0037 | hypothetical protein | 106 | 11 | 161 | 8 | 6.45E-06 |  |
| Fisuc_0038 | hypothetical protein | 117 | 14 | 200 | 19 | 7.65E-09 |  |
| Fisuc_0039 | TonB family protein | 63 | 22 | 123 | 14 | 2.35E-18 |  |
| Fisuc_0040 | biopolymer transport protein ExbD/TolR | 83 | 26 | 154 | 19 | 1.66E-13 |  |
| Fisuc_0041 | MotA/TolQ/ExbB proton channel | 49 | 15 | 104 | 17 | 1.92E-20 |  |
| Fisuc_0042 | MotA/TolQ/ExbB proton channel | 180 | 29 | 289 | 35 | 1.92E-04 |  |
| Fisuc_0043 | hypothetical protein | 272 | 14 | 460 | 45 | 5.16E-10 |  |
| Fisuc_0044* | pseudouridine synthase | 55 | 5 | 72 | 10 | 3.66E-03 |  |
| Fisuc_0050 | UbiA prenyltransferase | 163 | 17 | 121 | 7 | 1.31E-04 |  |
| Fisuc_0052 | NAD-dependent epimerase/dehydratase | 684 | 49 | 259 | 29 | 5.18E-58 |  |
| Fisuc_0053 | D-alanine--D-alanine ligase | 297 | 14 | 246 | 13 | 1.54E-04 |  |
| Fisuc_0055 | ribosomal-protein-alanine acetyltransferase | 211 | 11 | 174 | 9 | 3.95E-03 |  |
| Fisuc_0056 | hypothetical protein | 141 | 15 | 107 | 3 | 1.96E-03 |  |
| Fisuc_0057 | cellulase | 123 | 37 | 55 | 3 | 2.36E-24 | YES |
| Fisuc_0058 | hypothetical protein | 141 | 9 | 107 | 14 | 1.85E-06 |  |
| Fisuc_0060 | chaperonin Cpn10 | 453 | 107 | 1602 | 237 | 4.51E-82 |  |
| Fisuc_0061 | chaperonin GroEL | 778 | 213 | 2841 | 465 | 1.10E-19 |  |
| Fisuc_0065 | peptidase M23 | 403 | 19 | 347 | 13 | 6.05E-06 |  |
| Fisuc_0066 | hypothetical protein | 2213 | 198 | 759 | 34 | 1.91E-45 |  |
| Fisuc_0068 | 3-isopropylmalate dehydratase small subunit | 438 | 10 | 737 | 24 | 3.76E-06 |  |
| Fisuc_0072 | ErfK/YbiS/YcfS/YnhG family protein | 21 | 3 | 19 | 3 | 9.21E-04 |  |
| Fisuc_0074 | hypothetical protein | 74 | 5 | 68 | 5 | 3.83E-03 |  |
| Fisuc_0075 | N-acetyltransferase GCN5 | 65 | 5 | 57 | 2 | 1.08E-03 |  |
| Fisuc_0076 | hypothetical protein | 64 | 5 | 42 | 2 | 7.88E-09 |  |
| Fisuc_0077 | membrane-flanked domain-containing protein | 47 | 7 | 28 | 2 | 9.63E-24 |  |
| Fisuc_0078 | hypothetical protein | 254 | 16 | 63 | 9 | 1.57E-207 |  |
| Fisuc_0079 | Superoxide dismutase | 502 | 101 | 16 | 3 | 0 |  |
| Fisuc_0080 | hypothetical protein | 512 | 92 | 350 | 8 | 2.35E-06 |  |
| Fisuc_0081 | hypothetical protein | 436 | 65 | 330 | 10 | 8.09E-04 |  |
| Fisuc_0084 | hypothetical protein | 90 | 7 | 56 | 2 | 1.42E-11 |  |
| Fisuc_0085 | hypothetical protein | 180 | 15 | 79 | 3 | 6.92E-50 |  |
| Fisuc_0086 | hypothetical protein | 166 | 11 | 64 | 8 | 6.85E-49 |  |
| Fisuc_0087 | hypothetical protein | 8 | 1 | 16 | 5 | 4.52E-11 |  |
| Fisuc_0088 | hypothetical protein | 10 | 1 | 24 | 3 | 2.52E-33 |  |
| Fisuc_0089 | hypothetical protein | 43 | 4 | 17 | 2 | 3.00E-77 |  |
| Fisuc_0090 | anaerobic ribonucleoside-triphosphate reductase | 9 | 2 | 7 | 1 | 9.02E-09 |  |
| Fisuc_0091 | hypothetical protein | 6 | 2 | 15 | 2 | 9.15E-24 |  |
| Fisuc_0093 | von Willebrand factor type A | 10 | 3 | 21 | 4 | 2.56E-20 |  |
| Fisuc_0094 | hypothetical protein | 11 | 2 | 24 | 3 | 3.76E-14 |  |
| Fisuc_0103* | redoxin domain-containing protein | 89 | 16 | 128 | 6 | 1.12E-04 |  |
| Fisuc_0104 | hypothetical protein | 94 | 13 | 123 | 4 | 2.32E-03 |  |
| Fisuc_0105 | dihydroorotate oxidase | 174 | 11 | 243 | 2 | 1.38E-03 |  |
| Fisuc_0106 | peptidase M20 | 132 | 6 | 83 | 10 | 6.61E-09 |  |
| Fisuc_0107* | silent information regulator protein Sir2 | 188 | 11 | 102 | 6 | 2.49E-17 |  |
| Fisuc_0108 | hypothetical protein | 165 | 48 | 50 | 4 | 8.88E-88 |  |
| Fisuc_0111 | hypothetical protein | 199 | 6 | 142 | 12 | 6.45E-06 |  |
| Fisuc_0112 | radical SAM protein | 134 | 16 | 56 | 5 | 1.99E-40 |  |
| Fisuc_0113 | hypothetical protein | 270 | 35 | 104 | 2 | 2.44E-47 |  |
| Fisuc_0114 | hypothetical protein | 63 | 3 | 28 | 1 | 6.10E-29 |  |
| Fisuc_0115 | exporter of the RND superfamily protein-like protein | 124 | 8 | 95 | 5 | 5.85E-06 |  |
| Fisuc_0118* | serine/threonine protein kinase | 116 | 4 | 165 | 3 | 9.32E-04 |  |
| Fisuc_0123 | diguanylate cyclase/phosphodiesterase | 440 | 41 | 106 | 14 | 1.63E-155 |  |
| Fisuc_0124 | hypothetical protein | 248 | 27 | 53 | 4 | 0 |  |
| Fisuc_0125 | hypothetical protein | 98 | 11 | 25 | 3 | 9.81E-195 |  |
| Fisuc_0126 | hypothetical protein | 42 | 2 | 66 | 5 | 2.16E-06 |  |
| Fisuc_0129 | hypothetical protein | 91 | 10 | 58 | 4 | 2.14E-09 |  |
| Fisuc_0130 | cobalamin synthesis protein P47K | 225 | 15 | 354 | 25 | 1.66E-03 |  |
| Fisuc_0131 | hypothetical protein | 249 | 46 | 105 | 5 | 3.19E-41 |  |
| Fisuc_0132 | ankyrin | 90 | 28 | 26 | 2 | 4.15E-109 |  |
| Fisuc_0133 | hypothetical protein | 165 | 59 | 71 | 9 | 3.79E-33 |  |
| Fisuc_0134 | von Willebrand factor type A | 143 | 57 | 72 | 5 | 6.88E-24 |  |
| Fisuc_0135 | serine/threonine protein kinase | 45 | 28 | 28 | 2 | 4.54E-10 |  |
| Fisuc_0136 | hypothetical protein | 56 | 5 | 237 | 48 | 3.87E-159 |  |
| Fisuc_0137 | anthranilate synthase | 530 | 23 | 420 | 25 | 7.99E-08 |  |
| Fisuc_0139 | hypothetical protein | 109 | 6 | 71 | 4 | 4.32E-09 |  |
| Fisuc_0143* | nitrite and sulfite reductase 4Fe-4S region | 132 | 17 | 226 | 28 | 8.74E-09 |  |
| Fisuc_0146 | sulfate adenylyltransferase subunit 2 | 49 | 11 | 71 | 6 | 2.66E-05 |  |
| Fisuc_0147 | sulfate adenylyltransferase, large subunit | 56 | 10 | 79 | 6 | 6.33E-03 |  |
| Fisuc_0148 | hypothetical protein | 52 | 10 | 162 | 41 | 3.63E-74 |  |
| Fisuc_0149 | sulfate ABC transporter, periplasmic sulfate-binding protein | 66 | 17 | 149 | 31 | 1.38E-23 |  |
| Fisuc_0150 | sulfate ABC transporter, inner membrane subunit CysT | 53 | 9 | 120 | 26 | 2.66E-30 |  |
| Fisuc_0151 | sulfate ABC transporter, inner membrane subunit CysW | 67 | 10 | 98 | 17 | 4.98E-06 |  |
| Fisuc_0153 | hypothetical protein | 24 | 4 | 58 | 15 | 3.79E-30 |  |
| Fisuc_0154 | metal dependent phophohydrolase | 38 | 4 | 98 | 18 | 3.03E-36 |  |
| Fisuc_0155 | O-acetylhomoserine/O-acetylserine sulfhydrylase | 15 | 4 | 40 | 12 | 7.45E-44 |  |
| Fisuc_0156* | hypothetical protein | 14 | 3 | 25 | 7 | 2.77E-07 |  |
| Fisuc_0157 | cupin | 13 | 4 | 21 | 4 | 7.91E-07 |  |
| Fisuc_0158 | hypothetical protein | 68 | 4 | 206 | 24 | 4.49E-64 |  |
| Fisuc_0159 | biotin synthase | 88 | 6 | 189 | 22 | 2.53E-18 |  |
| Fisuc_0160 | hypothetical protein | 43 | 9 | 98 | 16 | 1.00E-25 |  |
| Fisuc_0162 | O-acetylhomoserine aminocarboxypropyltransferase | 185 | 20 | 579 | 29 | 5.46E-66 |  |
| Fisuc_0163 | SirA family protein | 399 | 74 | 1006 | 35 | 8.21E-31 |  |
| Fisuc_0164 | thiamine biosynthesis protein ThiS | 381 | 80 | 912 | 25 | 1.19E-26 |  |
| Fisuc_0165 | UBA/THIF-type NAD/FAD binding protein | 394 | 112 | 846 | 47 | 8.66E-14 |  |
| Fisuc_0166 | Mov34/MPN/PAD-1 family protein | 314 | 84 | 561 | 25 | 1.43E-10 |  |
| Fisuc_0168 | hypothetical protein | 292 | 50 | 204 | 18 | 2.25E-06 |  |
| Fisuc_0169 | RelE/StbE family addiction module toxin | 247 | 46 | 168 | 8 | 2.77E-07 |  |
| Fisuc_0170 | thiazole biosynthesis adenylyltransferase ThiF | 266 | 24 | 486 | 33 | 3.82E-08 |  |
| Fisuc_0174 | Pectate lyase/Amb allergen | 227 | 175 | 11 | 1 | 0 | YES |
| Fisuc_0175 | hypothetical protein | 63 | 26 | 0 | 0 | 0 |  |
| Fisuc_0176 | indole-3-glycerol-phosphate synthase., phosphoribosylanthranilate isomerase | 40 | 4 | 58 | 3 | 5.28E-05 |  |
| Fisuc_0177* | inorganic diphosphatase | 10 | 2 | 6 | 2 | 4.49E-17 |  |
| Fisuc_0180 | tryptophan synthase subunit alpha | 266 | 16 | 561 | 76 | 2.93E-19 |  |
| Fisuc_0181* | aconitate hydratase | 147 | 2 | 252 | 1 | 2.63E-04 |  |
| Fisuc_0186* | endoribonuclease L-PSP | 393 | 39 | 805 | 67 | 7.61E-19 |  |
| Fisuc_0188* | DNA mismatch repair protein MutS | 60 | 8 | 96 | 10 | 3.63E-06 |  |
| Fisuc_0189* | chromosome segregation and condensation protein ScpB | 75 | 21 | 108 | 12 | 1.70E-05 |  |
| Fisuc_0190 | glutamate 5-kinase | 65 | 16 | 93 | 10 | 4.80E-04 |  |
| Fisuc_0194 | ABC transporter | 46 | 4 | 77 | 8 | 1.53E-08 |  |
| Fisuc_0195 | transport system permease | 25 | 13 | 60 | 11 | 1.20E-30 |  |
| Fisuc_0196 | hypothetical protein | 73 | 22 | 149 | 19 | 1.81E-17 |  |
| Fisuc_0197 | OmpA/MotB domain-containing protein | 93 | 14 | 173 | 13 | 4.45E-16 |  |
| Fisuc_0198 | TolB domain-containing protein | 94 | 8 | 159 | 17 | 2.65E-08 |  |
| Fisuc_0199 | hypothetical protein | 82 | 7 | 151 | 19 | 2.54E-09 |  |
| Fisuc_0202 | hypothetical protein | 1623 | 111 | 3424 | 290 | 1.06E-07 |  |
| Fisuc_0204* | Deoxyribonuclease I | 92 | 11 | 35 | 4 | 3.10E-71 |  |
| Fisuc_0207 | glycoside hydrolase family protein | 142 | 3 | 42 | 3 | 1.94E-100 | YES |
| Fisuc_0208* | Crp family transcriptional regulator | 116 | 10 | 46 | 3 | 2.52E-45 |  |
| Fisuc_0210 | hypothetical protein | 7 | 1 | 5 | 0 | 1.52E-04 |  |
| Fisuc_0212 | protein tyrosine/serine phosphatase | 118 | 14 | 96 | 12 | 2.46E-03 |  |
| Fisuc_0214 | glycosyltransferase 28 domain protein | 59 | 10 | 97 | 4 | 8.20E-07 | YES |
| Fisuc_0216 | hypothetical protein | 59 | 5 | 11 | 2 | 0 |  |
| Fisuc_0217 | hypothetical protein | 73 | 16 | 13 | 1 | 0 |  |
| Fisuc_0218 | hypothetical protein | 102 | 18 | 43 | 5 | 2.77E-61 |  |
| Fisuc_0219 | radical SAM protein | 109 | 14 | 45 | 4 | 2.93E-40 |  |
| Fisuc_0222 | hypothetical protein | 255 | 15 | 194 | 5 | 6.29E-04 |  |
| Fisuc_0223 | Outer membrane protein/protective antigen OMA87-like protein | 37 | 4 | 24 | 2 | 9.24E-10 |  |
| Fisuc_0226 | hypothetical protein | 381 | 31 | 331 | 13 | 5.24E-03 |  |
| Fisuc_0227 | GTPase-like protein | 758 | 17 | 657 | 31 | 2.35E-05 |  |
| Fisuc_0228 | 30S ribosomal protein S20 | 3046 | 378 | 7774 | 780 | 3.78E-14 |  |
| Fisuc_0231 | phosphoesterase RecJ domain-containing protein | 175 | 10 | 134 | 10 | 4.88E-05 |  |
| Fisuc_0238 | hypothetical protein | 20 | 4 | 55 | 9 | 1.02E-41 |  |
| Fisuc_0242 | MORN variant repeat protein | 143 | 14 | 102 | 7 | 1.41E-04 |  |
| Fisuc_0243* | iron-containing alcohol dehydrogenase | 57 | 14 | 17 | 2 | 2.23E-155 |  |
| Fisuc_0248 | type II and III secretion system protein | 544 | 38 | 424 | 11 | 9.12E-08 |  |
| Fisuc_0250 | hypothetical protein | 484 | 46 | 321 | 5 | 1.58E-08 |  |
| Fisuc_0251 | fimbrial assembly family protein | 544 | 62 | 358 | 22 | 9.63E-10 |  |
| Fisuc_0252 | Tfp pilus assembly protein ATPase PilM-like protein | 645 | 41 | 439 | 11 | 2.83E-12 |  |
| Fisuc_0255 | aspartate carbamoyltransferase | 388 | 16 | 300 | 16 | 6.53E-05 |  |
| Fisuc_0257 | cyclophilin type peptidyl-prolyl cis-trans isomerase | 513 | 31 | 340 | 13 | 1.49E-08 |  |
| Fisuc_0261 | hypothetical protein | 36 | 4 | 22 | 2 | 1.95E-06 |  |
| Fisuc_0263* | adenine-specific DNA-methyltransferase | 154 | 12 | 126 | 2 | 8.09E-04 |  |
| Fisuc_0264 | KilA, N-terminal/APSES-type HTH DNA-binding domain-containing protein | 13 | 2 | 12 | 1 | 6.53E-03 |  |
| Fisuc_0265 | Heat shock protein Hsp90-like protein | 323 | 88 | 1315 | 200 | 3.62E-48 |  |
| Fisuc_0267 | hypothetical protein | 2 | 1 | 5 | 2 | 5.51E-11 |  |
| Fisuc_0268* | DeoR family transcriptional regulator | 12 | 1 | 11 | 1 | 1.59E-05 |  |
| Fisuc_0269 | pyridoxal biosynthesis lyase PdxS | 51 | 15 | 88 | 28 | 3.42E-11 |  |
| Fisuc_0270 | glutamine amidotransferase subunit PdxT | 164 | 13 | 120 | 23 | 1.51E-05 |  |
| Fisuc_0271 | hypothetical protein | 249 | 4 | 182 | 29 | 1.51E-05 |  |
| Fisuc_0272 | hypothetical protein | 209 | 11 | 335 | 19 | 2.32E-03 |  |
| Fisuc_0273 | FAD dependent oxidoreductase | 105 | 8 | 147 | 5 | 1.80E-03 |  |
| Fisuc_0274 | hypothetical protein | 55 | 14 | 77 | 6 | 5.37E-03 |  |
| Fisuc_0275* | pseudouridine synthase | 54 | 8 | 73 | 3 | 3.24E-03 |  |
| Fisuc_0276* | UvrD/REP helicase | 16 | 1 | 23 | 2 | 9.14E-04 |  |
| Fisuc_0277 | hypothetical protein | 102 | 16 | 7 | 1 | 0 |  |
| Fisuc_0278 | hypothetical protein | 79 | 6 | 45 | 1 | 5.53E-30 |  |
| Fisuc_0279 | hypothetical protein | 86 | 9 | 50 | 6 | 7.45E-25 |  |
| Fisuc_0284 | hypothetical protein | 207 | 7 | 163 | 6 | 1.39E-04 |  |
| Fisuc_0286* | DNA polymerase III subunits gamma and tau | 355 | 15 | 316 | 6 | 2.04E-05 |  |
| Fisuc_0288 | outer membrane efflux protein | 267 | 12 | 586 | 35 | 2.82E-15 |  |
| Fisuc_0289 | RND family efflux transporter MFP subunit | 376 | 11 | 783 | 62 | 9.23E-13 |  |
| Fisuc_0290 | acriflavin resistance protein | 372 | 14 | 838 | 33 | 1.92E-05 |  |
| Fisuc_0291 | type II secretion system protein E | 43 | 6 | 23 | 4 | 5.73E-28 |  |
| Fisuc_0292* | zinc finger CHC2-family protein | 98 | 11 | 74 | 8 | 4.55E-04 |  |
| Fisuc_0293 | UDP-N-acetylenolpyruvoylglucosamine reductase | 66 | 7 | 49 | 5 | 1.21E-04 |  |
| Fisuc_0295 | VanZ family protein | 168 | 31 | 114 | 6 | 2.50E-07 |  |
| Fisuc_0296 | FecR protein | 479 | 40 | 159 | 25 | 2.11E-82 |  |
| Fisuc_0298 | FGGY-like carbohydrate kinase | 222 | 28 | 178 | 4 | 1.59E-04 |  |
| Fisuc_0300 | hypothetical protein | 168 | 11 | 58 | 5 | 3.61E-117 |  |
| Fisuc_0304 | hypothetical protein | 902 | 51 | 636 | 22 | 1.96E-06 |  |
| Fisuc_0306 | hypothetical protein | 95 | 22 | 192 | 17 | 3.86E-18 |  |
| Fisuc_0307 | hypothetical protein | 156 | 23 | 240 | 1 | 5.80E-06 |  |
| Fisuc_0310 | hypothetical protein | 18 | 3 | 11 | 1 | 3.63E-19 |  |
| Fisuc_0311 | hypothetical protein | 31 | 2 | 24 | 2 | 6.30E-08 |  |
| Fisuc_0312 | hypothetical protein | 56 | 4 | 43 | 3 | 6.83E-06 |  |
| Fisuc_0313 | ketol-acid reductoisomerase | 843 | 209 | 1547 | 83 | 6.00E-03 |  |
| Fisuc_0314 | hypothetical protein | 36 | 5 | 67 | 5 | 3.50E-13 |  |
| Fisuc_0316 | short-chain dehydrogenase/reductase SDR | 37 | 4 | 30 | 4 | 5.24E-06 |  |
| Fisuc_0317* | transcriptional regulator, XRE family | 153 | 26 | 69 | 5 | 2.84E-44 |  |
| Fisuc_0318 | beta-lactamase | 160 | 14 | 60 | 6 | 1.85E-57 |  |
| Fisuc_0319 | hypothetical protein | 58 | 3 | 53 | 3 | 5.35E-03 |  |
| Fisuc_0320 | hypothetical protein | 131 | 36 | 34 | 5 | 1.00E-221 |  |
| Fisuc_0321 | N-acetyltransferase GCN5 | 213 | 38 | 60 | 4 | 5.80E-160 |  |
| Fisuc_0322 | hypothetical protein | 300 | 51 | 28 | 6 | 0 |  |
| Fisuc_0323 | glycoside hydrolase family protein | 70 | 10 | 13 | 1 | 0 | YES |
| Fisuc_0324* | RelA/SpoT domain-containing protein | 42 | 4 | 15 | 1 | 2.41E-103 |  |
| Fisuc_0325 | hypothetical protein | 20 | 4 | 10 | 2 | 9.08E-22 |  |
| Fisuc_0326 | carboxynorspermidine decarboxylase | 115 | 15 | 157 | 4 | 2.04E-03 |  |
| Fisuc_0327 | hypothetical protein | 79 | 18 | 128 | 13 | 1.25E-07 |  |
| Fisuc_0331 | pentapeptide repeat-containing protein | 2575 | 72 | 1715 | 206 | 1.61E-04 |  |
| Fisuc_0332 | DNA repair protein RadA | 37 | 5 | 54 | 4 | 7.34E-06 |  |
| Fisuc_0333 | response regulator receiver modulated diguanylate cyclase/phosphodiesterase | 263 | 58 | 68 | 19 | 1.10E-149 |  |
| Fisuc_0334 | short-chain dehydrogenase/reductase SDR | 318 | 44 | 26 | 2 | 0 |  |
| Fisuc_0335* | transcriptional regulator, XRE family | 32 | 19 | 5 | 1 | 0 |  |
| Fisuc_0338 | SEC-C motif domain protein | 623 | 27 | 1126 | 21 | 1.85E-05 |  |
| Fisuc_0339 | N-acetyl-gamma-glutamyl-phosphate reductase | 471 | 6 | 806 | 8 | 2.68E-06 |  |
| Fisuc_0341 | hypothetical protein | 87 | 17 | 63 | 5 | 2.01E-05 |  |
| Fisuc_0342 | hypothetical protein | 32 | 11 | 15 | 1 | 1.86E-25 |  |
| Fisuc_0343 | hypothetical protein | 19 | 5 | 15 | 4 | 8.73E-05 |  |
| Fisuc_0344 | TonB family protein | 70 | 2 | 109 | 2 | 2.17E-05 |  |
| Fisuc_0345 | hypothetical protein | 64 | 7 | 89 | 3 | 9.78E-04 |  |
| Fisuc_0346 | hypothetical protein | 67 | 8 | 108 | 9 | 3.65E-09 |  |
| Fisuc_0347 | GTP-binding protein Obg/CgtA | 67 | 25 | 113 | 9 | 2.08E-08 |  |
| Fisuc_0348* | histone family protein DNA-binding protein | 595 | 62 | 448 | 39 | 1.11E-04 |  |
| Fisuc_0349 | SsrA-binding protein | 478 | 40 | 353 | 18 | 1.77E-04 |  |
| Fisuc_0350 | N-acetylmuramoyl-L-alanine amidase | 266 | 23 | 194 | 2 | 7.57E-06 |  |
| Fisuc_0351 | hypothetical protein | 69 | 3 | 34 | 2 | 1.20E-25 |  |
| Fisuc_0352 | hypothetical protein | 35 | 4 | 10 | 1 | 9.05E-155 |  |
| Fisuc_0353 | hypothetical protein | 35 | 6 | 7 | 1 | 7.37E-209 |  |
| Fisuc_0354 | hypothetical protein | 56 | 7 | 12 | 2 | 0 |  |
| Fisuc_0357 | hypothetical protein | 433 | 35 | 654 | 59 | 7.85E-03 |  |
| Fisuc_0362 | endo-1,4-beta-xylanase | 141 | 26 | 21 | 2 | 0 | YES |
| Fisuc_0366 | hypothetical protein | 9 | 1 | 29 | 2 | 2.76E-72 |  |
| Fisuc_0367 | hypothetical protein | 8 | 1 | 32 | 3 | 5.47E-116 |  |
| Fisuc_0368 | hypothetical protein | 20 | 1 | 118 | 21 | 0 |  |
| Fisuc_0369 | hypothetical protein | 574 | 60 | 376 | 31 | 4.20E-11 |  |
| Fisuc_0370 | hypothetical protein | 31 | 3 | 2 | 1 | 0 |  |
| Fisuc_0371* | RNA methylase | 30 | 4 | 18 | 1 | 8.21E-17 |  |
| Fisuc_0377 | fibro-slime family protein | 1022 | 239 | 270 | 15 | 4.46E-81 |  |
| Fisuc_0380 | hypothetical protein | 140 | 16 | 107 | 9 | 7.39E-09 |  |
| Fisuc_0382 | hypothetical protein | 1559 | 101 | 1113 | 76 | 4.96E-07 |  |
| Fisuc_0383 | dihydroneopterin aldolase | 874 | 81 | 551 | 48 | 1.20E-10 |  |
| Fisuc_0384 | Mg chelatase subunit ChlI | 421 | 150 | 35 | 3 | 0 |  |
| Fisuc_0385 | hypothetical protein | 780 | 273 | 13 | 2 | 0 |  |
| Fisuc_0389 | alpha-N-arabinofuranosidase | 54 | 23 | 10 | 1 | 0 | YES |
| Fisuc_0391* | Fe-S type, tartrate/fumarate subfamily hydro-lyase subunit beta | 638 | 32 | 598 | 48 | 2.55E-03 |  |
| Fisuc_0393 | glycoside hydrolase family protein | 266 | 25 | 149 | 5 | 1.85E-14 | YES |
| Fisuc_0394 | glycoside hydrolase family protein | 94 | 16 | 63 | 7 | 1.13E-06 | YES |
| Fisuc_0395 | integral membrane sensor signal transduction histidine kinase | 97 | 8 | 63 | 4 | 2.55E-09 |  |
| Fisuc_0396 | response regulator receiver protein | 1894 | 88 | 1330 | 41 | 6.71E-11 |  |
| Fisuc_0400 | transketolase | 246 | 45 | 465 | 31 | 7.03E-07 |  |
| Fisuc_0402 | family 2 glycosyl transferase | 127 | 16 | 226 | 11 | 3.91E-10 | YES |
| Fisuc_0403 | guanylate kinase | 49 | 13 | 83 | 8 | 3.80E-09 |  |
| Fisuc_0414 | hypothetical protein | 458 | 32 | 253 | 14 | 1.65E-12 |  |
| Fisuc_0417 | hypothetical protein | 260 | 27 | 199 | 16 | 4.76E-07 |  |
| Fisuc_0418 | nitrilase/cyanide hydratase and apolipoprotein N-acyltransferase | 63 | 4 | 38 | 2 | 7.93E-17 |  |
| Fisuc_0419 | hypothetical protein | 358 | 40 | 220 | 8 | 6.65E-17 |  |
| Fisuc_0420 | hypothetical protein | 338 | 44 | 212 | 20 | 8.11E-11 |  |
| Fisuc_0421 | aminoglycoside phosphotransferase | 151 | 28 | 83 | 1 | 5.73E-12 |  |
| Fisuc_0422 | Serine O-acetyltransferase | 141 | 21 | 220 | 11 | 4.14E-06 |  |
| Fisuc_0425 | hypothetical protein | 48 | 2 | 74 | 4 | 7.98E-08 |  |
| Fisuc_0427 | hypothetical protein | 68 | 4 | 48 | 4 | 8.46E-06 |  |
| Fisuc_0428* | Na+/H+ antiporter NhaC-like protein | 109 | 11 | 74 | 7 | 2.69E-06 |  |
| Fisuc_0429 | hypothetical protein | 122 | 17 | 82 | 4 | 1.78E-15 |  |
| Fisuc_0433 | peptidoglycan-binding lysin domain protein | 416 | 11 | 338 | 21 | 5.72E-03 | YES |
| Fisuc_0434 | integral membrane sensor signal transduction histidine kinase | 138 | 16 | 114 | 5 | 4.37E-04 |  |
| Fisuc_0437 | family 5 extracellular solute-binding protein | 101 | 25 | 54 | 2 | 3.79E-13 |  |
| Fisuc_0444 | hypothetical protein | 185 | 8 | 244 | 16 | 7.77E-03 |  |
| Fisuc_0445 | haloacid dehalogenase domain-containing protein hydrolase | 18 | 2 | 32 | 2 | 1.12E-08 |  |
| Fisuc_0448 | hypothetical protein | 73 | 7 | 54 | 3 | 1.79E-04 |  |
| Fisuc_0449 | protein tyrosine/serine phosphatase | 9 | 1 | 8 | 2 | 8.33E-04 |  |
| Fisuc_0450 | hypothetical protein | 27 | 3 | 23 | 2 | 4.44E-04 |  |
| Fisuc_0453* | MiaB family RNA modification protein | 35 | 1 | 110 | 5 | 5.98E-72 |  |
| Fisuc_0454 | Sporulation domain protein | 104 | 1 | 153 | 19 | 9.04E-04 |  |
| Fisuc_0457 | hypothetical protein | 157 | 16 | 439 | 43 | 4.37E-47 |  |
| Fisuc_0458 | hypothetical protein | 120 | 6 | 71 | 2 | 2.41E-12 |  |
| Fisuc_0459 | hypothetical protein | 109 | 13 | 353 | 12 | 5.55E-67 |  |
| Fisuc_0463 | hypothetical protein | 725 | 31 | 546 | 16 | 3.72E-09 |  |
| Fisuc_0467 | hypothetical protein | 89 | 17 | 134 | 4 | 1.38E-06 |  |
| Fisuc_0469* | DEAD/DEAH box helicase | 133 | 16 | 278 | 7 | 2.85E-20 |  |
| Fisuc_0470* | methionine aminopeptidase | 245 | 11 | 502 | 9 | 9.43E-19 |  |
| Fisuc_0471 | glycoside hydrolase family protein | 84 | 3 | 48 | 2 | 1.01E-13 | YES |
| Fisuc_0472 | hypothetical protein | 93 | 12 | 45 | 3 | 1.77E-45 |  |
| Fisuc_0473 | hypothetical protein | 111 | 16 | 47 | 7 | 2.79E-69 |  |
| Fisuc_0474 | hypothetical protein | 568 | 26 | 255 | 32 | 2.95E-30 |  |
| Fisuc_0476 | MazG family protein | 47 | 8 | 73 | 3 | 7.66E-07 |  |
| Fisuc_0480 | RND family efflux transporter MFP subunit | 60 | 21 | 84 | 10 | 3.08E-04 |  |
| Fisuc_0483 | hypothetical protein | 717 | 64 | 481 | 31 | 3.92E-03 |  |
| Fisuc_0484* | 50S ribosomal protein L9 | 2611 | 82 | 5326 | 164 | 7.62E-04 |  |
| Fisuc_0485* | 30S ribosomal protein S18 | 3416 | 163 | 7176 | 347 | 3.88E-08 |  |
| Fisuc_0486* | 30S ribosomal protein S6 | 3773 | 156 | 8339 | 415 | 7.35E-06 |  |
| Fisuc_0489* | tryptophanyl-tRNA synthetase | 296 | 15 | 460 | 3 | 2.32E-03 |  |
| Fisuc_0491 | 5-formyltetrahydrofolate cyclo-ligase | 192 | 14 | 143 | 9 | 1.05E-04 |  |
| Fisuc_0493 | spore coat protein CotH | 13 | 2 | 8 | 1 | 9.52E-25 |  |
| Fisuc_0497* | rubrerythrin | 369 | 48 | 83 | 13 | 6.48E-194 |  |
| Fisuc_0498 | hypothetical protein | 8 | 3 | 3 | 0 | 5.22E-17 | YES |
| Fisuc_0499 | hypothetical protein | 8 | 3 | 2 | 1 | 3.67E-49 |  |
| Fisuc_0506 | hypothetical protein | 157 | 11 | 120 | 8 | 1.80E-04 |  |
| Fisuc_0507 | hypothetical protein | 59 | 21 | 39 | 2 | 4.13E-11 |  |
| Fisuc_0508 | haloacid dehalogenase domain-containing protein hydrolase | 72 | 4 | 107 | 9 | 7.91E-05 |  |
| Fisuc_0515* | DNA topoisomerase IV subunit A | 106 | 5 | 175 | 16 | 1.78E-05 |  |
| Fisuc_0516 | hypothetical protein | 52 | 17 | 69 | 2 | 2.81E-03 |  |
| Fisuc_0517 | cell division ATP-binding protein FtsE | 84 | 27 | 177 | 14 | 1.48E-22 |  |
| Fisuc_0518 | PpiC-type peptidyl-prolyl cis-trans isomerase | 178 | 27 | 302 | 18 | 2.69E-10 |  |
| Fisuc_0519 | hypothetical protein | 186 | 8 | 311 | 16 | 7.31E-08 |  |
| Fisuc_0520* | helicase-associated domain-containing protein | 49 | 7 | 84 | 3 | 7.63E-11 |  |
| Fisuc_0521 | hypothetical protein | 48 | 10 | 90 | 4 | 1.86E-11 |  |
| Fisuc_0522 | thioesterase superfamily protein | 162 | 19 | 125 | 9 | 6.38E-05 |  |
| Fisuc_0523 | MATE efflux family protein | 15 | 0 | 8 | 1 | 2.20E-35 |  |
| Fisuc_0525* | DNA primase | 86 | 3 | 140 | 3 | 4.12E-08 |  |
| Fisuc_0526 | FeS assembly ATPase SufC | 64 | 18 | 123 | 11 | 6.38E-19 |  |
| Fisuc_0527 | SufBD protein | 61 | 8 | 112 | 11 | 2.71E-12 |  |
| Fisuc_0528 | hypothetical protein | 58 | 5 | 44 | 3 | 1.14E-03 |  |
| Fisuc_0529 | hypothetical protein | 72 | 10 | 111 | 8 | 1.42E-04 |  |
| Fisuc_0530 | sulfate transporter | 104 | 10 | 395 | 18 | 6.44E-103 |  |
| Fisuc_0531 | hypothetical protein | 147 | 14 | 99 | 6 | 1.07E-08 |  |
| Fisuc_0532 | hypothetical protein | 119 | 15 | 75 | 9 | 9.92E-10 |  |
| Fisuc_0535 | hypothetical protein | 15 | 4 | 11 | 3 | 7.97E-06 |  |
| Fisuc_0536 | hypothetical protein | 10 | 2 | 8 | 1 | 1.56E-04 |  |
| Fisuc_0537 | hypothetical protein | 4 | 1 | 2 | 1 | 3.90E-12 |  |
| Fisuc_0538 | hypothetical protein | 0 | 1 | 0 | 0 | 1.78E-13 |  |
| Fisuc_0540 | hypothetical protein | 5173 | 2286 | 35 | 10 | 0 |  |
| Fisuc_0541 | hypothetical protein | 1959 | 916 | 14 | 3 | 0 |  |
| Fisuc_0542 | hypothetical protein | 24 | 5 | 34 | 4 | 3.30E-04 |  |
| Fisuc_0544 | hypothetical protein | 19 | 2 | 30 | 1 | 1.22E-04 |  |
| Fisuc_0545 | aspartate racemase | 61 | 7 | 50 | 5 | 4.16E-03 |  |
| Fisuc_0547* | DNA topoisomerase IV subunit B | 169 | 8 | 269 | 7 | 3.29E-03 |  |
| Fisuc_0557 | hypothetical protein | 26 | 2 | 41 | 4 | 4.55E-07 |  |
| Fisuc_0560 | hypothetical protein | 48 | 6 | 35 | 3 | 9.50E-07 |  |
| Fisuc_0563 | cell division protein FtsA | 400 | 19 | 594 | 18 | 4.05E-03 |  |
| Fisuc_0567 | cell division protein FtsW | 179 | 24 | 269 | 6 | 2.01E-06 |  |
| Fisuc_0568 | UDP-N-acetylmuramoylalanyl-D-glutamyl-2,6-diaminopimelate/D-alanyl-D-alanyl ligase | 164 | 37 | 286 | 20 | 2.49E-11 |  |
| Fisuc_0573 | MraZ domain-containing protein | 265 | 46 | 732 | 102 | 4.18E-46 |  |
| Fisuc_0574 | hypothetical protein | 41 | 3 | 27 | 2 | 4.31E-12 |  |
| Fisuc_0576 | methylenetetrahydrofolate reductase | 284 | 23 | 788 | 44 | 8.78E-41 |  |
| Fisuc_0577 | OmpA/MotB domain-containing protein | 45 | 6 | 149 | 8 | 1.36E-71 |  |
| Fisuc_0578 | hypothetical protein | 170 | 3 | 437 | 14 | 1.71E-16 |  |
| Fisuc_0579 | hypothetical protein | 1834 | 122 | 1186 | 124 | 1.67E-15 |  |
| Fisuc_0580 | GLUG domain-containing protein | 117 | 3 | 64 | 4 | 1.08E-16 |  |
| Fisuc_0589 | hypothetical protein | 25 | 4 | 19 | 2 | 4.48E-08 |  |
| Fisuc_0590 | hypothetical protein | 518 | 15 | 368 | 17 | 6.81E-05 |  |
| Fisuc_0591 | hypothetical protein | 284 | 20 | 192 | 4 | 1.17E-06 |  |
| Fisuc_0592 | MATE efflux family protein | 17 | 1 | 28 | 2 | 1.70E-07 |  |
| Fisuc_0593* | GTP-binding protein YchF | 60 | 6 | 130 | 4 | 9.80E-19 |  |
| Fisuc_0595 | hypothetical protein | 88 | 11 | 57 | 4 | 7.83E-12 |  |
| Fisuc_0596* | excinuclease ABC subunit A | 25 | 2 | 51 | 3 | 9.92E-16 |  |
| Fisuc_0598 | hypothetical protein | 17 | 2 | 14 | 1 | 5.47E-09 |  |
| Fisuc_0599* | protein serine/threonine phosphatase | 131 | 9 | 24 | 3 | 0 |  |
| Fisuc_0600 | hypothetical protein | 1864 | 347 | 81 | 30 | 0 |  |
| Fisuc_0601 | hypothetical protein | 119 | 9 | 68 | 4 | 3.35E-14 |  |
| Fisuc_0602* | transcriptional regulator, TetR family | 32 | 7 | 18 | 2 | 2.85E-26 |  |
| Fisuc_0603* | N-acetyltransferase GCN5 | 23 | 3 | 16 | 2 | 2.39E-10 |  |
| Fisuc_0604 | chloramphenicol acetyltransferase | 20 | 3 | 15 | 1 | 4.42E-09 |  |
| Fisuc_0605 | hypothetical protein | 35 | 9 | 12 | 2 | 1.94E-100 |  |
| Fisuc_0610 | hypothetical protein | 114 | 20 | 23 | 2 | 0 |  |
| Fisuc_0611 | hypothetical protein | 121 | 19 | 23 | 2 | 0 |  |
| Fisuc_0612 | Fis family transcriptional regulator | 89 | 23 | 15 | 1 | 0 |  |
| Fisuc_0613* | serine/threonine protein kinase | 49 | 12 | 20 | 2 | 8.02E-57 |  |
| Fisuc_0614 | hypothetical protein | 49 | 10 | 38 | 3 | 3.65E-06 |  |
| Fisuc_0615 | hypothetical protein | 1837 | 242 | 1326 | 82 | 9.12E-12 |  |
| Fisuc_0616 | NUDIX hydrolase | 43 | 6 | 33 | 5 | 2.20E-09 |  |
| Fisuc_0617 | extracellular solute-binding protein | 463 | 23 | 270 | 24 | 1.85E-13 |  |
| Fisuc_0618 | diguanylate cyclase | 526 | 40 | 310 | 23 | 1.35E-20 |  |
| Fisuc_0619* | yjbN family TIM-barrel protein | 270 | 22 | 185 | 7 | 1.99E-07 |  |
| Fisuc_0621 | biotin synthase | 201 | 24 | 315 | 21 | 4.41E-07 |  |
| Fisuc_0622 | Lanthionine synthetase C family protein | 53 | 4 | 113 | 11 | 3.50E-17 |  |
| Fisuc_0623 | pyridoxal-5'-phosphate-dependent protein subunit beta | 21 | 1 | 97 | 29 | 7.94E-186 |  |
| Fisuc_0624 | short-chain dehydrogenase/reductase SDR | 46 | 6 | 204 | 32 | 2.61E-172 |  |
| Fisuc_0628 | hypothetical protein | 36 | 6 | 24 | 2 | 3.58E-07 |  |
| Fisuc_0629 | hypothetical protein | 22 | 1 | 18 | 1 | 9.23E-05 |  |
| Fisuc_0630 | hypothetical protein | 87 | 8 | 146 | 10 | 3.20E-08 |  |
| Fisuc_0631 | hypothetical protein | 59 | 25 | 114 | 13 | 1.44E-19 |  |
| Fisuc_0632 | imidazole glycerol phosphate synthase, glutamine amidotransferase subunit | 96 | 28 | 367 | 24 | 1.02E-120 |  |
| Fisuc_0634 | hypothetical protein | 97 | 14 | 68 | 10 | 8.64E-07 |  |
| Fisuc_0635 | hypothetical protein | 100 | 19 | 78 | 6 | 1.24E-04 |  |
| Fisuc_0637 | mammalian cell entry domain-containing protein | 174 | 17 | 132 | 10 | 8.02E-06 |  |
| Fisuc_0643 | hypothetical protein | 56 | 2 | 38 | 5 | 3.79E-15 |  |
| Fisuc_0645 | hypothetical protein | 227 | 42 | 76 | 19 | 2.98E-120 |  |
| Fisuc_0646* | flavodoxin | 201 | 21 | 71 | 10 | 3.24E-60 |  |
| Fisuc_0647 | transferase hexapeptide repeat containing protein | 12 | 2 | 8 | 1 | 3.32E-04 |  |
| Fisuc_0650 | plasmid stabilization system | 39 | 6 | 30 | 3 | 4.23E-08 |  |
| Fisuc_0651 | prevent-host-death family protein | 59 | 10 | 46 | 3 | 7.21E-09 |  |
| Fisuc_0653* | diguanylate cyclase and metal dependent phosphohydrolase | 137 | 12 | 40 | 8 | 8.04E-82 |  |
| Fisuc_0654* | citrate synthase I | 377 | 24 | 642 | 8 | 9.76E-06 |  |
| Fisuc_0655 | hypothetical protein | 311 | 4 | 627 | 5 | 1.13E-11 |  |
| Fisuc_0656* | metal dependent phosphohydrolase | 367 | 87 | 150 | 12 | 2.00E-42 |  |
| Fisuc_0657 | hypothetical protein | 2819 | 1325 | 1751 | 206 | 3.90E-03 |  |
| Fisuc_0658 | hypothetical protein | 1883 | 840 | 1201 | 163 | 2.14E-11 |  |
| Fisuc_0659* | dihydrouridine synthase DuS | 22 | 13 | 33 | 2 | 1.05E-05 |  |
| Fisuc_0660* | histone family protein DNA-binding protein | 432 | 43 | 853 | 53 | 2.12E-17 |  |
| Fisuc_0661* | pseudouridine synthase | 69 | 14 | 91 | 5 | 1.30E-03 |  |
| Fisuc_0665 | Lytic transglycosylase catalytic | 48 | 16 | 65 | 4 | 1.60E-03 | YES |
| Fisuc_0668 | glycoside hydrolase family protein | 326 | 16 | 282 | 13 | 8.33E-06 | YES |
| Fisuc_0669* | tRNA delta(2)-isopentenylpyrophosphate transferase | 51 | 8 | 68 | 5 | 1.90E-03 |  |
| Fisuc_0673 | hypothetical protein | 552 | 12 | 370 | 12 | 6.86E-08 |  |
| Fisuc_0674* | hypothetical protein | 54 | 5 | 10 | 1 | 0 |  |
| Fisuc_0675* | isocitrate dehydrogenase | 134 | 18 | 211 | 3 | 1.27E-03 |  |
| Fisuc_0676 | hypothetical protein | 13 | 1 | 29 | 3 | 3.88E-25 |  |
| Fisuc_0677 | hypothetical protein | 9 | 1 | 22 | 1 | 1.38E-29 |  |
| Fisuc_0678 | pectate lyase | 75 | 6 | 25 | 2 | 1.80E-67 | YES |
| Fisuc_0679 | pectinesterase | 60 | 8 | 34 | 2 | 1.49E-14 | YES |
| Fisuc_0682 | hypothetical protein | 113 | 6 | 76 | 1 | 3.73E-16 |  |
| Fisuc_0688 | hypothetical protein | 137 | 21 | 81 | 5 | 5.41E-14 |  |
| Fisuc_0690 | hypothetical protein | 1570 | 105 | 1095 | 38 | 8.53E-08 |  |
| Fisuc_0696* | RNA polymerase sigma-32 subunit RpoH | 830 | 45 | 1681 | 35 | 2.58E-08 |  |
| Fisuc_0700* | valyl-tRNA synthetase | 285 | 43 | 503 | 25 | 1.30E-05 |  |
| Fisuc_0701 | hypothetical protein | 51 | 12 | 40 | 0 | 2.08E-04 |  |
| Fisuc_0702 | hypothetical protein | 70 | 8 | 42 | 2 | 4.69E-12 |  |
| Fisuc_0703 | hypothetical protein | 1 | 1 | 1 | 0 | 3.03E-03 |  |
| Fisuc_0706 | hypothetical protein | 86 | 7 | 70 | 3 | 3.18E-03 |  |
| Fisuc_0708 | beta-lactamase | 228 | 45 | 47 | 4 | 4.13E-256 |  |
| Fisuc_0714 | hypothetical protein | 819 | 65 | 1254 | 62 | 1.51E-03 |  |
| Fisuc_0717 | glycoside hydrolase family protein | 127 | 20 | 261 | 14 | 8.88E-18 | YES |
| Fisuc_0718 | hypothetical protein | 804 | 59 | 1384 | 48 | 3.12E-03 |  |
| Fisuc_0719 | hypothetical protein | 348 | 21 | 568 | 26 | 4.91E-05 |  |
| Fisuc_0720 | hypothetical protein | 355 | 19 | 563 | 22 | 9.37E-04 |  |
| Fisuc_0723 | M6 family metalloprotease domain-containing protein | 102 | 21 | 13 | 1 | 0 |  |
| Fisuc_0726 | aldose 1-epimerase | 59 | 17 | 85 | 7 | 5.22E-05 |  |
| Fisuc_0727 | mannan endo-1,4-beta-mannosidase | 49 | 14 | 7 | 1 | 0 | YES |
| Fisuc_0728 | mannan endo-1,4-beta-mannosidase | 244 | 34 | 153 | 3 | 7.83E-10 | YES |
| Fisuc_0729 | mannan endo-1,4-beta-mannosidase | 72 | 17 | 10 | 2 | 0 | YES |
| Fisuc_0730 | mannan endo-1,4-beta-mannosidase | 101 | 10 | 14 | 2 | 0 | YES |
| Fisuc_0738 | trans-2-enoyl-CoA reductase | 39 | 2 | 81 | 2 | 4.71E-23 |  |
| Fisuc_0739 | amino acid carrier protein | 88 | 11 | 132 | 4 | 3.98E-05 |  |
| Fisuc_0740 | hypothetical protein | 93 | 16 | 34 | 8 | 3.49E-48 |  |
| Fisuc_0741 | hypothetical protein | 1581 | 110 | 990 | 50 | 4.02E-05 |  |
| Fisuc_0742 | hypothetical protein | 104 | 42 | 154 | 13 | 3.16E-05 |  |
| Fisuc_0745* | replicative DNA helicase | 448 | 29 | 383 | 29 | 2.13E-06 |  |
| Fisuc_0749* | 30S ribosomal protein S1 | 1524 | 113 | 2942 | 155 | 4.27E-03 |  |
| Fisuc_0751 | hypothetical protein | 191 | 20 | 147 | 14 | 9.13E-05 |  |
| Fisuc_0754 | endo-1,4-beta-xylanase | 89 | 11 | 32 | 0 | 3.17E-55 | YES |
| Fisuc_0755 | hypothetical protein | 130 | 5 | 84 | 6 | 8.75E-11 |  |
| Fisuc_0756 | hypothetical protein | 163 | 1 | 120 | 5 | 4.14E-04 |  |
| Fisuc_0757 | endo-1,4-beta-xylanase | 26 | 2 | 9 | 1 | 1.45E-90 | YES |
| Fisuc_0758 | thioredoxin | 5733 | 934 | 1313 | 627 | 6.43E-149 |  |
| Fisuc_0759 | CarD family transcriptional regulator | 819 | 152 | 164 | 81 | 1.22E-236 |  |
| Fisuc_0760 | hypothetical protein | 15 | 3 | 2 | 1 | 0 |  |
| Fisuc_0761* | cysteinyl-tRNA synthetase | 286 | 8 | 488 | 24 | 5.20E-06 |  |
| Fisuc_0762 | major facilitator superfamily protein | 53 | 6 | 97 | 5 | 1.97E-09 |  |
| Fisuc_0763 | beta-lactamase | 83 | 3 | 150 | 16 | 7.90E-15 |  |
| Fisuc_0764 | acyltransferase 3 | 36 | 5 | 58 | 1 | 2.93E-08 |  |
| Fisuc_0765 | hypothetical protein | 106 | 2 | 155 | 11 | 1.08E-04 |  |
| Fisuc_0767 | hypothetical protein | 1653 | 107 | 508 | 38 | 4.49E-112 |  |
| Fisuc_0768 | phosphoglyceromutase | 509 | 67 | 1025 | 51 | 5.73E-08 |  |
| Fisuc_0770 | hypothetical protein | 18 | 9 | 31 | 7 | 5.53E-08 |  |
| Fisuc_0771 | hypothetical protein | 192 | 9 | 111 | 21 | 7.09E-15 |  |
| Fisuc_0772 | hypothetical protein | 143 | 14 | 77 | 9 | 3.13E-28 |  |
| Fisuc_0773* | phenylalanyl-tRNA synthetase subunit alpha | 657 | 46 | 1267 | 17 | 1.39E-06 |  |
| Fisuc_0776 | protein tyrosine phosphatase | 490 | 32 | 718 | 20 | 6.00E-06 |  |
| Fisuc_0779 | glucose-1-phosphate thymidylyltransferase | 409 | 34 | 284 | 35 | 2.52E-09 |  |
| Fisuc_0780* | DEAD/DEAH box helicase | 29 | 5 | 41 | 2 | 1.39E-03 |  |
| Fisuc_0786 | cellulase | 61 | 19 | 7 | 1 | 0 | YES |
| Fisuc_0790* | malate dehydrogenase | 55 | 1 | 270 | 4 | 1.18E-191 |  |
| Fisuc_0791 | peptidase M16 domain-containing protein | 97 | 7 | 134 | 5 | 2.98E-03 |  |
| Fisuc_0792* | 50S ribosomal protein L20 | 2152 | 143 | 4295 | 300 | 2.82E-08 |  |
| Fisuc_0793* | 50S ribosomal protein L35 | 3134 | 202 | 6187 | 374 | 3.65E-06 |  |
| Fisuc_0796 | hypothetical protein | 34 | 5 | 29 | 5 | 1.43E-05 |  |
| Fisuc_0797 | hypothetical protein | 82 | 3 | 54 | 3 | 1.97E-08 |  |
| Fisuc_0798 | hypothetical protein | 151 | 10 | 91 | 1 | 2.89E-12 |  |
| Fisuc_0799 | hypothetical protein | 224 | 9 | 138 | 13 | 7.66E-11 |  |
| Fisuc_0801 | hypothetical protein | 51 | 10 | 32 | 5 | 1.32E-08 |  |
| Fisuc_0803 | diguanylate cyclase | 36 | 6 | 31 | 3 | 9.47E-06 |  |
| Fisuc_0804 | hypothetical protein | 24 | 3 | 15 | 1 | 2.52E-20 |  |
| Fisuc_0805 | hypothetical protein | 15 | 2 | 27 | 2 | 2.26E-07 |  |
| Fisuc_0806 | hypothetical protein | 47 | 1 | 37 | 6 | 2.20E-03 |  |
| Fisuc_0808 | lipoprotein | 136 | 7 | 94 | 3 | 6.76E-07 |  |
| Fisuc_0809 | lipoprotein | 68 | 5 | 32 | 3 | 7.14E-26 |  |
| Fisuc_0810 | hypothetical protein | 206 | 50 | 100 | 11 | 1.62E-35 |  |
| Fisuc_0811 | hypothetical protein | 174 | 45 | 71 | 8 | 3.11E-38 |  |
| Fisuc_0812 | hypothetical protein | 120 | 30 | 38 | 6 | 2.17E-76 |  |
| Fisuc_0813 | carbamoyl-phosphate synthase large subunit | 12 | 3 | 16 | 2 | 4.26E-03 |  |
| Fisuc_0815 | hypothetical protein | 184 | 28 | 133 | 2 | 1.95E-05 |  |
| Fisuc_0821 | hypothetical protein | 307 | 83 | 220 | 5 | 2.37E-07 |  |
| Fisuc_0822 | FG-GAP repeat-containing protein | 188 | 40 | 170 | 7 | 2.31E-03 |  |
| Fisuc_0823* | hypothetical protein | 253 | 60 | 74 | 7 | 4.22E-83 | YES |
| Fisuc_0824 | hypothetical protein | 155 | 45 | 49 | 6 | 7.13E-89 |  |
| Fisuc_0826 | hypothetical protein | 4 | 2 | 2 | 0 | 1.63E-09 |  |
| Fisuc_0827 | amine oxidase | 8 | 2 | 2 | 1 | 6.88E-55 |  |
| Fisuc_0828 | GtrA family protein | 29 | 8 | 7 | 2 | 5.37E-195 |  |
| Fisuc_0829 | hypothetical protein | 85 | 17 | 23 | 1 | 1.42E-161 |  |
| Fisuc_0830 | hypothetical protein | 123 | 28 | 31 | 3 | 3.18E-153 |  |
| Fisuc_0831 | hypothetical protein | 153 | 27 | 33 | 3 | 1.69E-216 |  |
| Fisuc_0832 | hypothetical protein | 133 | 26 | 35 | 6 | 6.99E-199 |  |
| Fisuc_0833 | hypothetical protein | 1711 | 101 | 738 | 107 | 2.08E-42 |  |
| Fisuc_0834 | hypothetical protein | 2314 | 121 | 1036 | 135 | 1.17E-40 |  |
| Fisuc_0835 | carbamoyl-phosphate synthase large subunit | 76 | 17 | 132 | 25 | 8.43E-11 |  |
| Fisuc_0837 | hypothetical protein | 590 | 24 | 431 | 48 | 2.14E-05 |  |
| Fisuc_0841 | hypothetical protein | 104 | 10 | 88 | 6 | 7.58E-03 |  |
| Fisuc_0846 | hypothetical protein | 116 | 23 | 55 | 4 | 8.87E-26 |  |
| Fisuc_0847 | hypothetical protein | 36 | 19 | 3 | 0 | 0 |  |
| Fisuc_0848 | hypothetical protein | 16 | 12 | 4 | 1 | 1.02E-137 |  |
| Fisuc_0849 | signal transduction histidine kinase LytS | 38 | 5 | 29 | 1 | 1.82E-03 |  |
| Fisuc_0850* | RNA polymerase sigma-32 subunit RpoH | 2072 | 189 | 5565 | 580 | 4.04E-06 |  |
| Fisuc_0852 | hypothetical protein | 384 | 6 | 572 | 13 | 7.78E-03 |  |
| Fisuc_0855 | polyprenyl synthetase | 202 | 11 | 307 | 17 | 1.95E-06 |  |
| Fisuc_0856 | hypothetical protein | 74 | 4 | 36 | 4 | 6.89E-33 |  |
| Fisuc_0857 | Mg2 transporter protein CorA family protein | 266 | 22 | 544 | 36 | 7.10E-15 |  |
| Fisuc_0859 | alpha amylase | 102 | 23 | 67 | 8 | 1.31E-07 | YES |
| Fisuc_0860 | 4-alpha-glucanotransferase | 227 | 18 | 150 | 5 | 4.02E-07 | YES |
| Fisuc_0861 | hypothetical protein | 90 | 8 | 189 | 13 | 1.86E-21 |  |
| Fisuc_0862 | hypothetical protein | 141 | 3 | 261 | 19 | 1.37E-11 |  |
| Fisuc_0863 | hypothetical protein | 133 | 6 | 184 | 9 | 9.37E-03 |  |
| Fisuc_0864 | hypothetical protein | 82 | 10 | 110 | 7 | 3.31E-03 |  |
| Fisuc_0865* | tRNA (5-methylaminomethyl-2-thiouridylate)-methyltransferase | 124 | 13 | 177 | 18 | 2.80E-04 |  |
| Fisuc_0868 | leucine-rich repeat protein | 164 | 2 | 118 | 11 | 6.53E-05 |  |
| Fisuc_0869 | peptidase S41 | 47 | 2 | 27 | 2 | 1.60E-14 |  |
| Fisuc_0878 | N-acetyltransferase GCN5 | 126 | 23 | 30 | 1 | 1.12E-268 |  |
| Fisuc_0879 | aminoglycoside phosphotransferase | 131 | 14 | 11 | 1 | 0 |  |
| Fisuc_0880 | aminoglycoside phosphotransferase | 56 | 15 | 4 | 1 | 0 |  |
| Fisuc_0883 | hypothetical protein | 64 | 10 | 94 | 13 | 3.51E-04 |  |
| Fisuc_0886 | hypothetical protein | 295 | 45 | 180 | 5 | 1.11E-08 |  |
| Fisuc_0887 | VTC domain-containing protein | 233 | 34 | 148 | 6 | 1.60E-09 |  |
| Fisuc_0888 | hypothetical protein | 256 | 34 | 144 | 4 | 4.98E-15 |  |
| Fisuc_0889 | hypothetical protein | 246 | 26 | 150 | 6 | 2.49E-11 |  |
| Fisuc_0890 | hypothetical protein | 326 | 25 | 208 | 10 | 3.99E-12 |  |
| Fisuc_0891 | cell wall/surface repeat protein | 30 | 4 | 14 | 1 | 3.33E-30 |  |
| Fisuc_0892 | hypothetical protein | 31 | 13 | 3 | 1 | 0 |  |
| Fisuc_0893 | hypothetical protein | 52 | 7 | 20 | 1 | 1.66E-91 |  |
| Fisuc_0894 | hypothetical protein | 40 | 5 | 15 | 1 | 2.67E-99 |  |
| Fisuc_0896 | hypothetical protein | 326 | 24 | 178 | 11 | 7.72E-15 |  |
| Fisuc_0897 | cellulase | 109 | 6 | 16 | 1 | 0 | YES |
| Fisuc_0898 | glutamate synthase (NADPH), homotetrameric | 358 | 62 | 553 | 32 | 6.25E-03 |  |
| Fisuc_0899* | ferredoxin-NADP(+) reductase subunit alpha | 322 | 79 | 595 | 37 | 4.63E-07 |  |
| Fisuc_0901 | hypothetical protein | 81 | 7 | 67 | 4 | 2.49E-03 |  |
| Fisuc_0902 | hypothetical protein | 34 | 13 | 20 | 1 | 2.53E-18 |  |
| Fisuc_0903 | hypothetical protein | 29 | 10 | 18 | 1 | 1.79E-17 |  |
| Fisuc_0904 | biopolymer transport protein ExbD/TolR | 42 | 19 | 23 | 3 | 3.13E-23 |  |
| Fisuc_0905 | MotA/TolQ/ExbB proton channel | 37 | 18 | 23 | 1 | 4.36E-20 |  |
| Fisuc_0906 | MotA/TolQ/ExbB proton channel | 50 | 19 | 31 | 1 | 4.78E-11 |  |
| Fisuc_0907 | hypothetical protein | 49 | 17 | 29 | 3 | 5.80E-16 |  |
| Fisuc_0908 | hypothetical protein | 25 | 13 | 16 | 2 | 1.21E-15 |  |
| Fisuc_0909 | TonB-dependent receptor plug | 23 | 8 | 14 | 1 | 3.75E-13 |  |
| Fisuc_0910 | peptidase C14 caspase catalytic subunit p20 | 20 | 12 | 16 | 2 | 9.03E-03 |  |
| Fisuc_0911 | hypothetical protein | 68 | 22 | 31 | 3 | 2.37E-40 |  |
| Fisuc_0912* | ECF subfamily RNA polymerase sigma-24 subunit | 129 | 34 | 68 | 4 | 5.69E-23 |  |
| Fisuc_0913 | nitroreductase | 75 | 5 | 55 | 6 | 9.98E-05 |  |
| Fisuc_0915 | peptidase T | 31 | 5 | 23 | 2 | 9.40E-09 |  |
| Fisuc_0916 | citrate transporter | 302 | 34 | 22 | 7 | 0 |  |
| Fisuc_0917 | hypothetical protein | 675 | 107 | 55 | 17 | 0 |  |
| Fisuc_0918 | oxidoreductase domain-containing protein | 34 | 6 | 2 | 1 | 0 |  |
| Fisuc_0920 | NUDIX hydrolase | 76 | 3 | 103 | 6 | 3.04E-03 |  |
| Fisuc_0924 | carbohydrate binding family 6 | 7 | 4 | 1 | 0 | 4.84E-126 | YES |
| Fisuc_0926 | hypothetical protein | 76 | 2 | 224 | 29 | 7.87E-61 |  |
| Fisuc_0929 | hypothetical protein | 8 | 2 | 4 | 0 | 2.11E-17 |  |
| Fisuc_0931 | hypothetical protein | 161 | 45 | 112 | 6 | 7.89E-05 |  |
| Fisuc_0932 | hypothetical protein | 94 | 32 | 37 | 6 | 4.18E-63 |  |
| Fisuc_0933* | transcriptional regulator, XRE family | 61 | 16 | 25 | 2 | 1.07E-42 |  |
| Fisuc_0934 | hypothetical protein | 56 | 4 | 9 | 1 | 0 |  |
| Fisuc_0935 | hypothetical protein | 21 | 5 | 5 | 0 | 7.58E-120 |  |
| Fisuc_0936 | hypothetical protein | 28 | 2 | 6 | 2 | 2.19E-134 |  |
| Fisuc_0937 | hypothetical protein | 32 | 5 | 7 | 1 | 8.35E-201 |  |
| Fisuc_0940 | DNA-binding domain-containing protein | 16 | 1 | 5 | 1 | 1.25E-59 |  |
| Fisuc_0941 | hypothetical protein | 6 | 1 | 2 | 1 | 1.42E-31 |  |
| Fisuc_0942* | transcriptional regulator | 2 | 0 | 0 | 1 | 1.08E-52 |  |
| Fisuc_0944* | Na+/H+ antiporter NhaC-like protein | 37 | 2 | 57 | 3 | 3.45E-07 |  |
| Fisuc_0945 | hypothetical protein | 93 | 0 | 155 | 31 | 1.07E-09 |  |
| Fisuc_0946 | hypothetical protein | 46 | 6 | 24 | 3 | 4.15E-37 |  |
| Fisuc_0947* | translation elongation factor Ts | 1997 | 85 | 4409 | 128 | 2.88E-03 |  |
| Fisuc_0948* | 30S ribosomal protein S2 | 1895 | 91 | 4354 | 165 | 1.86E-06 |  |
| Fisuc_0953 | radical SAM protein | 255 | 9 | 195 | 6 | 7.39E-06 |  |
| Fisuc_0954 | hypothetical protein | 11 | 2 | 7 | 1 | 9.21E-16 |  |
| Fisuc_0955 | hypothetical protein | 570 | 71 | 274 | 6 | 2.25E-26 |  |
| Fisuc_0956 | twitching motility protein PilT | 368 | 45 | 188 | 12 | 2.98E-20 |  |
| Fisuc_0958 | hypothetical protein | 42 | 6 | 21 | 5 | 4.90E-17 |  |
| Fisuc_0959 | aminoglycoside phosphotransferase | 13 | 4 | 1 | 0 | 0 |  |
| Fisuc_0960 | N-acetyltransferase GCN5 | 20 | 6 | 2 | 1 | 0 |  |
| Fisuc_0965 | hypothetical protein | 203 | 21 | 258 | 20 | 8.50E-03 |  |
| Fisuc_0969 | glycerol-3-phosphate cytidylyltransferase | 157 | 25 | 131 | 6 | 3.57E-03 |  |
| Fisuc_0972* | transcriptional regulator | 10 | 3 | 7 | 1 | 2.51E-09 |  |
| Fisuc_0973 | Multimeric flavodoxin WrbA-like protein | 145 | 21 | 300 | 2 | 4.44E-17 |  |
| Fisuc_0974* | 30S ribosomal protein S9 | 1714 | 102 | 3754 | 61 | 1.29E-10 |  |
| Fisuc_0975* | 50S ribosomal protein L13 | 2103 | 100 | 4984 | 141 | 4.78E-09 |  |
| Fisuc_0977 | signal peptide peptidase SppA, 36K type | 34 | 6 | 25 | 2 | 3.66E-06 |  |
| Fisuc_0983 | glycosyltransferase family protein | 72 | 19 | 99 | 7 | 6.15E-04 | YES |
| Fisuc_0988 | hypothetical protein | 104 | 23 | 215 | 22 | 1.27E-16 |  |
| Fisuc_0989 | hypothetical protein | 142 | 17 | 243 | 18 | 1.15E-08 |  |
| Fisuc_0990 | NAD-dependent epimerase/dehydratase | 170 | 8 | 244 | 16 | 6.95E-04 |  |
| Fisuc_0991 | metallophosphoesterase | 109 | 9 | 172 | 13 | 3.49E-05 |  |
| Fisuc_0992 | phosphonopyruvate decarboxylase | 122 | 19 | 208 | 18 | 1.38E-08 |  |
| Fisuc_0993 | phosphoenolpyruvate phosphomutase | 202 | 16 | 318 | 27 | 2.07E-05 |  |
| Fisuc_0995 | NAD-dependent epimerase/dehydratase | 154 | 17 | 202 | 12 | 8.42E-03 |  |
| Fisuc_0997 | nucleotide sugar dehydrogenase | 173 | 8 | 219 | 3 | 7.39E-03 |  |
| Fisuc_0998 | spore coat protein CotH | 19 | 2 | 13 | 1 | 3.05E-07 |  |
| Fisuc_1000 | Lytic transglycosylase catalytic | 254 | 13 | 234 | 4 | 1.21E-04 | YES |
| Fisuc_1001 | phosphoenolpyruvate-protein phosphotransferase | 344 | 28 | 304 | 7 | 1.54E-05 |  |
| Fisuc_1002 | phosphotransferase system, phosphocarrier protein HPr | 938 | 61 | 668 | 44 | 1.02E-05 |  |
| Fisuc_1003 | mammalian cell entry domain-containing protein | 875 | 61 | 564 | 42 | 2.03E-15 |  |
| Fisuc_1004* | DNA-directed RNA polymerase subunit beta' | 69 | 6 | 46 | 2 | 9.58E-09 |  |
| Fisuc_1008 | von Willebrand factor type A | 177 | 12 | 347 | 55 | 3.35E-17 |  |
| Fisuc_1010 | von Willebrand factor type A | 184 | 22 | 329 | 33 | 3.46E-12 |  |
| Fisuc_1011 | branched-chain amino acid transport | 61 | 4 | 96 | 8 | 8.86E-05 |  |
| Fisuc_1012 | AzlC family protein | 95 | 3 | 236 | 6 | 3.96E-31 |  |
| Fisuc_1013 | hypothetical protein | 2815 | 51 | 1667 | 113 | 1.19E-10 |  |
| Fisuc_1014 | aminodeoxychorismate lyase | 134 | 16 | 108 | 3 | 5.73E-03 |  |
| Fisuc_1015 | hypothetical protein | 113 | 11 | 63 | 1 | 9.65E-20 |  |
| Fisuc_1016 | Tfp pilus assembly protein pilus retraction ATPase PilT-like protein | 123 | 4 | 61 | 1 | 6.63E-24 |  |
| Fisuc_1017 | Tfp pilus assembly protein pilus retraction ATPase PilT-like protein | 98 | 10 | 64 | 3 | 1.61E-06 |  |
| Fisuc_1018 | ABC transporter ATPase | 144 | 7 | 74 | 8 | 5.43E-22 |  |
| Fisuc_1019 | ABC transporter | 200 | 14 | 100 | 15 | 4.75E-18 |  |
| Fisuc_1020 | hypothetical protein | 258 | 12 | 158 | 21 | 9.90E-13 |  |
| Fisuc_1021 | hypothetical protein | 946 | 391 | 3968 | 543 | 1.36E-71 |  |
| Fisuc_1023 | hypothetical protein | 9 | 3 | 7 | 1 | 1.51E-07 |  |
| Fisuc_1025 | hypothetical protein | 16 | 1 | 43 | 3 | 1.65E-38 |  |
| Fisuc_1026 | hypothetical protein | 12 | 0 | 29 | 2 | 6.32E-32 |  |
| Fisuc_1027 | hypothetical protein | 7 | 2 | 19 | 3 | 1.23E-41 |  |
| Fisuc_1028 | hypothetical protein | 45 | 14 | 32 | 1 | 1.52E-06 |  |
| Fisuc_1029 | hypothetical protein | 40 | 12 | 31 | 1 | 7.11E-06 |  |
| Fisuc_1030 | GSCFA domain-containing protein | 42 | 4 | 24 | 4 | 2.38E-20 |  |
| Fisuc_1031 | hypothetical protein | 30 | 3 | 52 | 2 | 3.44E-11 |  |
| Fisuc_1032 | hypothetical protein | 20 | 3 | 46 | 4 | 1.19E-22 |  |
| Fisuc_1034 | diguanylate cyclase | 56 | 8 | 35 | 3 | 3.68E-10 |  |
| Fisuc_1035 | binding-protein-dependent transport system inner membrane protein | 3 | 1 | 9 | 2 | 9.90E-29 |  |
| Fisuc_1036 | ABC transporter | 6 | 1 | 20 | 6 | 8.94E-55 |  |
| Fisuc_1037 | NLPA lipoprotein | 13 | 1 | 37 | 9 | 1.37E-45 |  |
| Fisuc_1039* | alanyl-tRNA synthetase | 168 | 9 | 370 | 16 | 7.51E-14 |  |
| Fisuc_1041 | hypothetical protein | 152 | 11 | 358 | 33 | 4.12E-24 |  |
| Fisuc_1042 | hypothetical protein | 137 | 12 | 342 | 40 | 2.07E-31 |  |
| Fisuc_1043 | licheninase | 14 | 1 | 7 | 1 | 2.42E-32 | YES |
| Fisuc_1044* | formate acetyltransferase | 246 | 23 | 1159 | 85 | 2.09E-77 |  |
| Fisuc_1045 | hypothetical protein | 81 | 3 | 107 | 7 | 4.90E-03 |  |
| Fisuc_1048* | helicase domain-containing protein | 71 | 7 | 53 | 3 | 2.23E-06 |  |
| Fisuc_1050 | type II secretion system F domain-containing protein | 252 | 14 | 162 | 9 | 8.22E-10 |  |
| Fisuc_1051 | twitching motility protein | 286 | 16 | 186 | 9 | 1.53E-09 |  |
| Fisuc_1053* | tRNA synthetase class II (D K and N) | 81 | 15 | 66 | 3 | 1.20E-03 |  |
| Fisuc_1054 | hypothetical protein | 37 | 8 | 14 | 1 | 1.87E-74 |  |
| Fisuc_1055 | SufS subfamily cysteine desulfurase | 79 | 3 | 186 | 20 | 9.41E-24 |  |
| Fisuc_1056 | UBA/THIF-type NAD/FAD binding protein | 40 | 6 | 72 | 3 | 1.41E-11 |  |
| Fisuc_1058 | hypothetical protein | 25 | 4 | 13 | 2 | 3.18E-23 |  |
| Fisuc_1059 | hypothetical protein | 56 | 8 | 36 | 5 | 7.28E-15 |  |
| Fisuc_1062 | hypothetical protein | 56 | 5 | 46 | 6 | 1.71E-03 |  |
| Fisuc_1063 | hypothetical protein | 93 | 2 | 57 | 6 | 4.24E-11 |  |
| Fisuc_1064 | hypothetical protein | 95 | 14 | 58 | 6 | 5.78E-11 |  |
| Fisuc_1065 | hypothetical protein | 113 | 16 | 75 | 9 | 4.30E-15 |  |
| Fisuc_1066 | hypothetical protein | 120 | 8 | 77 | 5 | 4.00E-09 |  |
| Fisuc_1067 | ATPase | 18 | 1 | 28 | 1 | 4.29E-06 |  |
| Fisuc_1068 | filamentation induced by cAMP protein fic | 31 | 1 | 45 | 11 | 8.52E-03 |  |
| Fisuc_1069 | phospholipase D/Transphosphatidylase | 50 | 2 | 74 | 9 | 1.11E-04 |  |
| Fisuc_1071 | O-acetylhomoserine/O-acetylserine sulfhydrylase | 376 | 106 | 1792 | 127 | 1.38E-119 |  |
| Fisuc_1072 | cysteine synthase A | 232 | 115 | 902 | 84 | 1.23E-116 |  |
| Fisuc_1073* | tRNA (5-methylaminomethyl-2-thiouridylate)-methyltransferase | 70 | 16 | 105 | 5 | 1.96E-04 |  |
| Fisuc_1074 | hypothetical protein | 51 | 13 | 39 | 4 | 5.30E-03 |  |
| Fisuc_1075 | nitrogenase iron protein | 56 | 4 | 382 | 70 | 0 |  |
| Fisuc_1076 | radical SAM protein | 40 | 4 | 313 | 85 | 0 |  |
| Fisuc_1077* | nitrogenase | 43 | 3 | 238 | 58 | 0 |  |
| Fisuc_1078* | oxidoreductase/nitrogenase component 1 | 32 | 6 | 159 | 28 | 1.12E-235 |  |
| Fisuc_1081 | methyltransferase | 23 | 4 | 34 | 3 | 3.57E-03 |  |
| Fisuc_1082 | binding-protein-dependent transport system inner membrane protein | 15 | 3 | 33 | 7 | 7.05E-23 |  |
| Fisuc_1083 | ABC transporter | 13 | 3 | 52 | 17 | 5.47E-116 |  |
| Fisuc_1084 | glycine betaine ABC transporter substrate-binding protein | 18 | 6 | 66 | 23 | 1.27E-91 |  |
| Fisuc_1085 | hypothetical protein | 16 | 3 | 58 | 22 | 3.20E-88 |  |
| Fisuc_1086* | nitrogenase | 25 | 4 | 106 | 13 | 2.24E-140 |  |
| Fisuc_1087* | nitrogenase | 23 | 6 | 124 | 26 | 8.39E-276 |  |
| Fisuc_1088 | nitrogenase iron protein | 23 | 3 | 177 | 43 | 0 |  |
| Fisuc_1089 | dinitrogenase iron-molybdenum cofactor biosynthesis protein | 42 | 1 | 346 | 61 | 0 |  |
| Fisuc_1090 | N-acetyltransferase GCN5 | 40 | 2 | 267 | 57 | 0 |  |
| Fisuc_1091 | pyridoxal-5'-phosphate-dependent protein subunit beta | 27 | 1 | 138 | 36 | 4.35E-228 |  |
| Fisuc_1092 | hypothetical protein | 0 | 0 | 5 | 3 | 9.43E-248 |  |
| Fisuc_1093 | nitrate/sulfonate/bicarbonate ABC transporter periplasmic protein | 0 | 0 | 3 | 1 | 2.04E-193 |  |
| Fisuc_1094 | nitrate/sulfonate/bicarbonate ABC transporter periplasmic protein | 14 | 2 | 101 | 36 | 0 |  |
| Fisuc_1095 | pyridoxal-5'-phosphate-dependent protein subunit beta | 17 | 2 | 93 | 28 | 1.23E-257 |  |
| Fisuc_1096 | binding-protein-dependent transport system inner membrane protein | 3 | 1 | 10 | 4 | 1.27E-82 |  |
| Fisuc_1097 | ABC transporter | 3 | 2 | 7 | 2 | 2.50E-25 |  |
| Fisuc_1098 | class I and II aminotransferase | 77 | 11 | 169 | 19 | 1.17E-19 |  |
| Fisuc_1100 | hypothetical protein | 40 | 4 | 153 | 25 | 9.69E-104 |  |
| Fisuc_1101 | hypothetical protein | 86 | 6 | 128 | 28 | 4.12E-04 |  |
| Fisuc_1103* | ribosomal L11 methyltransferase | 371 | 18 | 319 | 18 | 1.29E-03 |  |
| Fisuc_1105 | hypothetical protein | 23 | 3 | 13 | 1 | 2.53E-09 |  |
| Fisuc_1106* | transcriptional regulator | 26 | 2 | 16 | 1 | 3.84E-16 |  |
| Fisuc_1108 | phospholipid/glycerol acyltransferase | 168 | 6 | 106 | 8 | 3.20E-10 |  |
| Fisuc_1109* | VacB and RNase II family 3'-5' exoribonuclease | 177 | 12 | 102 | 3 | 5.16E-17 |  |
| Fisuc_1110 | hypothetical protein | 112 | 5 | 81 | 5 | 7.92E-06 |  |
| Fisuc_1111 | hypothetical protein | 132 | 11 | 418 | 91 | 1.33E-61 |  |
| Fisuc_1112 | phosphoglycerate mutase | 103 | 8 | 59 | 2 | 1.41E-14 |  |
| Fisuc_1114 | hypothetical protein | 5 | 1 | 0 | 1 | 4.27E-251 |  |
| Fisuc_1115 | hypothetical protein | 463 | 57 | 108 | 7 | 3.26E-174 |  |
| Fisuc_1116 | hypothetical protein | 197 | 22 | 69 | 9 | 2.94E-60 |  |
| Fisuc_1117 | hypothetical protein | 149 | 16 | 57 | 6 | 3.17E-51 |  |
| Fisuc_1118* | hypothetical protein | 113 | 18 | 88 | 3 | 1.78E-03 |  |
| Fisuc_1120 | glutamine synthetase | 86 | 8 | 143 | 31 | 8.22E-10 |  |
| Fisuc_1121 | Fis family sigma-54 specific transcriptional regulator | 29 | 5 | 48 | 3 | 4.95E-08 |  |
| Fisuc_1122 | hypothetical protein | 18 | 1 | 26 | 1 | 3.19E-03 |  |
| Fisuc_1126 | O-acetylhomoserine/O-acetylserine sulfhydrylase | 105 | 17 | 329 | 10 | 2.82E-63 |  |
| Fisuc_1129 | hypothetical protein | 59 | 5 | 99 | 7 | 5.90E-06 |  |
| Fisuc_1130 | hypothetical protein | 102 | 9 | 190 | 7 | 3.79E-12 |  |
| Fisuc_1131 | hypothetical protein | 107 | 16 | 187 | 20 | 2.27E-11 |  |
| Fisuc_1132 | hypothetical protein | 232 | 16 | 400 | 74 | 2.57E-11 |  |
| Fisuc_1133 | YidE/YbjL duplication | 1411 | 357 | 4169 | 949 | 9.18E-08 |  |
| Fisuc_1134 | hypothetical protein | 8 | 3 | 4 | 1 | 1.04E-13 |  |
| Fisuc_1135* | transcriptional regulator, MerR family | 7 | 3 | 3 | 1 | 7.66E-16 |  |
| Fisuc_1137 | patatin | 54 | 7 | 13 | 1 | 3.82E-249 |  |
| Fisuc_1138 | hypothetical protein | 61 | 13 | 32 | 2 | 2.37E-27 |  |
| Fisuc_1139 | hypothetical protein | 190 | 15 | 108 | 15 | 8.86E-19 |  |
| Fisuc_1140 | methyltransferase | 97 | 5 | 61 | 1 | 6.67E-11 |  |
| Fisuc_1141 | hypothetical protein | 298 | 95 | 171 | 13 | 3.00E-14 |  |
| Fisuc_1142 | hypothetical protein | 119 | 13 | 40 | 4 | 5.64E-68 |  |
| Fisuc_1143 | phosphoribosyl-ATP diphosphatase | 418 | 14 | 605 | 23 | 5.77E-04 |  |
| Fisuc_1150 | dihydroorotase | 73 | 2 | 102 | 10 | 1.11E-03 |  |
| Fisuc_1151 | OmpA/MotB domain-containing protein | 794 | 29 | 216 | 22 | 1.65E-149 |  |
| Fisuc_1152 | hypothetical protein | 40 | 5 | 17 | 1 | 5.47E-70 |  |
| Fisuc_1153* | DnaB helicase domain-containing protein | 16 | 1 | 9 | 1 | 2.65E-21 |  |
| Fisuc_1154 | hypothetical protein | 3 | 1 | 4 | 1 | 5.19E-03 |  |
| Fisuc_1156 | hypothetical protein | 15 | 2 | 13 | 3 | 2.52E-04 |  |
| Fisuc_1158 | metal-dependent phosphohydrolase HD sub domain-containing protein | 62 | 6 | 49 | 5 | 8.67E-05 |  |
| Fisuc_1159* | leucyl-tRNA synthetase | 161 | 22 | 259 | 8 | 1.00E-04 |  |
| Fisuc_1160 | hypothetical protein | 7 | 2 | 17 | 6 | 1.26E-32 |  |
| Fisuc_1161 | hypothetical protein | 7 | 2 | 14 | 1 | 2.44E-13 |  |
| Fisuc_1163 | hypothetical protein | 297 | 38 | 228 | 10 | 9.57E-04 |  |
| Fisuc_1164 | spermine synthase | 32 | 8 | 8 | 1 | 7.66E-175 |  |
| Fisuc_1166 | hypothetical protein | 136 | 11 | 119 | 7 | 1.23E-05 |  |
| Fisuc_1168 | hypothetical protein | 17 | 2 | 14 | 2 | 1.19E-04 |  |
| Fisuc_1169* | ECF subfamily RNA polymerase sigma-24 subunit | 335 | 111 | 126 | 18 | 2.06E-49 |  |
| Fisuc_1170 | hypothetical protein | 272 | 81 | 95 | 18 | 2.45E-83 |  |
| Fisuc_1171 | hypothetical protein | 399 | 98 | 180 | 38 | 3.08E-32 |  |
| Fisuc_1172 | hypothetical protein | 20 | 6 | 15 | 3 | 1.86E-03 |  |
| Fisuc_1174 | diguanylate cyclase | 119 | 38 | 99 | 13 | 1.30E-03 |  |
| Fisuc_1175 | hypothetical protein | 125 | 15 | 80 | 10 | 1.80E-11 |  |
| Fisuc_1176 | hypothetical protein | 28 | 5 | 21 | 1 | 2.85E-05 |  |
| Fisuc_1177* | SMC domain-containing protein | 89 | 16 | 59 | 4 | 4.18E-09 |  |
| Fisuc_1178* | nuclease SbcCD subunit D | 48 | 13 | 68 | 5 | 1.06E-04 |  |
| Fisuc_1179 | hypothetical protein | 59 | 4 | 40 | 3 | 6.18E-11 |  |
| Fisuc_1180 | histidine kinase | 97 | 7 | 55 | 2 | 4.21E-18 |  |
| Fisuc_1181* | Exodeoxyribonuclease V subunit RecC | 59 | 1 | 87 | 11 | 2.73E-06 |  |
| Fisuc_1183* | exodeoxyribonuclease V | 34 | 3 | 26 | 2 | 1.14E-05 |  |
| Fisuc_1185 | hypothetical protein | 42 | 3 | 35 | 2 | 8.60E-04 |  |
| Fisuc_1186* | transcriptional regulator | 94 | 10 | 40 | 2 | 2.12E-38 |  |
| Fisuc_1187 | DNA-damage-inducible protein D | 7 | 1 | 1 | 0 | 1.67E-270 |  |
| Fisuc_1189 | cobalamin synthesis protein P47K | 5 | 1 | 18 | 1 | 7.98E-66 |  |
| Fisuc_1191 | hypothetical protein | 95 | 16 | 86 | 19 | 4.01E-03 |  |
| Fisuc_1193 | ABC transporter | 19 | 4 | 31 | 2 | 3.98E-05 |  |
| Fisuc_1194 | ABC transporter | 9 | 2 | 18 | 3 | 1.29E-11 |  |
| Fisuc_1195 | hypothetical protein | 11 | 3 | 21 | 4 | 4.71E-10 |  |
| Fisuc_1196 | hypothetical protein | 7 | 1 | 16 | 2 | 2.49E-17 |  |
| Fisuc_1197 | hypothetical protein | 9 | 2 | 13 | 3 | 1.74E-03 |  |
| Fisuc_1198 | hypothetical protein | 1 | 0 | 2 | 1 | 3.04E-07 |  |
| Fisuc_1199 | hypothetical protein | 170 | 5 | 371 | 9 | 3.39E-24 |  |
| Fisuc_1200* | small GTP-binding protein | 65 | 8 | 88 | 8 | 3.30E-03 |  |
| Fisuc_1201 | RelA/SpoT domain-containing protein | 89 | 21 | 67 | 8 | 1.16E-04 |  |
| Fisuc_1202* | metal dependent phosphohydrolase | 166 | 6 | 88 | 10 | 5.73E-20 |  |
| Fisuc_1203 | hypothetical protein | 221 | 2 | 110 | 11 | 2.16E-22 |  |
| Fisuc_1204 | glucose-6-phosphate isomerase | 137 | 21 | 229 | 11 | 8.75E-10 |  |
| Fisuc_1205 | diguanylate cyclase/phosphodiesterase | 179 | 6 | 85 | 15 | 4.16E-26 |  |
| Fisuc_1206 | hypothetical protein | 20 | 1 | 32 | 5 | 7.79E-09 |  |
| Fisuc_1207 | phosphoglycerate mutase | 41 | 10 | 62 | 2 | 3.08E-05 |  |
| Fisuc_1208 | hypothetical protein | 28 | 3 | 40 | 4 | 1.06E-03 |  |
| Fisuc_1209 | ATPase AAA | 323 | 18 | 785 | 92 | 1.70E-20 |  |
| Fisuc_1212 | DNA-damage-inducible protein D | 6 | 2 | 1 | 1 | 9.63E-63 |  |
| Fisuc_1213 | hypothetical protein | 17 | 2 | 13 | 1 | 3.38E-09 |  |
| Fisuc_1216 | hypothetical protein | 26 | 2 | 38 | 3 | 2.08E-04 |  |
| Fisuc_1219 | glycoside hydrolase family protein | 604 | 40 | 369 | 13 | 9.83E-19 | YES |
| Fisuc_1220 | hypothetical protein | 29 | 8 | 20 | 2 | 2.28E-08 |  |
| Fisuc_1221 | hypothetical protein | 113 | 22 | 53 | 3 | 1.39E-28 |  |
| Fisuc_1222 | hypothetical protein | 97 | 22 | 29 | 4 | 2.46E-135 |  |
| Fisuc_1223 | hypothetical protein | 8203 | 869 | 1818 | 97 | 2.21E-34 |  |
| Fisuc_1224 | cellulase | 551 | 133 | 107 | 11 | 7.06E-300 | YES |
| Fisuc_1225 | hypothetical protein | 31 | 3 | 19 | 3 | 5.59E-12 |  |
| Fisuc_1226 | ABC transporter | 634 | 56 | 569 | 32 | 4.42E-03 |  |
| Fisuc_1227 | oligopeptide/dipeptide ABC transporter ATPase | 628 | 88 | 549 | 27 | 1.12E-05 |  |
| Fisuc_1229 | binding-protein-dependent transport system inner membrane protein | 582 | 88 | 465 | 32 | 2.66E-05 |  |
| Fisuc_1230 | family 5 extracellular solute-binding protein | 818 | 90 | 665 | 29 | 1.24E-03 |  |
| Fisuc_1231* | excinuclease ABC subunit C | 66 | 6 | 48 | 1 | 4.64E-04 |  |
| Fisuc_1234 | beta-lactamase | 178 | 12 | 70 | 11 | 1.76E-43 |  |
| Fisuc_1235 | TraR/DksA family transcriptional regulator | 1108 | 44 | 352 | 8 | 3.06E-79 |  |
| Fisuc_1236 | mammalian cell entry domain-containing protein | 183 | 57 | 131 | 9 | 1.32E-05 |  |
| Fisuc_1237 | HPr kinase | 242 | 42 | 96 | 10 | 1.57E-35 |  |
| Fisuc_1238* | sigma 54 modulation protein/ribosomal protein S30EA | 2868 | 419 | 560 | 42 | 6.62E-266 |  |
| Fisuc_1239* | RNA polymerase sigma 54 subunit RpoN | 64 | 9 | 101 | 8 | 3.53E-06 |  |
| Fisuc_1240 | ABC transporter | 70 | 9 | 122 | 12 | 4.30E-13 |  |
| Fisuc_1241 | hypothetical protein | 33 | 4 | 55 | 6 | 2.76E-10 |  |
| Fisuc_1242 | hypothetical protein | 27 | 4 | 59 | 4 | 2.58E-23 |  |
| Fisuc_1243* | pyruvate/ketoisovalerate oxidoreductase | 205 | 14 | 300 | 11 | 2.85E-03 |  |
| Fisuc_1245 | hypothetical protein | 21 | 5 | 36 | 4 | 1.58E-07 |  |
| Fisuc_1249 | hypothetical protein | 172 | 4 | 25 | 2 | 0 |  |
| Fisuc_1250 | hypothetical protein | 222 | 47 | 48 | 12 | 1.71E-221 |  |
| Fisuc_1252 | peptidoglycan glycosyltransferase | 65 | 11 | 89 | 8 | 4.56E-04 | YES |
| Fisuc_1255 | hypothetical protein | 82 | 8 | 44 | 4 | 5.22E-17 |  |
| Fisuc_1256* | excinuclease ABC C subunit domain protein | 76 | 6 | 45 | 4 | 6.83E-24 |  |
| Fisuc_1259 | histidine kinase | 116 | 23 | 64 | 7 | 1.21E-18 |  |
| Fisuc_1265* | Crp family transcriptional regulator | 6 | 1 | 8 | 1 | 6.37E-03 |  |
| Fisuc_1268* | 30S ribosomal protein S7 | 2243 | 381 | 5072 | 252 | 2.77E-05 |  |
| Fisuc_1269* | 30S ribosomal protein S12 | 1774 | 412 | 4499 | 337 | 9.41E-16 |  |
| Fisuc_1272* | 50S ribosomal protein L7/L12 | 6060 | 201 | 12054 | 729 | 3.46E-03 |  |
| Fisuc_1273* | 50S ribosomal protein L10 | 4955 | 117 | 10741 | 380 | 5.20E-04 |  |
| Fisuc_1275* | 50S ribosomal protein L11 | 4199 | 299 | 9003 | 417 | 5.97E-03 |  |
| Fisuc_1276* | NusG antitermination factor | 4101 | 479 | 8170 | 205 | 4.17E-03 |  |
| Fisuc_1277 | Preprotein translocase subunit SecE | 5666 | 966 | 11164 | 608 | 1.37E-03 |  |
| Fisuc_1278* | 50S ribosomal protein L33 | 6024 | 1007 | 11298 | 330 | 4.31E-05 |  |
| Fisuc_1280 | thioesterase superfamily protein | 46 | 4 | 135 | 6 | 3.40E-52 |  |
| Fisuc_1281* | ATPase AAA | 76 | 4 | 144 | 4 | 2.29E-12 |  |
| Fisuc_1282 | hypothetical protein | 25 | 2 | 35 | 1 | 2.15E-04 |  |
| Fisuc_1283 | hypothetical protein | 5719 | 452 | 498 | 51 | 0 |  |
| Fisuc_1284 | hypothetical protein | 5373 | 805 | 219 | 19 | 0 |  |
| Fisuc_1285 | hypothetical protein | 242 | 44 | 20 | 1 | 0 |  |
| Fisuc_1287 | hypothetical protein | 119 | 17 | 110 | 10 | 6.48E-03 |  |
| Fisuc_1288 | hypothetical protein | 79 | 4 | 66 | 2 | 5.82E-03 |  |
| Fisuc_1290 | hypothetical protein | 95 | 13 | 88 | 5 | 5.40E-03 |  |
| Fisuc_1294 | hypothetical protein | 584 | 48 | 1076 | 101 | 3.72E-13 |  |
| Fisuc_1298* | adenine-specific DNA-methyltransferase | 70 | 3 | 54 | 1 | 3.94E-03 |  |
| Fisuc_1305 | hypothetical protein | 31 | 2 | 28 | 2 | 5.86E-04 |  |
| Fisuc_1306* | helicase domain-containing protein | 24 | 3 | 17 | 2 | 3.39E-06 |  |
| Fisuc_1308 | G-D-S-L family lipolytic protein | 46 | 4 | 80 | 2 | 6.79E-13 |  |
| Fisuc_1309 | hypothetical protein | 91 | 3 | 74 | 4 | 1.62E-03 |  |
| Fisuc_1312 | hypothetical protein | 60 | 4 | 43 | 1 | 4.00E-11 |  |
| Fisuc_1313 | hypothetical protein | 182 | 15 | 22 | 5 | 0 |  |
| Fisuc_1314 | hypothetical protein | 57 | 18 | 21 | 3 | 4.62E-78 |  |
| Fisuc_1315 | ABC transporter ATPase | 61 | 8 | 47 | 2 | 5.40E-04 |  |
| Fisuc_1316 | PEGA domain-containing protein | 2443 | 145 | 1920 | 125 | 1.39E-05 |  |
| Fisuc_1319 | hypothetical protein | 589 | 151 | 425 | 31 | 6.96E-04 |  |
| Fisuc_1320* | two component transcriptional regulator, winged helix family | 1022 | 67 | 825 | 14 | 1.67E-07 |  |
| Fisuc_1321 | integral membrane sensor signal transduction histidine kinase | 307 | 45 | 243 | 11 | 4.45E-06 |  |
| Fisuc_1322 | diphosphate--fructose-6-phosphate 1-phosphotransferase | 462 | 31 | 359 | 8 | 4.41E-07 |  |
| Fisuc_1323 | hypothetical protein | 360 | 47 | 266 | 22 | 1.55E-04 |  |
| Fisuc_1326 | fibro-slime family protein | 222 | 50 | 148 | 5 | 2.50E-14 |  |
| Fisuc_1327 | fibro-slime family protein | 377 | 91 | 8 | 1 | 0 |  |
| Fisuc_1329 | hypothetical protein | 31 | 2 | 23 | 2 | 2.89E-10 |  |
| Fisuc_1331 | peptidase M23 | 172 | 11 | 130 | 1 | 1.39E-03 |  |
| Fisuc_1332 | hypothetical protein | 172 | 12 | 121 | 10 | 1.78E-07 |  |
| Fisuc_1334* | DNA polymerase III subunit beta | 1041 | 17 | 953 | 16 | 1.37E-03 |  |
| Fisuc_1335 | PP-loop domain-containing protein | 69 | 4 | 36 | 4 | 1.08E-27 |  |
| Fisuc_1336 | hypothetical protein | 37 | 2 | 20 | 1 | 1.52E-33 |  |
| Fisuc_1337 | hypothetical protein | 12 | 3 | 3 | 1 | 3.17E-62 |  |
| Fisuc_1338 | hypothetical protein | 16 | 3 | 6 | 3 | 1.45E-44 |  |
| Fisuc_1340 | hypothetical protein | 21 | 3 | 15 | 3 | 1.55E-05 |  |
| Fisuc_1343 | hypothetical protein | 87 | 7 | 127 | 11 | 1.03E-05 |  |
| Fisuc_1344 | hypothetical protein | 76 | 10 | 111 | 10 | 2.71E-05 |  |
| Fisuc_1348 | Virulence protein-like protein | 25 | 2 | 82 | 19 | 1.49E-70 |  |
| Fisuc_1350* | transcriptional regulator, LysR family | 25 | 2 | 22 | 1 | 2.24E-04 |  |
| Fisuc_1351 | 5-methyltetrahydropteroyltriglutamate/homocysteine S-methyltransferase | 1551 | 102 | 909 | 203 | 2.32E-05 |  |
| Fisuc_1352 | cytosine/purines uracil thiamine allantoin permease | 55 | 16 | 170 | 29 | 2.83E-61 |  |
| Fisuc_1353 | phosphomethylpyrimidine kinase type-1 | 109 | 11 | 157 | 15 | 5.68E-03 |  |
| Fisuc_1354 | radical SAM protein | 134 | 8 | 226 | 14 | 1.10E-06 |  |
| Fisuc_1355 | hypothetical protein | 31 | 5 | 100 | 15 | 1.61E-65 |  |
| Fisuc_1357 | Appr-1-p processing protein | 43 | 3 | 31 | 0 | 4.99E-07 |  |
| Fisuc_1358 | hypothetical protein | 47 | 4 | 28 | 1 | 1.34E-16 |  |
| Fisuc_1359 | lipoprotein | 38 | 1 | 23 | 3 | 4.82E-19 |  |
| Fisuc_1360 | hypothetical protein | 107 | 15 | 56 | 6 | 8.34E-21 |  |
| Fisuc_1361 | hypothetical protein | 271 | 18 | 121 | 10 | 2.34E-33 |  |
| Fisuc_1362 | lipoprotein | 46 | 6 | 33 | 3 | 1.50E-10 |  |
| Fisuc_1365 | hypothetical protein | 4 | 0 | 16 | 3 | 1.52E-96 |  |
| Fisuc_1366 | hypothetical protein | 173 | 28 | 60 | 8 | 8.31E-62 |  |
| Fisuc_1367 | lipoprotein | 217 | 31 | 78 | 9 | 1.03E-55 |  |
| Fisuc_1368 | lipoprotein | 247 | 21 | 127 | 12 | 6.65E-17 |  |
| Fisuc_1369* | rubredoxin-type Fe(Cys)4 protein | 213 | 26 | 120 | 9 | 8.78E-25 |  |
| Fisuc_1370 | hypothetical protein | 96 | 18 | 36 | 5 | 2.08E-73 |  |
| Fisuc_1371 | hypothetical protein | 87 | 25 | 31 | 4 | 2.87E-88 |  |
| Fisuc_1372 | hypothetical protein | 54 | 15 | 22 | 3 | 4.62E-65 |  |
| Fisuc_1373 | carbon starvation protein CstA | 47 | 7 | 25 | 2 | 2.26E-20 |  |
| Fisuc_1374 | aspartate kinase | 316 | 26 | 627 | 18 | 5.98E-11 |  |
| Fisuc_1375* | Fis family sigma-54 specific transcriptional regulator | 69 | 4 | 38 | 9 | 1.40E-17 |  |
| Fisuc_1379 | HipA N-terminal domain-containing protein | 36 | 5 | 63 | 2 | 3.75E-07 |  |
| Fisuc_1380* | transcriptional regulator, XRE family | 27 | 9 | 86 | 7 | 3.22E-69 |  |
| Fisuc_1384 | alanine racemase | 182 | 5 | 256 | 9 | 7.62E-04 |  |
| Fisuc_1385 | hypothetical protein | 43 | 8 | 66 | 5 | 1.02E-04 |  |
| Fisuc_1386 | hypothetical protein | 88 | 10 | 124 | 6 | 9.26E-04 |  |
| Fisuc_1389 | endopeptidase Clp | 421 | 74 | 693 | 64 | 2.31E-08 |  |
| Fisuc_1390 | trigger factor | 438 | 64 | 804 | 37 | 1.37E-04 |  |
| Fisuc_1391 | methyltransferase type 11 | 69 | 30 | 109 | 8 | 8.32E-09 |  |
| Fisuc_1395 | polysaccharide export periplasmic protein | 478 | 11 | 444 | 38 | 2.40E-04 |  |
| Fisuc_1396* | 50S ribosomal protein L17 | 2426 | 208 | 4438 | 297 | 7.76E-03 |  |
| Fisuc_1397* | DNA-directed RNA polymerase subunit alpha | 2797 | 380 | 6026 | 493 | 6.78E-04 |  |
| Fisuc_1398* | 30S ribosomal protein S11 | 2248 | 484 | 4226 | 409 | 9.25E-04 |  |
| Fisuc_1399* | 30S ribosomal protein S13 | 2750 | 697 | 5422 | 403 | 5.59E-04 |  |
| Fisuc_1400* | 50S ribosomal protein L36 | 2731 | 615 | 5455 | 431 | 1.82E-09 |  |
| Fisuc_1401* | translation initiation factor IF-1 | 1627 | 330 | 3245 | 234 | 4.09E-11 |  |
| Fisuc_1402 | preprotein translocase subunit SecY | 2182 | 329 | 6575 | 540 | 1.33E-06 |  |
| Fisuc_1403* | 50S ribosomal protein L15 | 2660 | 407 | 6019 | 438 | 1.39E-05 |  |
| Fisuc_1404* | 50S ribosomal protein L30 | 2917 | 524 | 5923 | 290 | 5.86E-08 |  |
| Fisuc_1405* | 30S ribosomal protein S5 | 2354 | 381 | 4901 | 249 | 4.46E-04 |  |
| Fisuc_1406* | 50S ribosomal protein L18 | 2805 | 459 | 5482 | 286 | 1.28E-03 |  |
| Fisuc_1407* | 50S ribosomal protein L6 | 3006 | 347 | 5912 | 294 | 4.98E-03 |  |
| Fisuc_1408* | 30S ribosomal protein S8 | 2477 | 207 | 4375 | 214 | 2.59E-03 |  |
| Fisuc_1409* | 30S ribosomal protein S14 | 2682 | 207 | 4798 | 295 | 3.07E-06 |  |
| Fisuc_1411* | 50S ribosomal protein L24 | 3075 | 231 | 5760 | 243 | 6.86E-05 |  |
| Fisuc_1412* | 50S ribosomal protein L14 | 2491 | 169 | 4526 | 239 | 1.36E-04 |  |
| Fisuc_1413* | 30S ribosomal protein S17 | 3075 | 248 | 5570 | 301 | 4.44E-06 |  |
| Fisuc_1414* | 50S ribosomal protein L29 | 3518 | 274 | 6573 | 291 | 1.32E-05 |  |
| Fisuc_1415* | 50S ribosomal protein L16 | 2461 | 183 | 4721 | 303 | 1.33E-03 |  |
| Fisuc_1416* | 30S ribosomal protein S3 | 2267 | 167 | 4528 | 277 | 3.30E-04 |  |
| Fisuc_1417* | 50S ribosomal protein L22 | 2173 | 167 | 4238 | 220 | 9.72E-08 |  |
| Fisuc_1418* | 30S ribosomal protein S19 | 2411 | 164 | 4639 | 262 | 9.48E-07 |  |
| Fisuc_1420* | 50S ribosomal protein L25 | 2702 | 207 | 5294 | 227 | 1.74E-07 |  |
| Fisuc_1422* | 50S ribosomal protein L3 | 2393 | 233 | 4716 | 260 | 3.27E-04 |  |
| Fisuc_1423* | 30S ribosomal protein S10 | 3070 | 428 | 5622 | 174 | 3.49E-04 |  |
| Fisuc_1425 | cellulase | 308 | 51 | 100 | 3 | 7.68E-73 | YES |
| Fisuc_1426 | cellulase | 424 | 143 | 99 | 5 | 5.70E-164 | YES |
| Fisuc_1428 | peptidase M48 Ste24p | 153 | 14 | 96 | 10 | 1.22E-07 |  |
| Fisuc_1433 | hypothetical protein | 38 | 6 | 57 | 8 | 1.57E-05 |  |
| Fisuc_1434 | hypothetical protein | 109 | 14 | 242 | 38 | 4.88E-26 |  |
| Fisuc_1435 | hypothetical protein | 151 | 13 | 348 | 36 | 2.32E-28 |  |
| Fisuc_1439 | group 1 glycosyl transferase | 405 | 13 | 343 | 10 | 7.22E-03 | YES |
| Fisuc_1440 | hypothetical protein | 1605 | 144 | 3030 | 319 | 4.16E-06 |  |
| Fisuc_1441 | metallophosphoesterase | 653 | 40 | 352 | 24 | 3.70E-21 |  |
| Fisuc_1446 | glucose inhibited division protein | 195 | 10 | 260 | 27 | 7.05E-03 |  |
| Fisuc_1447 | hypothetical protein | 152 | 23 | 47 | 7 | 5.44E-100 |  |
| Fisuc_1448 | hypothetical protein | 73 | 14 | 25 | 5 | 8.47E-105 |  |
| Fisuc_1449 | Glyoxalase/bleomycin resistance protein/dioxygenase | 81 | 16 | 25 | 5 | 1.65E-138 |  |
| Fisuc_1450 | hypothetical protein | 49 | 11 | 38 | 5 | 5.45E-08 |  |
| Fisuc_1451 | Appr-1-p processing protein | 54 | 14 | 43 | 7 | 1.07E-06 |  |
| Fisuc_1453 | hypothetical protein | 435 | 18 | 261 | 22 | 2.14E-12 |  |
| Fisuc_1455 | hypothetical protein | 106 | 15 | 177 | 29 | 7.22E-06 |  |
| Fisuc_1457 | bile acid:sodium symporter | 47 | 3 | 37 | 2 | 1.70E-03 |  |
| Fisuc_1459 | hypothetical protein | 33 | 5 | 28 | 2 | 1.57E-04 |  |
| Fisuc_1461 | Preprotein translocase subunit SecA | 237 | 13 | 557 | 62 | 1.25E-12 |  |
| Fisuc_1466 | hypothetical protein | 559 | 62 | 57 | 9 | 0 |  |
| Fisuc_1467 | hypothetical protein | 188 | 8 | 266 | 5 | 1.39E-03 |  |
| Fisuc_1468 | hypothetical protein | 118 | 14 | 93 | 10 | 6.01E-04 |  |
| Fisuc_1469 | hypothetical protein | 583 | 52 | 391 | 25 | 7.35E-06 |  |
| Fisuc_1471 | CarD family transcriptional regulator | 809 | 46 | 672 | 40 | 7.07E-07 |  |
| Fisuc_1473 | cellulase | 401 | 17 | 177 | 10 | 9.31E-40 | YES |
| Fisuc_1474 | fibro-slime family protein | 916 | 65 | 142 | 10 | 0 |  |
| Fisuc_1475 | fibro-slime family protein | 95 | 20 | 3 | 1 | 0 |  |
| Fisuc_1476 | hypothetical protein | 589 | 21 | 1303 | 60 | 5.66E-11 |  |
| Fisuc_1477 | ABC transporter | 85 | 10 | 145 | 5 | 1.15E-08 |  |
| Fisuc_1479 | hypothetical protein | 37 | 5 | 32 | 3 | 2.91E-05 |  |
| Fisuc_1480 | hypothetical protein | 66 | 8 | 43 | 2 | 4.61E-15 |  |
| Fisuc_1482 | hypothetical protein | 112 | 12 | 93 | 5 | 1.28E-03 |  |
| Fisuc_1483 | Tetraacyldisaccharide-1-P 4'-kinase | 42 | 7 | 34 | 3 | 2.50E-04 |  |
| Fisuc_1486* | rubrerythrin | 6556 | 462 | 3897 | 433 | 1.13E-05 |  |
| Fisuc_1487 | ferric uptake regulator, Fur family | 1988 | 154 | 999 | 170 | 8.88E-24 |  |
| Fisuc_1488 | TrkA-N domain-containing protein | 1239 | 81 | 635 | 118 | 1.04E-19 |  |
| Fisuc_1489 | H(+)-transporting two-sector ATPase | 227 | 27 | 110 | 23 | 7.30E-28 |  |
| Fisuc_1491* | NADH-ubiquinone/plastoquinone oxidoreductase chain 3 | 636 | 78 | 951 | 76 | 1.46E-05 |  |
| Fisuc_1492 | hypothetical protein | 51 | 7 | 98 | 5 | 2.35E-17 |  |
| Fisuc_1493 | ABC transporter | 41 | 4 | 59 | 4 | 1.02E-03 |  |
| Fisuc_1494 | hypothetical protein | 584 | 35 | 265 | 12 | 7.13E-21 |  |
| Fisuc_1496 | phosphoesterase PA-phosphatase-like protein | 39 | 11 | 58 | 8 | 4.24E-04 |  |
| Fisuc_1498 | hypothetical protein | 78 | 3 | 53 | 3 | 1.62E-08 |  |
| Fisuc_1501 | bifunctional 5,10-methylene-tetrahydrofolate dehydrogenase/ 5,10-methylene-tetrahydrofolate cyclohydrolase | 194 | 7 | 387 | 7 | 3.41E-18 |  |
| Fisuc_1502 | hypothetical protein | 272 | 36 | 1718 | 239 | 0 |  |
| Fisuc_1503 | FKBP-type peptidylprolyl isomerase | 319 | 17 | 252 | 40 | 2.68E-03 |  |
| Fisuc_1504 | amino-acid N-acetyltransferase | 342 | 6 | 262 | 6 | 3.46E-03 |  |
| Fisuc_1505 | hypothetical protein | 74 | 7 | 52 | 4 | 5.08E-05 |  |
| Fisuc_1506 | hypothetical protein | 32 | 4 | 25 | 3 | 2.97E-04 |  |
| Fisuc_1512 | GTP cyclohydrolase I | 298 | 30 | 549 | 26 | 5.39E-14 |  |
| Fisuc_1513 | esterase/lipase/thioesterase | 62 | 43 | 3 | 1 | 0 |  |
| Fisuc_1514 | esterase/lipase/thioesterase | 80 | 54 | 1 | 1 | 0 |  |
| Fisuc_1516* | Thiol:disulfide interchange protein-like protein | 76 | 2 | 106 | 7 | 1.02E-03 |  |
| Fisuc_1519 | hypothetical protein | 208 | 21 | 275 | 9 | 8.34E-03 |  |
| Fisuc_1521 | family 2 glycosyl transferase | 222 | 10 | 173 | 8 | 1.52E-03 | YES |
| Fisuc_1523 | glycoside hydrolase family protein | 314 | 95 | 102 | 3 | 1.58E-74 | YES |
| Fisuc_1524 | hypothetical protein | 5 | 2 | 3 | 1 | 2.77E-03 |  |
| Fisuc_1525 | hypothetical protein | 981 | 81 | 731 | 19 | 3.08E-10 | YES |
| Fisuc_1526 | hypothetical protein | 427 | 110 | 382 | 24 | 6.46E-03 |  |
| Fisuc_1529 | hypothetical protein | 762 | 47 | 657 | 20 | 4.80E-03 |  |
| Fisuc_1531 | cellulase | 53 | 8 | 22 | 3 | 9.30E-37 | YES |
| Fisuc_1532 | chorismate binding-like protein | 8 | 1 | 34 | 3 | 3.30E-107 |  |
| Fisuc_1533 | hypothetical protein | 39 | 6 | 21 | 3 | 2.03E-21 |  |
| Fisuc_1535 | ribulose-phosphate 3-epimerase | 299 | 21 | 605 | 28 | 2.49E-17 |  |
| Fisuc_1536 | glycoside hydrolase family protein | 800 | 59 | 1417 | 6 | 4.83E-06 |  |
| Fisuc_1537 | diaminopimelate decarboxylase | 111 | 21 | 199 | 11 | 1.41E-10 |  |
| Fisuc_1538 | hypothetical protein | 47 | 2 | 35 | 4 | 1.18E-07 |  |
| Fisuc_1539 | hypothetical protein | 66 | 8 | 60 | 2 | 6.61E-03 |  |
| Fisuc_1540 | hypothetical protein | 82 | 21 | 69 | 2 | 6.15E-03 |  |
| Fisuc_1541 | hypothetical protein | 40 | 8 | 30 | 3 | 4.30E-05 |  |
| Fisuc_1542 | hypothetical protein | 55 | 2 | 41 | 1 | 6.25E-04 |  |
| Fisuc_1543 | hypothetical protein | 14 | 2 | 22 | 3 | 1.33E-05 |  |
| Fisuc_1544 | serine/threonine protein kinase | 11 | 3 | 17 | 2 | 3.78E-03 |  |
| Fisuc_1545 | von Willebrand factor type A | 14 | 2 | 29 | 5 | 5.08E-17 |  |
| Fisuc_1546 | hypothetical protein | 10 | 2 | 24 | 1 | 8.56E-29 |  |
| Fisuc_1547 | radical SAM protein | 12 | 3 | 11 | 0 | 2.32E-04 |  |
| Fisuc_1548 | hypothetical protein | 2 | 1 | 4 | 1 | 5.95E-12 |  |
| Fisuc_1549 | hypothetical protein | 1 | 1 | 2 | 0 | 1.13E-26 |  |
| Fisuc_1550 | GTP-binding protein HSR1-like protein | 0 | 0 | 1 | 0 | 2.85E-53 |  |
| Fisuc_1551 | GTP-binding protein HSR1-like protein | 22 | 5 | 38 | 4 | 1.75E-09 |  |
| Fisuc_1552* | CRISPR-associated protein Cas1 | 54 | 9 | 22 | 2 | 5.76E-64 |  |
| Fisuc_1553* | CRISPR-associated protein Cas2 | 45 | 10 | 16 | 2 | 4.54E-44 |  |
| Fisuc_1554 | hypothetical protein | 93 | 9 | 29 | 3 | 2.86E-142 |  |
| Fisuc_1555 | hypothetical protein | 103 | 13 | 31 | 4 | 7.09E-149 |  |
| Fisuc_1556 | CRISPR-associated protein | 135 | 3 | 89 | 8 | 1.12E-07 |  |
| Fisuc_1557 | hypothetical protein | 138 | 4 | 85 | 5 | 4.71E-13 |  |
| Fisuc_1560* | hypothetical protein | 144 | 5 | 93 | 4 | 3.09E-11 |  |
| Fisuc_1561 | hypothetical protein | 110 | 3 | 68 | 4 | 8.26E-16 |  |
| Fisuc_1562* | hypothetical protein | 72 | 4 | 45 | 3 | 8.99E-08 |  |
| Fisuc_1564 | hypothetical protein | 43 | 3 | 86 | 4 | 6.39E-18 |  |
| Fisuc_1565 | hypothetical protein | 26 | 1 | 13 | 1 | 4.97E-37 |  |
| Fisuc_1568 | hypothetical protein | 84 | 46 | 6 | 1 | 0 | YES |
| Fisuc_1569 | Poly(3-hydroxybutyrate) depolymerase-like protein | 220 | 49 | 18 | 1 | 0 | YES |
| Fisuc_1572 | PTS transporter subunit IIA-like nitrogen-regulatory protein PtsN | 798 | 50 | 602 | 50 | 9.91E-06 |  |
| Fisuc_1574 | endopeptidase Clp | 931 | 61 | 830 | 43 | 4.76E-04 |  |
| Fisuc_1580 | hypothetical protein | 29 | 6 | 21 | 1 | 6.52E-10 |  |
| Fisuc_1582 | N-acetyltransferase GCN5 | 85 | 8 | 38 | 2 | 2.68E-48 |  |
| Fisuc_1583 | hypothetical protein | 63 | 9 | 34 | 6 | 4.75E-25 |  |
| Fisuc_1585 | hypothetical protein | 396 | 21 | 645 | 63 | 2.95E-07 |  |
| Fisuc_1586 | hypothetical protein | 36 | 8 | 30 | 1 | 1.85E-04 |  |
| Fisuc_1587 | response regulator receiver modulated diguanylate cyclase | 465 | 34 | 240 | 14 | 9.64E-21 |  |
| Fisuc_1588 | 2-dehydro-3-deoxyphosphogluconate aldolase | 155 | 16 | 117 | 10 | 1.36E-04 |  |
| Fisuc_1589 | hypothetical protein | 80 | 15 | 60 | 10 | 5.67E-05 |  |
| Fisuc_1590 | ABC transporter | 81 | 19 | 46 | 5 | 1.30E-14 |  |
| Fisuc_1593 | GtrA family protein | 59 | 1 | 100 | 2 | 4.87E-09 |  |
| Fisuc_1595 | phosphoglycerate mutase | 113 | 14 | 32 | 3 | 2.26E-106 |  |
| Fisuc_1596 | phosphoglycerate mutase | 41 | 11 | 35 | 2 | 8.18E-03 |  |
| Fisuc_1597 | hypothetical protein | 497 | 85 | 924 | 53 | 4.94E-09 |  |
| Fisuc_1598 | biotin/lipoyl attachment domain-containing protein | 686 | 151 | 1291 | 71 | 2.34E-12 |  |
| Fisuc_1600 | hypothetical protein | 6 | 2 | 14 | 1 | 2.03E-21 |  |
| Fisuc_1601 | hypothetical protein | 6 | 2 | 16 | 2 | 1.94E-27 |  |
| Fisuc_1602 | hypothetical protein | 14 | 2 | 35 | 5 | 3.87E-26 |  |
| Fisuc_1603 | hypothetical protein | 20 | 3 | 33 | 3 | 3.12E-10 |  |
| Fisuc_1604 | hypothetical protein | 15 | 2 | 22 | 3 | 2.47E-05 |  |
| Fisuc_1607 | hypothetical protein | 2 | 1 | 18 | 2 | 0 |  |
| Fisuc_1608 | hypothetical protein | 4 | 2 | 10 | 2 | 2.35E-32 |  |
| Fisuc_1609 | hypothetical protein | 6 | 2 | 21 | 3 | 2.24E-78 |  |
| Fisuc_1610 | hypothetical protein | 10 | 1 | 35 | 5 | 4.98E-102 |  |
| Fisuc_1611 | hypothetical protein | 12 | 1 | 33 | 3 | 2.70E-43 |  |
| Fisuc_1612 | hypothetical protein | 11 | 2 | 25 | 3 | 3.57E-23 |  |
| Fisuc_1613 | hypothetical protein | 4 | 1 | 8 | 1 | 1.84E-12 |  |
| Fisuc_1614* | protein serine/threonine phosphatase | 58 | 18 | 24 | 2 | 4.61E-36 |  |
| Fisuc_1616 | beta-lactamase | 1 | 1 | 0 | 0 | 9.22E-09 |  |
| Fisuc_1619 | acyl-ACP thioesterase | 48 | 6 | 37 | 3 | 1.90E-06 |  |
| Fisuc_1620 | 3-phosphoshikimate 1-carboxyvinyltransferase | 262 | 17 | 205 | 5 | 3.44E-05 |  |
| Fisuc_1621 | extracellular solute-binding protein | 133 | 8 | 103 | 4 | 1.14E-03 |  |
| Fisuc_1623 | hypothetical protein | 29 | 5 | 24 | 2 | 1.31E-04 |  |
| Fisuc_1639 | hypothetical protein | 96 | 5 | 62 | 3 | 8.52E-09 |  |
| Fisuc_1640 | hypothetical protein | 99 | 7 | 332 | 24 | 4.97E-73 |  |
| Fisuc_1641 | hypothetical protein | 120 | 10 | 84 | 9 | 1.09E-05 | YES |
| Fisuc_1644 | hypothetical protein | 286 | 30 | 164 | 9 | 7.21E-18 |  |
| Fisuc_1645 | hypothetical protein | 280 | 22 | 165 | 12 | 2.18E-15 |  |
| Fisuc_1648* | tRNA/rRNA methyltransferase SpoU | 408 | 7 | 605 | 42 | 7.70E-03 |  |
| Fisuc_1651 | hypothetical protein | 313 | 32 | 191 | 6 | 8.58E-13 |  |
| Fisuc_1652 | mammalian cell entry domain-containing protein | 410 | 18 | 315 | 30 | 4.32E-03 |  |
| Fisuc_1653 | aminopeptidase 2 | 105 | 6 | 72 | 6 | 5.53E-06 |  |
| Fisuc_1654 | acyltransferase 3 | 52 | 4 | 37 | 1 | 1.72E-05 |  |
| Fisuc_1656 | glycosyl hydrolase family 98 carbohydrate binding module | 28 | 6 | 37 | 5 | 1.29E-03 | YES |
| Fisuc_1658 | hypothetical protein | 151 | 7 | 317 | 18 | 2.39E-17 |  |
| Fisuc_1659 | hypothetical protein | 185 | 5 | 372 | 25 | 8.69E-17 |  |
| Fisuc_1660 | hypothetical protein | 271 | 10 | 789 | 53 | 8.80E-53 |  |
| Fisuc_1661 | glycoside hydrolase family protein | 40 | 5 | 28 | 4 | 3.80E-05 | YES |
| Fisuc_1664 | hypothetical protein | 103 | 37 | 10 | 2 | 0 |  |
| Fisuc_1665 | phosphoadenosine phosphosulfate reductase | 109 | 6 | 81 | 1 | 1.33E-06 |  |
| Fisuc_1671* | hypothetical protein | 41 | 4 | 64 | 5 | 1.46E-04 |  |
| Fisuc_1673* | RNA polymerase sigma 70 family subunit | 39 | 3 | 53 | 6 | 1.77E-03 |  |
| Fisuc_1674* | DtxR family iron (metal) dependent repressor | 673 | 21 | 1296 | 183 | 2.03E-07 |  |
| Fisuc_1675 | FeoA family protein | 455 | 26 | 666 | 125 | 1.20E-04 |  |
| Fisuc_1677 | hypothetical protein | 73 | 12 | 31 | 1 | 2.14E-40 |  |
| Fisuc_1679 | hypothetical protein | 1 | 1 | 1 | 0 | 5.34E-05 |  |
| Fisuc_1680* | flavodoxin | 0 | 0 | 1 | 1 | 3.83E-03 |  |
| Fisuc_1681 | hypothetical protein | 29 | 13 | 20 | 3 | 1.23E-04 |  |
| Fisuc_1682 | heavy metal translocating P-type ATPase | 19 | 3 | 12 | 0 | 9.46E-15 |  |
| Fisuc_1684 | exporter of the RND superfamily protein-like protein | 0 | 0 | 0 | 0 | 4.93E-21 |  |
| Fisuc_1685 | hypothetical protein | 0 | 0 | 2 | 1 | 0 |  |
| Fisuc_1686 | cobalt transport protein | 0 | 0 | 0 | 0 | 7.51E-13 |  |
| Fisuc_1687 | ABC transporter | 0 | 1 | 0 | 0 | 3.68E-21 |  |
| Fisuc_1688 | mannan endo-1,4-beta-mannosidase | 3 | 1 | 0 | 0 | 0 | YES |
| Fisuc_1691 | hypothetical protein | 92 | 14 | 185 | 36 | 1.83E-18 |  |
| Fisuc_1692 | Holliday junction ATP-dependent DNA helicase RuvA | 96 | 4 | 58 | 2 | 2.43E-12 |  |
| Fisuc_1695 | hypothetical protein | 51 | 5 | 33 | 4 | 6.59E-12 |  |
| Fisuc_1696 | hypothetical protein | 65 | 6 | 34 | 4 | 2.05E-31 |  |
| Fisuc_1697 | phosphate/sulfate permease-like protein | 2 | 1 | 1 | 0 | 3.08E-07 |  |
| Fisuc_1698* | two component transcriptional regulator, winged helix family | 45 | 2 | 36 | 1 | 2.63E-05 |  |
| Fisuc_1699 | integral membrane sensor signal transduction histidine kinase | 23 | 1 | 16 | 1 | 8.41E-11 |  |
| Fisuc_1702 | phosphate uptake regulator PhoU | 11 | 2 | 6 | 0 | 7.60E-11 |  |
| Fisuc_1704 | phosphate ABC transporter permease | 2 | 1 | 0 | 1 | 2.08E-32 |  |
| Fisuc_1705 | phosphate ABC transporter permease | 1 | 0 | 0 | 1 | 1.66E-07 |  |
| Fisuc_1706 | hypothetical protein | 2 | 1 | 1 | 1 | 5.74E-27 |  |
| Fisuc_1709 | aldo/keto reductase | 6 | 3 | 2 | 1 | 3.61E-35 |  |
| Fisuc_1711* | transcriptional regulator, LysR family | 83 | 12 | 61 | 3 | 1.62E-05 |  |
| Fisuc_1712 | antibiotic biosynthesis monooxygenase | 136 | 12 | 80 | 7 | 4.48E-18 |  |
| Fisuc_1713 | hypothetical protein | 148 | 15 | 83 | 11 | 1.90E-23 |  |
| Fisuc_1716 | hypothetical protein | 13 | 2 | 6 | 1 | 3.40E-41 |  |
| Fisuc_1717 | hypothetical protein | 36 | 5 | 13 | 3 | 2.81E-73 |  |
| Fisuc_1718 | NADPH-dependent FMN reductase | 32 | 8 | 13 | 1 | 3.09E-52 |  |
| Fisuc_1719 | hypothetical protein | 9 | 2 | 4 | 1 | 3.91E-18 |  |
| Fisuc_1720 | Type I site-specific deoxyribonuclease | 1 | 1 | 18 | 6 | 0 |  |
| Fisuc_1721 | restriction modification system DNA specificity domain-containing protein | 2 | 1 | 34 | 9 | 0 |  |
| Fisuc_1722 | adenine-specific DNA-methyltransferase | 36 | 2 | 65 | 12 | 2.13E-14 |  |
| Fisuc_1726 | ATPase | 21 | 4 | 17 | 1 | 5.42E-05 |  |
| Fisuc_1727 | zinc/iron permease | 33 | 4 | 25 | 1 | 3.55E-09 |  |
| Fisuc_1731 | ABC transporter | 0 | 0 | 1 | 1 | 9.06E-11 |  |
| Fisuc_1732 | hypothetical protein | 1 | 1 | 4 | 2 | 4.90E-37 |  |
| Fisuc_1733 | TonB-dependent receptor plug | 0 | 0 | 0 | 0 | 2.33E-11 |  |
| Fisuc_1735 | small GTP-binding protein | 0 | 0 | 0 | 0 | 5.45E-03 |  |
| Fisuc_1736 | TonB family protein | 0 | 0 | 0 | 1 | 2.52E-48 |  |
| Fisuc_1737 | MotA/TolQ/ExbB proton channel | 0 | 0 | 0 | 1 | 1.95E-28 |  |
| Fisuc_1738 | biopolymer transport protein ExbD/TolR | 0 | 0 | 0 | 0 | 1.78E-04 |  |
| Fisuc_1744 | diguanylate cyclase | 121 | 19 | 88 | 5 | 3.57E-07 |  |
| Fisuc_1746 | DivIVA domain-containing protein | 308 | 7 | 257 | 10 | 1.64E-03 |  |
| Fisuc_1747* | 50S ribosomal protein L34 | 1811 | 151 | 5325 | 556 | 5.92E-38 |  |
| Fisuc_1748 | hypothetical protein | 594 | 38 | 1413 | 91 | 2.31E-30 |  |
| Fisuc_1749 | hypothetical protein | 302 | 27 | 761 | 24 | 3.79E-30 |  |
| Fisuc_1750 | YidC/Oxa1 family membrane protein insertase | 155 | 18 | 369 | 17 | 6.66E-20 |  |
| Fisuc_1751 | glycoside hydrolase family protein | 84 | 10 | 29 | 4 | 2.05E-81 | YES |
| Fisuc_1752 | peptidase U61 LD-carboxypeptidase A | 86 | 18 | 44 | 6 | 6.49E-22 |  |
| Fisuc_1753 | hypothetical protein | 102 | 22 | 75 | 8 | 4.30E-06 |  |
| Fisuc_1756 | FKBP-type peptidylprolyl isomerase | 156 | 52 | 236 | 22 | 9.45E-06 |  |
| Fisuc_1758 | hypothetical protein | 76 | 6 | 104 | 5 | 2.81E-03 |  |
| Fisuc_1759 | hypothetical protein | 70 | 3 | 107 | 7 | 3.13E-06 |  |
| Fisuc_1760 | hypothetical protein | 527 | 218 | 106 | 3 | 2.57E-211 |  |
| Fisuc_1761 | hypothetical protein | 857 | 202 | 391 | 25 | 3.60E-35 |  |
| Fisuc_1762 | glycoside hydrolase family protein | 489 | 136 | 15 | 1 | 0 | YES |
| Fisuc_1763 | carbohydrate binding family 6 | 509 | 238 | 2 | 1 | 0 | YES |
| Fisuc_1764 | carbohydrate binding family 6 | 175 | 95 | 1 | 1 | 0 | YES |
| Fisuc_1765 | glucuronoarabinoxylan endo-1,4-beta-xylanase | 77 | 30 | 3 | 1 | 0 | YES |
| Fisuc_1766 | carbohydrate binding family 6 | 10 | 5 | 1 | 1 | 0 | YES |
| Fisuc_1767 | carbohydrate binding family 6 | 180 | 61 | 4 | 1 | 0 | YES |
| Fisuc_1768 | feruloyl esterase | 4 | 1 | 2 | 1 | 5.64E-06 | YES |
| Fisuc_1769 | xylan 1,4-beta-xylosidase | 257 | 111 | 5 | 1 | 0 | YES |
| Fisuc_1770 | hypothetical protein | 18 | 7 | 0 | 1 | 0 |  |
| Fisuc_1771 | carbohydrate-binding CenC domain protein | 18 | 6 | 2 | 0 | 0 | YES |
| Fisuc_1772 | hypothetical protein | 28 | 1 | 15 | 2 | 1.52E-22 |  |
| Fisuc_1773 | alpha-galactosidase | 571 | 141 | 4 | 1 | 0 | YES |
| Fisuc_1774 | carbohydrate binding family 6 | 493 | 125 | 2 | 2 | 0 | YES |
| Fisuc_1775 | glycoside hydrolase family protein | 377 | 133 | 2 | 1 | 0 | YES |
| Fisuc_1776 | hypothetical protein | 67 | 15 | 121 | 28 | 2.20E-11 |  |
| Fisuc_1779 | lipoprotein | 57 | 8 | 29 | 3 | 3.08E-32 |  |
| Fisuc_1780 | hypothetical protein | 85 | 9 | 42 | 1 | 3.49E-24 |  |
| Fisuc_1781 | hypothetical protein | 75 | 6 | 55 | 4 | 1.98E-10 |  |
| Fisuc_1782 | hypothetical protein | 95 | 5 | 78 | 3 | 2.85E-03 |  |
| Fisuc_1783* | UvrD/REP helicase | 96 | 7 | 80 | 7 | 4.56E-04 |  |
| Fisuc_1785 | hypothetical protein | 98 | 5 | 66 | 5 | 3.94E-16 |  |
| Fisuc_1786 | hypothetical protein | 90 | 10 | 68 | 11 | 2.22E-04 |  |
| Fisuc_1787 | hypothetical protein | 67 | 7 | 36 | 5 | 4.61E-32 |  |
| Fisuc_1788 | family 2 glycoside hydrolase | 924 | 251 | 6 | 2 | 0 | YES |
| Fisuc_1790 | carbohydrate binding family 6 | 220 | 88 | 2 | 0 | 0 | YES |
| Fisuc_1791 | carbohydrate binding family 6 | 109 | 31 | 4 | 1 | 0 | YES |
| Fisuc_1792 | hypothetical protein | 10 | 4 | 9 | 2 | 7.27E-03 |  |
| Fisuc_1793 | carbohydrate binding family 6 | 471 | 200 | 10 | 2 | 0 | YES |
| Fisuc_1794 | glycoside hydrolase family protein | 63 | 28 | 1 | 0 | 0 | YES |
| Fisuc_1795 | histidine kinase | 216 | 22 | 58 | 9 | 1.88E-122 |  |
| Fisuc_1796* | response regulator receiver modulated metal dependent phosphohydrolase | 307 | 14 | 142 | 10 | 7.11E-30 |  |
| Fisuc_1797 | hypothetical protein | 42 | 3 | 77 | 3 | 2.41E-15 |  |
| Fisuc_1798 | histidine kinase | 103 | 13 | 75 | 3 | 1.37E-04 |  |
| Fisuc_1799 | Hpt protein | 127 | 17 | 73 | 6 | 3.06E-20 |  |
| Fisuc_1800* | response regulator receiver modulated metal dependent phosphohydrolase | 63 | 12 | 27 | 3 | 3.03E-31 |  |
| Fisuc_1801 | histidine kinase | 85 | 29 | 7 | 2 | 0 |  |
| Fisuc_1802 | glycoside hydrolase family protein | 920 | 302 | 305 | 37 | 2.76E-58 | YES |
| Fisuc_1803 | polysaccharide deacetylase | 541 | 37 | 26 | 9 | 0 |  |
| Fisuc_1804 | sugar transporter | 132 | 4 | 75 | 10 | 1.89E-11 |  |
| Fisuc_1806 | hypothetical protein | 589 | 75 | 459 | 25 | 1.15E-03 |  |
| Fisuc_1807 | hypothetical protein | 329 | 50 | 197 | 4 | 1.84E-13 |  |
| Fisuc_1808 | hypothetical protein | 258 | 67 | 155 | 8 | 1.11E-08 |  |
| Fisuc_1810 | hypothetical protein | 148 | 17 | 74 | 4 | 3.88E-42 |  |
| Fisuc_1813 | diaminopimelate epimerase | 184 | 22 | 337 | 144 | 9.52E-12 |  |
| Fisuc_1814 | LL-diaminopimelate aminotransferase | 105 | 12 | 76 | 55 | 5.22E-05 |  |
| Fisuc_1816 | glutamine synthetase | 53 | 14 | 25 | 16 | 9.66E-31 |  |
| Fisuc_1817 | glutamate synthase | 60 | 19 | 48 | 28 | 2.53E-04 |  |
| Fisuc_1818 | glutamate synthase NADH/NADPH small subunit | 1 | 0 | 8 | 3 | 2.85E-289 |  |
| Fisuc_1820 | ammonium transporter | 6 | 2 | 4 | 2 | 3.62E-03 |  |
| Fisuc_1823 | hypothetical protein | 16 | 4 | 14 | 2 | 4.09E-04 |  |
| Fisuc_1833 | nucleic acid binding protein | 52 | 7 | 40 | 1 | 2.15E-07 |  |
| Fisuc_1834* | RelB/DinJ family addiction module antitoxin | 62 | 13 | 51 | 4 | 8.94E-07 |  |
| Fisuc_1835 | hypothetical protein | 8 | 3 | 4 | 1 | 7.27E-19 |  |
| Fisuc_1836 | hypothetical protein | 148 | 16 | 79 | 9 | 2.69E-26 |  |
| Fisuc_1837 | hypothetical protein | 118 | 23 | 73 | 5 | 1.94E-19 |  |
| Fisuc_1838 | hypothetical protein | 368 | 49 | 290 | 29 | 7.25E-04 |  |
| Fisuc_1840 | hypothetical protein | 29 | 11 | 19 | 1 | 1.99E-14 |  |
| Fisuc_1841 | hypothetical protein | 49 | 8 | 26 | 1 | 8.49E-34 |  |
| Fisuc_1842 | hypothetical protein | 17 | 4 | 15 | 4 | 1.27E-04 |  |
| Fisuc_1843 | hypothetical protein | 475 | 114 | 176 | 27 | 6.16E-51 |  |
| Fisuc_1844 | hypothetical protein | 100 | 12 | 48 | 14 | 4.42E-28 |  |
| Fisuc_1845 | hypothetical protein | 190 | 17 | 28 | 12 | 0 |  |
| Fisuc_1846 | M6 family metalloprotease domain-containing protein | 73 | 10 | 4 | 1 | 0 |  |
| Fisuc_1847 | uridylate kinase | 145 | 13 | 208 | 15 | 2.47E-03 |  |
| Fisuc_1849 | hypothetical protein | 21 | 2 | 90 | 13 | 2.24E-131 |  |
| Fisuc_1852 | phosphatidate cytidylyltransferase | 211 | 28 | 166 | 11 | 2.00E-04 |  |
| Fisuc_1853 | hypothetical protein | 20 | 3 | 5 | 0 | 8.60E-141 |  |
| Fisuc_1854 | hypothetical protein | 17 | 6 | 2 | 1 | 1.68E-280 |  |
| Fisuc_1855 | hypothetical protein | 27 | 12 | 2 | 1 | 0 |  |
| Fisuc_1856 | hypothetical protein | 9 | 4 | 0 | 0 | 0 |  |
| Fisuc_1857 | 1-deoxy-D-xylulose 5-phosphate reductoisomerase | 232 | 8 | 350 | 18 | 2.42E-04 |  |
| Fisuc_1858 | Esterase/lipase-like protein | 15 | 2 | 8 | 0 | 6.47E-30 |  |
| Fisuc_1859 | glycoside hydrolase family protein | 487 | 95 | 7 | 2 | 0 | YES |
| Fisuc_1860 | glycoside hydrolase family protein | 442 | 30 | 17 | 2 | 0 | YES |
| Fisuc_1862 | cupin | 188 | 30 | 261 | 21 | 1.21E-03 |  |
| Fisuc_1869 | protein-S-isoprenylcysteine methyltransferase-like protein | 77 | 9 | 57 | 2 | 3.63E-08 |  |
| Fisuc_1870 | N-acetyltransferase GCN5 | 136 | 6 | 109 | 2 | 3.76E-03 |  |
| Fisuc_1871 | hypothetical protein | 143 | 12 | 121 | 4 | 7.31E-03 |  |
| Fisuc_1873 | phosphoribosylaminoimidazole carboxylase catalytic subunit | 114 | 20 | 217 | 18 | 4.89E-18 |  |
| Fisuc_1875 | hypothetical protein | 905 | 53 | 395 | 71 | 1.50E-28 |  |
| Fisuc_1876* | MiaB-like tRNA modifying enzyme YliG | 73 | 11 | 114 | 2 | 2.58E-05 |  |
| Fisuc_1880* | 30S ribosomal protein S21 | 2943 | 289 | 7291 | 501 | 6.95E-15 |  |
| Fisuc_1884 | hypothetical protein | 62 | 5 | 88 | 6 | 2.42E-04 |  |
| Fisuc_1885 | hypothetical protein | 175 | 34 | 288 | 28 | 7.71E-08 |  |
| Fisuc_1888 | hypothetical protein | 47 | 4 | 61 | 6 | 7.19E-03 |  |
| Fisuc_1890 | hypothetical protein | 363 | 114 | 92 | 3 | 3.35E-231 |  |
| Fisuc_1891 | OmpA/MotB domain-containing protein | 923 | 330 | 525 | 23 | 8.61E-11 |  |
| Fisuc_1892 | hypothetical protein | 1093 | 280 | 499 | 20 | 1.58E-22 |  |
| Fisuc_1893 | hypothetical protein | 982 | 81 | 577 | 16 | 6.48E-06 |  |
| Fisuc_1894 | MotA/TolQ/ExbB proton channel | 2649 | 325 | 922 | 6 | 1.59E-83 |  |
| Fisuc_1895 | biopolymer transport protein ExbD/TolR | 1752 | 351 | 635 | 13 | 7.52E-75 |  |
| Fisuc_1896 | biopolymer transport protein ExbD/TolR | 1101 | 332 | 370 | 12 | 1.09E-64 |  |
| Fisuc_1897 | TonB family protein | 424 | 219 | 178 | 28 | 2.29E-35 |  |
| Fisuc_1903 | penicillin-binding protein 2 | 11 | 4 | 21 | 3 | 9.34E-16 |  |
| Fisuc_1904 | rod shape-determining protein RodA | 32 | 9 | 44 | 4 | 1.39E-03 |  |
| Fisuc_1907 | spore coat protein CotH | 307 | 72 | 103 | 11 | 3.50E-78 |  |
| Fisuc_1912 | hypothetical protein | 22 | 1 | 59 | 10 | 8.06E-34 |  |
| Fisuc_1913 | biotin--acetyl-CoA-carboxylase ligase | 138 | 7 | 98 | 4 | 5.23E-05 |  |
| Fisuc_1914 | hypothetical protein | 388 | 29 | 318 | 12 | 5.27E-04 |  |
| Fisuc_1916 | hypothetical protein | 210 | 17 | 149 | 9 | 2.38E-07 |  |
| Fisuc_1917 | Cell division protein-like protein | 160 | 10 | 121 | 13 | 1.52E-03 |  |
| Fisuc_1918 | peptidase M23 | 130 | 3 | 83 | 6 | 9.65E-08 |  |
| Fisuc_1919* | ATPase AAA | 270 | 10 | 212 | 16 | 1.90E-05 |  |
| Fisuc_1921 | hypothetical protein | 129 | 14 | 107 | 11 | 2.73E-03 |  |
| Fisuc_1922 | hypothetical protein | 378 | 29 | 919 | 54 | 8.24E-32 |  |
| Fisuc_1924 | hypothetical protein | 205 | 13 | 138 | 17 | 1.53E-08 |  |
| Fisuc_1925 | hypothetical protein | 87 | 6 | 64 | 6 | 1.32E-04 |  |
| Fisuc_1926 | hypothetical protein | 143 | 8 | 99 | 5 | 7.77E-07 |  |
| Fisuc_1927 | hypothetical protein | 88 | 9 | 54 | 3 | 1.89E-13 |  |
| Fisuc_1930 | hypothetical protein | 263 | 51 | 34 | 1 | 0 |  |
| Fisuc_1931 | carbohydrate-binding CenC domain protein | 279 | 43 | 58 | 7 | 4.93E-233 | YES |
| Fisuc_1934 | hypothetical protein | 104 | 32 | 25 | 2 | 1.59E-156 |  |
| Fisuc_1935 | hypothetical protein | 42 | 4 | 34 | 2 | 1.63E-05 |  |
| Fisuc_1936* | DEAD/DEAH box helicase | 41 | 2 | 101 | 3 | 2.13E-28 |  |
| Fisuc_1939 | three-deoxy-D-manno-octulosonic-acid transferase domain-containing protein | 110 | 7 | 83 | 4 | 1.92E-03 | YES |
| Fisuc_1940 | hydroxymethylbutenyl pyrophosphate reductase | 60 | 20 | 77 | 7 | 6.62E-03 |  |
| Fisuc_1941* | 50S ribosomal protein L28 | 3143 | 514 | 6670 | 558 | 1.14E-09 |  |
| Fisuc_1942* | DNA polymerase III subunit epsilon | 379 | 27 | 691 | 99 | 5.53E-11 |  |
| Fisuc_1943 | hypothetical protein | 66 | 4 | 54 | 3 | 1.73E-05 |  |
| Fisuc_1946 | hypothetical protein | 229 | 5 | 23 | 5 | 0 |  |
| Fisuc_1948 | hypothetical protein | 896 | 325 | 4 | 2 | 0 | YES |
| Fisuc_1949 | ABC transporter | 54 | 10 | 90 | 6 | 9.97E-07 |  |
| Fisuc_1950 | CrcB protein | 26 | 3 | 59 | 4 | 3.93E-24 |  |
| Fisuc_1957 | hypothetical protein | 138 | 8 | 262 | 18 | 3.33E-13 |  |
| Fisuc_1958* | protein-(glutamine-N5) methyltransferase | 164 | 17 | 289 | 10 | 1.66E-10 |  |
| Fisuc_1959* | peptide chain release factor 1 | 103 | 28 | 220 | 10 | 1.53E-19 |  |
| Fisuc_1961 | hypothetical protein | 85 | 5 | 223 | 11 | 2.53E-33 |  |
| Fisuc_1962 | hypothetical protein | 172 | 3 | 508 | 36 | 7.78E-51 |  |
| Fisuc_1963 | hypothetical protein | 131 | 7 | 377 | 25 | 1.27E-49 |  |
| Fisuc_1964 | hypothetical protein | 181 | 20 | 342 | 28 | 7.95E-16 |  |
| Fisuc_1967 | hypothetical protein | 32 | 5 | 48 | 8 | 4.25E-05 |  |
| Fisuc_1968 | hypothetical protein | 21 | 8 | 32 | 2 | 1.52E-03 |  |
| Fisuc_1969 | dTDP-4-dehydrorhamnose reductase | 10 | 2 | 18 | 2 | 2.09E-06 |  |
| Fisuc_1970* | NUDIX hydrolase | 27 | 2 | 55 | 3 | 5.80E-16 |  |
| Fisuc_1971 | hypothetical protein | 6 | 1 | 15 | 0 | 5.73E-31 |  |
| Fisuc_1972 | hypothetical protein | 8 | 1 | 21 | 3 | 4.06E-30 |  |
| Fisuc_1973 | hypothetical protein | 11 | 1 | 23 | 2 | 2.93E-15 |  |
| Fisuc_1974 | polysaccharide deacetylase | 44 | 2 | 1 | 1 | 0 |  |
| Fisuc_1977 | MATE efflux family protein | 74 | 3 | 167 | 11 | 3.26E-22 |  |
| Fisuc_1978 | diguanylate cyclase | 74 | 4 | 145 | 10 | 8.58E-16 |  |
| Fisuc_1979 | fibro-slime family protein | 829 | 268 | 159 | 19 | 8.37E-167 |  |
| Fisuc_1983 | hypothetical protein | 45 | 4 | 11 | 1 | 1.14E-161 |  |
| Fisuc_1987 | family 2 glycosyl transferase | 187 | 8 | 150 | 20 | 4.36E-03 | YES |
| Fisuc_1988 | hypothetical protein | 87 | 17 | 57 | 8 | 4.85E-17 |  |
| Fisuc_1990 | hypothetical protein | 81 | 12 | 30 | 5 | 1.00E-55 |  |
| Fisuc_1991 | pectate lyase-like protein | 31 | 13 | 1 | 0 | 0 | YES |
| Fisuc_1992 | hypothetical protein | 49 | 13 | 17 | 2 | 2.05E-106 |  |
| Fisuc_1994 | carbohydrate binding family 6 | 2 | 2 | 3 | 1 | 5.80E-03 | YES |
| Fisuc_1995 | hypothetical protein | 5 | 1 | 8 | 1 | 3.30E-03 | YES |
| Fisuc_1996 | arabinogalactan endo-1,4-beta-galactosidase | 4 | 1 | 6 | 1 | 8.98E-03 | YES |
| Fisuc_2000 | hypothetical protein | 35 | 5 | 28 | 2 | 3.29E-04 |  |
| Fisuc_2001 | hypothetical protein | 56 | 6 | 42 | 2 | 8.91E-04 |  |
| Fisuc_2002 | hypothetical protein | 6 | 4 | 1 | 1 | 0 | YES |
| Fisuc_2004 | spore coat protein CotH | 10 | 2 | 4 | 1 | 3.78E-38 |  |
| Fisuc_2005 | FG-GAP repeat-containing protein | 10 | 5 | 1 | 1 | 0 | YES |
| Fisuc_2006 | hypothetical protein | 2 | 1 | 0 | 0 | 1.70E-100 |  |
| Fisuc_2007 | hypothetical protein | 18 | 1 | 31 | 5 | 1.34E-07 |  |
| Fisuc_2009 | hypothetical protein | 31 | 8 | 25 | 2 | 1.17E-06 |  |
| Fisuc_2010 | hypothetical protein | 27 | 4 | 17 | 2 | 2.46E-16 |  |
| Fisuc_2011 | cellulase | 56 | 12 | 31 | 2 | 5.70E-16 | YES |
| Fisuc_2012 | hypothetical protein | 56 | 17 | 15 | 1 | 5.10E-166 | YES |
| Fisuc_2013 | hypothetical protein | 933 | 56 | 2412 | 46 | 3.50E-23 |  |
| Fisuc_2020 | hypothetical protein | 225 | 46 | 693 | 60 | 1.26E-56 |  |
| Fisuc_2021 | hypothetical protein | 432 | 44 | 224 | 9 | 2.41E-19 |  |
| Fisuc_2022 | hypothetical protein | 177 | 14 | 108 | 6 | 1.72E-18 |  |
| Fisuc_2023 | anion transporter | 209 | 57 | 379 | 19 | 4.84E-06 |  |
| Fisuc_2024 | hypothetical protein | 17 | 1 | 32 | 3 | 2.71E-12 |  |
| Fisuc_2027 | peptidase A24A domain-containing protein | 104 | 11 | 62 | 5 | 5.35E-14 |  |
| Fisuc_2028 | RND family efflux transporter MFP subunit | 245 | 30 | 195 | 20 | 2.58E-05 |  |
| Fisuc_2029 | cation diffusion facilitator family transporter | 173 | 14 | 96 | 7 | 3.46E-13 |  |
| Fisuc_2030 | aldo/keto reductase | 273 | 14 | 41 | 12 | 0 |  |
| Fisuc_2031 | fibro-slime family protein | 122 | 9 | 88 | 8 | 6.33E-06 |  |
| Fisuc_2032 | pyruvate carboxyltransferase | 1045 | 52 | 1922 | 592 | 1.55E-03 |  |
| Fisuc_2033 | glycoside hydrolase family protein | 73 | 19 | 12 | 2 | 0 | YES |
| Fisuc_2034 | hypothetical protein | 180 | 13 | 75 | 7 | 1.34E-34 |  |
| Fisuc_2036 | hypothetical protein | 82 | 4 | 35 | 2 | 1.24E-56 |  |
| Fisuc_2040 | ATPase AAA | 35 | 2 | 28 | 2 | 2.00E-03 |  |
| Fisuc_2042 | hypothetical protein | 134 | 3 | 75 | 5 | 1.95E-14 |  |
| Fisuc_2043 | hypothetical protein | 32 | 3 | 20 | 2 | 7.39E-15 |  |
| Fisuc_2044 | hypothetical protein | 26 | 2 | 16 | 1 | 1.88E-15 |  |
| Fisuc_2045 | glycosyltransferase family protein | 46 | 3 | 23 | 1 | 4.69E-35 | YES |
| Fisuc_2046 | family 2 glycosyl transferase | 47 | 11 | 24 | 2 | 3.84E-34 | YES |
| Fisuc_2047 | hydrolase (HAD superfamily)-like protein | 54 | 13 | 28 | 2 | 1.64E-21 |  |
| Fisuc_2048 | family 2 glycosyl transferase | 76 | 13 | 53 | 5 | 7.99E-08 | YES |
| Fisuc_2049 | hypothetical protein | 90 | 12 | 65 | 2 | 9.68E-07 |  |
| Fisuc_2050 | hypothetical protein | 73 | 3 | 53 | 2 | 5.90E-05 |  |
| Fisuc_2053 | hypothetical protein | 39 | 9 | 23 | 3 | 2.04E-22 |  |
| Fisuc_2054 | polysaccharide biosynthesis protein | 55 | 9 | 32 | 4 | 8.71E-14 |  |
| Fisuc_2055 | hypothetical protein | 76 | 9 | 42 | 5 | 5.99E-26 |  |
| Fisuc_2056 | hypothetical protein | 76 | 12 | 51 | 6 | 1.42E-12 |  |
| Fisuc_2057 | hypothetical protein | 74 | 8 | 62 | 7 | 2.86E-06 |  |
| Fisuc_2060 | phosphoribosylglycinamide synthetase | 73 | 8 | 44 | 4 | 3.36E-13 |  |
| Fisuc_2061 | TDP-4-oxo-6-deoxy-D-glucose transaminase | 82 | 7 | 57 | 3 | 4.45E-07 |  |
| Fisuc_2062 | hypothetical protein | 2 | 0 | 1 | 1 | 1.23E-04 |  |
| Fisuc_2063* | transcriptional regulator | 6 | 1 | 2 | 0 | 2.23E-42 |  |
| Fisuc_2064 | XRE family plasmid maintenance system antidote protein | 39 | 5 | 18 | 3 | 1.64E-48 |  |
| Fisuc_2065 | glycoside hydrolase family protein | 77 | 10 | 25 | 5 | 9.84E-83 | YES |
| Fisuc_2066 | hypothetical protein | 142 | 40 | 88 | 9 | 3.24E-10 |  |
| Fisuc_2067 | starch synthase catalytic domain-containing protein | 479 | 22 | 437 | 15 | 4.76E-04 | YES |
| Fisuc_2068 | hypothetical protein | 961 | 216 | 410 | 21 | 7.20E-35 |  |
| Fisuc_2071 | hypothetical protein | 18 | 9 | 31 | 7 | 5.01E-10 |  |
| Fisuc_2072 | hypothetical protein | 45 | 19 | 83 | 9 | 2.21E-15 |  |
| Fisuc_2073* | DNA replication and repair protein RecF | 36 | 12 | 66 | 8 | 4.45E-16 |  |
| Fisuc_2074 | large conductance mechanosensitive channel protein | 813 | 90 | 633 | 37 | 9.02E-05 |  |
| Fisuc_2075 | hypothetical protein | 18 | 2 | 40 | 2 | 1.23E-22 |  |
| Fisuc_2077 | hypothetical protein | 61 | 7 | 47 | 2 | 6.65E-09 |  |
| Fisuc_2078 | hypothetical protein | 37 | 7 | 14 | 1 | 2.37E-78 |  |
| Fisuc_2079 | hypothetical protein | 37 | 12 | 4 | 1 | 0 |  |
| Fisuc_2081 | coagulation factor 5/8 type domain-containing protein | 94 | 18 | 65 | 1 | 1.28E-06 | YES |
| Fisuc_2082 | acyltransferase 3 | 70 | 15 | 28 | 3 | 1.61E-64 |  |
| Fisuc_2083 | acid phosphatase | 83 | 5 | 54 | 3 | 7.43E-09 |  |
| Fisuc_2084 | hypothetical protein | 83 | 6 | 49 | 6 | 2.47E-13 |  |
| Fisuc_2085 | XRE family plasmid maintenance system antidote protein | 118 | 12 | 69 | 3 | 7.85E-13 |  |
| Fisuc_2086 | killer suppression protein HigA | 53 | 5 | 22 | 2 | 2.01E-47 |  |
| Fisuc_2087 | hypothetical protein | 46 | 3 | 21 | 2 | 5.52E-52 |  |
| Fisuc_2088* | threonyl-tRNA synthetase | 317 | 37 | 585 | 33 | 9.39E-05 |  |
| Fisuc_2089 | diguanylate cyclase | 81 | 16 | 22 | 5 | 5.49E-163 |  |
| Fisuc_2090 | hypothetical protein | 98 | 16 | 54 | 2 | 2.10E-18 |  |
| Fisuc_2091* | rubredoxin-type Fe(Cys)4 protein | 359 | 36 | 13 | 5 | 0 |  |
| Fisuc_2092 | 4'-phosphopantetheinyl transferase | 90 | 6 | 59 | 2 | 9.22E-10 |  |
| Fisuc_2093 | licheninase | 83 | 8 | 44 | 3 | 2.79E-20 | YES |
| Fisuc_2094 | ybaK/ebsC protein | 79 | 5 | 63 | 5 | 2.86E-05 |  |
| Fisuc_2095 | PAS/PAC sensor-containing diguanylate cyclase | 197 | 15 | 108 | 7 | 5.60E-13 |  |
| Fisuc_2096 | oxidoreductase domain-containing protein | 73 | 6 | 53 | 4 | 5.64E-06 |  |
| Fisuc_2098* | hypothetical protein | 41 | 6 | 21 | 1 | 1.33E-28 |  |
| Fisuc_2099 | hypothetical protein | 39 | 6 | 24 | 1 | 2.48E-15 |  |
| Fisuc_2101 | hypothetical protein | 10 | 2 | 15 | 1 | 1.29E-04 |  |
| Fisuc_2103 | hypothetical protein | 13 | 4 | 11 | 1 | 1.11E-04 |  |
| Fisuc_2104 | hypothetical protein | 190 | 19 | 6 | 1 | 0 |  |
| Fisuc_2105 | hypothetical protein | 158 | 16 | 6 | 2 | 0 |  |
| Fisuc_2106 | hypothetical protein | 98 | 12 | 5 | 1 | 0 |  |
| Fisuc_2107 | hypothetical protein | 39 | 4 | 8 | 0 | 6.20E-301 |  |
| Fisuc_2108 | hypothetical protein | 12 | 3 | 6 | 1 | 7.32E-20 |  |
| Fisuc_2109 | ATPase AAA | 7 | 1 | 12 | 2 | 4.26E-04 |  |
| Fisuc_2111 | phosphoglycerate mutase | 345 | 23 | 195 | 19 | 1.27E-15 |  |
| Fisuc_2112 | NAD-binding domain 4 protein | 48 | 8 | 30 | 2 | 6.91E-15 |  |
| Fisuc_2114 | hypothetical protein | 46 | 8 | 186 | 26 | 1.16E-127 |  |
| Fisuc_2116 | Cl- channel voltage-gated family protein | 30 | 3 | 55 | 3 | 2.99E-14 |  |
| Fisuc_2117 | spore coat protein CotH | 2 | 1 | 0 | 1 | 5.03E-70 |  |
| Fisuc_2118 | amino acid-binding ACT protein | 334 | 18 | 273 | 3 | 7.68E-03 |  |
| Fisuc_2120 | outer membrane protein assembly complex, YaeT protein | 249 | 32 | 489 | 30 | 1.82E-07 |  |
| Fisuc_2121 | outer membrane chaperone Skp | 347 | 7 | 643 | 4 | 2.13E-14 |  |
| Fisuc_2124 | hypothetical protein | 281 | 20 | 164 | 4 | 2.33E-16 |  |
| Fisuc_2125 | hypothetical protein | 278 | 18 | 226 | 20 | 3.05E-03 |  |
| Fisuc_2129* | NADH dehydrogenase (ubiquinone) 24 kDa subunit | 270 | 46 | 409 | 26 | 1.35E-03 |  |
| Fisuc_2130* | NADH dehydrogenase (quinone) | 131 | 30 | 214 | 16 | 8.67E-08 |  |
| Fisuc_2131* | NADH dehydrogenase (quinone) | 194 | 52 | 294 | 27 | 4.85E-03 |  |
| Fisuc_2132* | NADH dehydrogenase (quinone) | 203 | 75 | 329 | 29 | 2.36E-05 |  |
| Fisuc_2133* | 4Fe-4S ferredoxin | 199 | 49 | 299 | 34 | 2.76E-05 |  |
| Fisuc_2134* | NADH-ubiquinone/plastoquinone oxidoreductase chain 6 | 161 | 37 | 263 | 20 | 1.46E-05 |  |
| Fisuc_2135* | NADH-ubiquinone oxidoreductase chain 4L | 150 | 44 | 242 | 18 | 1.49E-09 |  |
| Fisuc_2136* | proton-translocating NADH-quinone oxidoreductase subunit L | 153 | 45 | 264 | 18 | 6.22E-05 |  |
| Fisuc_2138* | NADH/ubiquinone/plastoquinone | 190 | 22 | 316 | 20 | 6.15E-05 |  |
| Fisuc_2142 | GLUG domain-containing protein | 4 | 2 | 11 | 3 | 1.13E-43 |  |
| Fisuc_2143 | Na/Pi-cotransporter II-like protein | 93 | 6 | 229 | 21 | 9.47E-33 |  |
| Fisuc_2145 | GLUG domain-containing protein | 27 | 2 | 38 | 1 | 1.66E-03 |  |
| Fisuc_2146* | type III restriction protein res subunit | 52 | 1 | 167 | 10 | 2.62E-60 |  |
| Fisuc_2148 | hypothetical protein | 146 | 52 | 33 | 2 | 7.34E-270 |  |
| Fisuc_2149 | rhomboid family protein | 30 | 12 | 23 | 1 | 3.96E-07 |  |
| Fisuc_2150 | hypothetical protein | 39 | 5 | 55 | 2 | 5.15E-04 |  |
| Fisuc_2152 | outer membrane assembly lipoprotein YfiO | 278 | 1 | 352 | 7 | 9.27E-03 |  |
| Fisuc_2153 | YjgP/YjgQ family permease | 155 | 10 | 106 | 7 | 1.19E-07 |  |
| Fisuc_2154 | YjgP/YjgQ family permease | 116 | 18 | 88 | 6 | 3.35E-03 |  |
| Fisuc_2155 | diguanylate cyclase | 153 | 22 | 107 | 4 | 9.26E-07 |  |
| Fisuc_2157 | hypothetical protein | 72 | 8 | 57 | 4 | 8.40E-04 |  |
| Fisuc_2158 | hypothetical protein | 175 | 7 | 235 | 26 | 4.96E-03 |  |
| Fisuc_2162 | anaerobic ribonucleoside-triphosphate reductase activating protein | 324 | 11 | 499 | 63 | 6.25E-06 |  |
| Fisuc_2163 | hypothetical protein | 30 | 2 | 10 | 1 | 1.84E-76 |  |
| Fisuc_2164 | hypothetical protein | 155 | 16 | 107 | 13 | 5.14E-05 |  |
| Fisuc_2166 | hypothetical protein | 194 | 58 | 128 | 7 | 8.01E-13 |  |
| Fisuc_2167 | G-D-S-L family lipolytic protein | 142 | 46 | 97 | 4 | 3.92E-05 |  |
| Fisuc_2168 | hypothetical protein | 49 | 21 | 10 | 2 | 1.35E-289 |  |
| Fisuc_2169 | hypothetical protein | 93 | 31 | 52 | 6 | 1.71E-11 |  |
| Fisuc_2170* | helicase, RecD/TraA family | 87 | 5 | 132 | 8 | 2.68E-05 |  |
| Fisuc_2171 | carbon starvation protein CstA | 177 | 7 | 284 | 13 | 7.30E-05 |  |
| Fisuc_2172 | hypothetical protein | 113 | 11 | 240 | 5 | 3.20E-17 |  |
| Fisuc_2174 | metallophosphoesterase | 52 | 11 | 96 | 11 | 9.86E-15 |  |
| Fisuc_2175* | RNA-binding S4 domain-containing protein | 76 | 4 | 117 | 7 | 3.52E-07 |  |
| Fisuc_2176 | Ppx/GppA phosphatase | 105 | 10 | 157 | 6 | 3.94E-05 |  |
| Fisuc_2177 | hypothetical protein | 93 | 8 | 169 | 2 | 5.84E-12 |  |
| Fisuc_2178 | histone | 727 | 195 | 89 | 10 | 0 |  |
| Fisuc_2184* | protein serine/threonine phosphatase | 145 | 16 | 61 | 7 | 9.48E-36 |  |
| Fisuc_2185 | hypothetical protein | 80 | 11 | 34 | 7 | 1.40E-55 |  |
| Fisuc_2186 | hypothetical protein | 42 | 6 | 23 | 1 | 5.63E-17 |  |
| Fisuc_2191 | serine/threonine protein kinase | 37 | 5 | 47 | 1 | 8.28E-03 |  |
| Fisuc_2194* | sigma-54 interacting domain-containing protein | 35 | 1 | 23 | 2 | 3.25E-09 |  |
| Fisuc_2195 | hypothetical protein | 5 | 1 | 3 | 1 | 4.60E-06 |  |
| Fisuc_2196 | hypothetical protein | 20 | 1 | 58 | 6 | 1.73E-54 |  |
| Fisuc_2197 | hypothetical protein | 18 | 2 | 35 | 5 | 2.70E-11 |  |
| Fisuc_2198 | flavin reductase domain-containing FMN-binding protein | 63 | 6 | 81 | 7 | 8.98E-03 |  |
| Fisuc_2200 | hypothetical protein | 81 | 10 | 56 | 6 | 1.18E-06 |  |
| Fisuc_2201 | endo-1,4-beta-xylanase | 159 | 23 | 17 | 4 | 0 | YES |
| Fisuc_2202* | Tex-like protein | 84 | 13 | 140 | 8 | 6.52E-10 |  |
| Fisuc_2204 | acetylglutamate kinase | 612 | 30 | 1065 | 82 | 2.87E-06 |  |
| Fisuc_2205 | acetylornithine and succinylornithine aminotransferase | 408 | 41 | 647 | 45 | 2.60E-04 |  |
| Fisuc_2206 | diguanylate cyclase | 61 | 8 | 28 | 4 | 4.12E-39 |  |
| Fisuc_2207* | integrase family protein | 16 | 4 | 14 | 2 | 1.01E-04 |  |
| Fisuc_2209* | radical SAM protein | 381 | 33 | 336 | 23 | 5.86E-05 |  |
| Fisuc_2212 | hypothetical protein | 54 | 9 | 39 | 5 | 3.86E-06 |  |
| Fisuc_2213 | hypothetical protein | 53 | 6 | 39 | 3 | 1.95E-07 |  |
| Fisuc_2214 | hypothetical protein | 83 | 14 | 57 | 6 | 5.17E-07 |  |
| Fisuc_2215 | hypothetical protein | 83 | 13 | 53 | 1 | 4.39E-09 |  |
| Fisuc_2216 | hypothetical protein | 97 | 8 | 75 | 4 | 4.52E-05 |  |
| Fisuc_2219 | hypothetical protein | 130 | 5 | 209 | 22 | 1.33E-05 |  |
| Fisuc_2220 | mammalian cell entry domain-containing protein | 141 | 11 | 227 | 11 | 7.33E-07 |  |
| Fisuc_2221 | ABC transporter | 118 | 12 | 192 | 9 | 8.13E-06 |  |
| Fisuc_2222 | hypothetical protein | 120 | 6 | 224 | 13 | 1.27E-11 |  |
| Fisuc_2223 | hypothetical protein | 27 | 4 | 23 | 1 | 1.00E-06 |  |
| Fisuc_2224 | hypothetical protein | 24 | 4 | 17 | 1 | 7.39E-11 |  |
| Fisuc_2229 | hypothetical protein | 71 | 3 | 93 | 3 | 3.52E-03 |  |
| Fisuc_2230 | carbohydrate-binding protein | 94 | 23 | 1 | 0 | 0 | YES |
| Fisuc_2239 | hypothetical protein | 353 | 10 | 153 | 11 | 1.51E-28 |  |
| Fisuc_2248* | glycerol-3-phosphate dehydrogenase (NAD(P)(+)) | 515 | 12 | 506 | 46 | 4.64E-03 |  |
| Fisuc_2249 | hypothetical protein | 3096 | 125 | 453 | 148 | 0 |  |
| Fisuc_2251 | exopolysaccharide biosynthesis polyprenyl glycosylphosphotransferase | 136 | 7 | 214 | 8 | 5.28E-06 |  |
| Fisuc_2252 | hypothetical protein | 60 | 13 | 18 | 3 | 1.75E-93 |  |
| Fisuc_2254 | exopolysaccharide biosynthesis polyprenyl glycosylphosphotransferase | 214 | 29 | 322 | 20 | 8.03E-03 |  |
| Fisuc_2255 | nicotinate-nucleotide pyrophosphorylase | 107 | 8 | 185 | 11 | 3.16E-08 |  |
| Fisuc_2256 | NAD-dependent epimerase/dehydratase | 582 | 37 | 489 | 32 | 1.55E-04 |  |
| Fisuc_2257 | hypothetical protein | 60 | 2 | 36 | 5 | 3.45E-14 |  |
| Fisuc_2260 | hypothetical protein | 44 | 1 | 60 | 7 | 1.39E-03 |  |
| Fisuc_2263 | hypothetical protein | 132 | 16 | 52 | 1 | 1.89E-58 |  |
| Fisuc_2267 | metallophosphoesterase | 123 | 8 | 78 | 8 | 1.51E-11 |  |
| Fisuc_2268 | hypothetical protein | 2458 | 133 | 1715 | 137 | 6.06E-07 |  |
| Fisuc_2270 | hypothetical protein | 35 | 10 | 24 | 1 | 9.45E-12 |  |
| Fisuc_2271 | hypothetical protein | 433 | 25 | 105 | 4 | 4.19E-139 |  |
| Fisuc_2272 | hypothetical protein | 435 | 29 | 135 | 13 | 1.13E-82 |  |
| Fisuc_2277 | ATPase AAA | 138 | 29 | 420 | 122 | 1.79E-37 |  |
| Fisuc_2279 | histidinol-phosphate aminotransferase | 157 | 8 | 264 | 6 | 3.59E-09 |  |
| Fisuc_2280 | histidinol-phosphate phosphatase | 151 | 4 | 227 | 1 | 3.47E-05 |  |
| Fisuc_2281 | DNA internalization-related competence protein ComEC/Rec2 | 11 | 1 | 10 | 1 | 4.06E-03 |  |
| Fisuc_2282 | hypothetical protein | 55 | 9 | 34 | 4 | 2.95E-10 |  |
| Fisuc_2283 | GTP-binding protein | 127 | 3 | 96 | 6 | 2.38E-05 |  |
| Fisuc_2284 | hypothetical protein | 363 | 23 | 212 | 24 | 8.94E-17 |  |
| Fisuc_2285 | hypothetical protein | 146 | 41 | 114 | 9 | 1.77E-04 |  |
| Fisuc_2294 | hypothetical protein | 1113 | 69 | 882 | 14 | 1.03E-03 |  |
| Fisuc_2295* | NusA antitermination factor | 992 | 60 | 882 | 6 | 7.96E-04 |  |
| Fisuc_2302* | SNF2-related protein | 22 | 2 | 37 | 2 | 4.86E-05 |  |
| Fisuc_2303 | glycoside hydrolase family protein | 53 | 9 | 22 | 2 | 6.99E-42 | YES |
| Fisuc_2304 | hypothetical protein | 168 | 12 | 107 | 9 | 3.45E-12 |  |
| Fisuc_2305 | 7-cyano-7-deazaguanine reductase | 179 | 11 | 256 | 20 | 2.76E-03 |  |
| Fisuc_2306 | hypothetical protein | 95 | 0 | 162 | 9 | 1.46E-08 |  |
| Fisuc_2308 | hypothetical protein | 1320 | 78 | 1085 | 64 | 2.53E-05 |  |
| Fisuc_2310 | family 2 glycosyl transferase | 67 | 11 | 87 | 7 | 4.09E-03 | YES |
| Fisuc_2311 | ATPase AAA | 70 | 4 | 48 | 6 | 9.06E-07 |  |
| Fisuc_2312 | hypothetical protein | 36 | 5 | 24 | 3 | 1.29E-11 |  |
| Fisuc_2313 | transglutaminase domain protein | 30 | 2 | 14 | 1 | 3.09E-41 |  |
| Fisuc_2314 | hypothetical protein | 482 | 28 | 250 | 19 | 2.13E-26 |  |
| Fisuc_2315 | hypothetical protein | 65 | 3 | 11 | 2 | 0 | YES |
| Fisuc_2317 | endo-1,4-beta-glucanase/xyloglucanase, , gly74A | 42 | 6 | 1 | 0 | 0 | YES |
| Fisuc_2318 | hypothetical protein | 288 | 51 | 100 | 11 | 4.32E-54 |  |
| Fisuc_2319 | hypothetical protein | 44 | 2 | 64 | 5 | 1.09E-04 |  |
| Fisuc_2322 | CDP-alcohol phosphatidyltransferase | 376 | 21 | 251 | 20 | 8.13E-08 |  |
| Fisuc_2324 | tRNA (guanine-N(7)-)-methyltransferase | 179 | 15 | 244 | 14 | 2.28E-03 |  |
| Fisuc_2325 | hypothetical protein | 210 | 13 | 301 | 17 | 9.90E-04 |  |
| Fisuc_2326 | hypothetical protein | 504 | 7 | 447 | 49 | 4.99E-03 |  |
| Fisuc_2327* | exodeoxyribonuclease III Xth | 108 | 9 | 241 | 4 | 1.76E-20 |  |
| Fisuc_2331 | diguanylate cyclase | 225 | 13 | 126 | 12 | 1.53E-15 |  |
| Fisuc_2332 | diguanylate cyclase | 261 | 25 | 140 | 20 | 6.17E-18 |  |
| Fisuc_2335 | prevent-host-death family protein | 57 | 8 | 94 | 4 | 4.84E-06 |  |
| Fisuc_2339* | NADH-ubiquinone oxidoreductase chain 4L | 105 | 13 | 98 | 6 | 8.98E-03 |  |
| Fisuc_2348 | hypothetical protein | 29 | 8 | 9 | 0 | 7.47E-128 | YES |
| Fisuc_2349 | bifunctional phosphoserine phosphatase/homoserine phosphotransferase | 223 | 9 | 308 | 23 | 1.22E-03 |  |
| Fisuc_2351 | DJ-1 family protein | 233 | 43 | 167 | 10 | 1.23E-04 |  |
| Fisuc_2352 | FeS assembly protein SufB | 152 | 5 | 289 | 9 | 3.09E-14 |  |
| Fisuc_2355 | hypothetical protein | 72 | 15 | 60 | 11 | 4.10E-05 |  |
| Fisuc_2356 | hypothetical protein | 97 | 7 | 71 | 3 | 2.39E-09 |  |
| Fisuc_2357 | hypothetical protein | 134 | 13 | 90 | 3 | 1.46E-07 |  |
| Fisuc_2360 | hypothetical protein | 68 | 3 | 142 | 8 | 2.64E-17 |  |
| Fisuc_2361 | class I and II aminotransferase | 82 | 9 | 125 | 8 | 1.74E-05 |  |
| Fisuc_2362 | glycoside hydrolase family protein | 292 | 85 | 53 | 1 | 9.54E-265 | YES |
| Fisuc_2363 | Pectate lyase/Amb allergen | 211 | 40 | 20 | 2 | 0 | YES |
| Fisuc_2364 | cellulase | 583 | 134 | 43 | 6 | 0 | YES |
| Fisuc_2365* | queuine tRNA-ribosyltransferase | 111 | 4 | 218 | 8 | 5.17E-14 |  |
| Fisuc_2367 | preprotein translocase subunit SecG | 1099 | 193 | 2062 | 5 | 3.64E-05 |  |
| Fisuc_2368 | triosephosphate isomerase | 1201 | 222 | 2382 | 45 | 7.19E-06 |  |
| Fisuc_2369 | hypothetical protein | 60 | 16 | 19 | 4 | 1.32E-79 |  |
| Fisuc_2370 | hypothetical protein | 2032 | 167 | 1036 | 30 | 2.81E-24 |  |
| Fisuc_2373* | recA protein | 167 | 11 | 377 | 55 | 6.38E-25 |  |
| Fisuc_2374 | CinA domain-containing protein | 109 | 12 | 528 | 74 | 4.70E-210 |  |
| Fisuc_2378 | cadherin | 720 | 122 | 313 | 21 | 6.31E-08 |  |
| Fisuc_2380 | hypothetical protein | 199 | 19 | 133 | 11 | 1.09E-07 |  |
| Fisuc_2381 | hypothetical protein | 201 | 18 | 134 | 8 | 4.78E-06 |  |
| Fisuc_2386 | integral membrane sensor hybrid histidine kinase | 193 | 19 | 119 | 12 | 4.08E-16 |  |
| Fisuc_2387* | 30S ribosomal protein S15 | 2685 | 24 | 4550 | 73 | 1.04E-03 |  |
| Fisuc_2389* | metal dependent phosphohydrolase | 387 | 44 | 171 | 7 | 1.85E-29 |  |
| Fisuc_2390* | metal dependent phosphohydrolase | 236 | 41 | 111 | 4 | 7.12E-26 |  |
| Fisuc_2391 | 1-hydroxy-2-methyl-2-(E)-butenyl 4-diphosphate synthase | 117 | 31 | 193 | 18 | 1.27E-09 |  |
| Fisuc_2392 | D-isomer specific 2-hydroxyacid dehydrogenase NAD-binding protein | 664 | 34 | 1134 | 13 | 1.48E-04 |  |
| Fisuc_2393 | AMP-dependent synthetase and ligase | 45 | 8 | 27 | 1 | 7.44E-12 |  |
| Fisuc_2394 | hypothetical protein | 50 | 4 | 34 | 5 | 7.29E-15 |  |
| Fisuc_2396 | carbohydrate-binding CenC domain protein | 8 | 2 | 3 | 1 | 3.52E-34 | YES |
| Fisuc_2397* | radical SAM protein | 40 | 6 | 63 | 4 | 2.33E-09 |  |
| Fisuc_2402 | CarD family transcriptional regulator | 138 | 14 | 199 | 8 | 2.74E-04 |  |
| Fisuc_2403* | 50S ribosomal protein L19 | 2219 | 237 | 4873 | 386 | 2.91E-10 |  |
| Fisuc_2404* | tRNA (guanine-N1)-methyltransferase | 263 | 18 | 639 | 28 | 1.78E-29 |  |
| Fisuc_2405* | 16S rRNA processing protein RimM | 342 | 28 | 763 | 23 | 5.22E-23 |  |
| Fisuc_2406* | 30S ribosomal protein S16 | 1300 | 94 | 2849 | 105 | 1.73E-09 |  |
| Fisuc_2407 | diguanylate cyclase | 72 | 9 | 23 | 6 | 2.21E-104 |  |
| Fisuc_2408 | hypothetical protein | 112 | 14 | 41 | 3 | 1.84E-82 |  |
| Fisuc_2409 | N-acetyltransferase GCN5 | 157 | 23 | 59 | 6 | 6.73E-62 |  |
| Fisuc_2413 | hypothetical protein | 451 | 32 | 187 | 42 | 1.26E-37 |  |
| Fisuc_2414 | hypothetical protein | 411 | 12 | 138 | 22 | 2.93E-108 |  |
| Fisuc_2415 | hypothetical protein | 1545 | 196 | 366 | 18 | 8.84E-177 |  |
| Fisuc_2416 | hypothetical protein | 169 | 8 | 64 | 6 | 6.79E-49 |  |
| Fisuc_2417 | hypothetical protein | 40 | 4 | 11 | 2 | 2.45E-173 |  |
| Fisuc_2418 | amino acid carrier protein | 60 | 7 | 43 | 3 | 7.95E-07 |  |
| Fisuc_2419 | class I and II aminotransferase | 56 | 10 | 76 | 8 | 4.83E-03 |  |
| Fisuc_2420 | alpha/beta hydrolase fold protein | 9 | 4 | 46 | 11 | 1.36E-195 |  |
| Fisuc_2421 | hypothetical protein | 5 | 1 | 15 | 2 | 1.37E-43 |  |
| Fisuc_2422 | hypothetical protein | 7 | 2 | 13 | 2 | 4.16E-13 |  |
| Fisuc_2424 | glycoside hydrolase family protein | 117 | 29 | 52 | 4 | 1.79E-30 | YES |
| Fisuc_2429 | 2-amino-4-hydroxy-6-hydroxymethyldihydropteridine pyrophosphokinase | 302 | 22 | 403 | 13 | 5.81E-03 |  |
| Fisuc_2431 | pantoate/beta-alanine ligase | 293 | 16 | 412 | 22 | 1.31E-04 |  |
| Fisuc_2432 | adenylate/guanylate cyclase with Chase sensor | 82 | 7 | 62 | 4 | 2.44E-04 |  |
| Fisuc_2434 | AMP-dependent synthetase and ligase | 49 | 5 | 41 | 3 | 9.01E-03 |  |
| Fisuc_2435 | anti-sigma-factor antagonist | 51 | 4 | 47 | 2 | 2.54E-04 |  |
| Fisuc_2437* | protein serine/threonine phosphatase | 35 | 3 | 27 | 1 | 6.23E-03 |  |
| Fisuc_2439* | ribosomal 5S rRNA E-loop binding protein Ctc/L25/TL5 | 2703 | 115 | 5999 | 76 | 1.93E-03 |  |
| Fisuc_2440 | hypothetical protein | 139 | 18 | 235 | 1 | 9.22E-09 |  |
| Fisuc_2441* | peptidyl-tRNA hydrolase | 179 | 20 | 256 | 13 | 6.69E-03 |  |
| Fisuc_2443 | hypothetical protein | 84 | 20 | 65 | 7 | 1.27E-03 |  |
| Fisuc_2444 | hypothetical protein | 53 | 7 | 27 | 4 | 1.74E-39 |  |
| Fisuc_2445 | hypothetical protein | 81 | 20 | 34 | 1 | 8.87E-64 |  |
| Fisuc_2446 | hypothetical protein | 115 | 14 | 53 | 3 | 1.47E-30 |  |
| Fisuc_2447 | transcriptional regulator, XRE family | 110 | 9 | 56 | 1 | 6.32E-32 |  |
| Fisuc_2448 | type II restriction enzyme (methylase subunit) | 8 | 1 | 7 | 1 | 7.63E-04 |  |
| Fisuc_2451 | hypothetical protein | 80 | 11 | 52 | 3 | 1.21E-09 |  |
| Fisuc_2452 | inosine-5'-monophosphate dehydrogenase | 354 | 53 | 534 | 49 | 4.60E-03 |  |
| Fisuc_2455* | glutamyl-tRNA(Gln) amidotransferase subunit A | 190 | 31 | 341 | 21 | 2.18E-07 |  |
| Fisuc_2456 | hypothetical protein | 193 | 15 | 342 | 12 | 4.31E-11 |  |
| Fisuc_2459 | hypothetical protein | 68 | 7 | 62 | 8 | 6.13E-03 |  |
| Fisuc_2461 | arabinogalactan endo-1,4-beta-galactosidase | 22 | 3 | 9 | 1 | 1.28E-72 | YES |
| Fisuc_2463 | hypothetical protein | 69 | 8 | 49 | 3 | 3.07E-08 |  |
| Fisuc_2468 | hypothetical protein | 28 | 8 | 25 | 3 | 8.62E-04 |  |
| Fisuc_2469 | phage-associated protein-like protein | 25 | 4 | 21 | 1 | 4.85E-05 |  |
| Fisuc_2470 | hypothetical protein | 52 | 3 | 66 | 2 | 7.55E-03 |  |
| Fisuc_2474 | hypothetical protein | 204 | 55 | 60 | 9 | 9.61E-95 |  |
| Fisuc_2477 | carbohydrate binding family 6 | 101 | 21 | 13 | 2 | 0 | YES |
| Fisuc_2478 | carbohydrate binding family 6 | 31 | 12 | 12 | 1 | 4.21E-70 | YES |
| Fisuc_2479 | G-D-S-L family lipolytic protein | 23 | 10 | 6 | 1 | 8.20E-176 | YES |
| Fisuc_2480 | hypothetical protein | 106 | 15 | 45 | 1 | 1.54E-35 |  |
| Fisuc_2481 | Pectate lyase/Amb allergen | 63 | 10 | 32 | 4 | 6.65E-24 | YES |
| Fisuc_2483 | hypothetical protein | 24 | 1 | 61 | 3 | 4.17E-36 |  |
| Fisuc_2484 | fibro-slime family protein | 27 | 25 | 2 | 0 | 0 |  |
| Fisuc_2485 | carbohydrate binding family 6 | 7 | 2 | 1 | 1 | 1.87E-177 | YES |
| Fisuc_2488 | surface antigen (D15) | 82 | 6 | 69 | 6 | 1.64E-03 |  |
| Fisuc_2498 | hypothetical protein | 52 | 8 | 84 | 23 | 1.24E-07 |  |
| Fisuc_2499 | hypothetical protein | 63 | 3 | 102 | 20 | 3.67E-07 |  |
| Fisuc_2501* | type III restriction protein res subunit | 4 | 1 | 17 | 7 | 1.08E-134 |  |
| Fisuc_2502* | succinate dehydrogenase/fumarate reductase iron-sulfur subunit | 238 | 19 | 332 | 29 | 3.74E-03 |  |
| Fisuc_2506 | hypothetical protein | 156 | 12 | 228 | 16 | 8.83E-05 |  |
| Fisuc_2507 | hypothetical protein | 32 | 7 | 53 | 6 | 1.09E-08 |  |
| Fisuc_2510 | hypothetical protein | 69 | 3 | 114 | 5 | 1.35E-05 |  |
| Fisuc_2511 | thymidylate synthase | 339 | 24 | 295 | 20 | 3.11E-03 |  |
| Fisuc_2517 | hypothetical protein | 76 | 1 | 61 | 4 | 1.32E-03 |  |
| Fisuc_2519 | lipoprotein | 14 | 3 | 10 | 1 | 5.01E-11 |  |
| Fisuc_2520 | hypothetical protein | 127 | 24 | 42 | 10 | 6.51E-106 |  |
| Fisuc_2521 | lipoprotein | 31 | 6 | 14 | 1 | 5.12E-32 |  |
| Fisuc_2522 | ATPase AAA | 28 | 7 | 6 | 1 | 1.13E-254 |  |
| Fisuc_2523 | lipoprotein | 55 | 14 | 22 | 3 | 1.06E-65 |  |
| Fisuc_2524 | PDZ/DHR/GLGF domain-containing protein | 1026 | 47 | 642 | 17 | 5.76E-17 |  |
| Fisuc_2530 | hypothetical protein | 9 | 1 | 18 | 2 | 7.40E-18 |  |
| Fisuc_2531 | hypothetical protein | 20 | 1 | 26 | 0 | 5.18E-03 |  |
| Fisuc_2534 | carbohydrate binding family 6 | 116 | 34 | 46 | 4 | 1.34E-39 | YES |
| Fisuc_2535 | hypothetical protein | 359 | 51 | 260 | 15 | 8.53E-08 |  |
| Fisuc_2536 | hypothetical protein | 688 | 68 | 543 | 6 | 1.52E-05 |  |
| Fisuc_2538 | type II secretion system protein E | 269 | 34 | 192 | 9 | 5.63E-05 |  |
| Fisuc_2539* | UvrD/REP helicase | 62 | 3 | 115 | 6 | 3.75E-13 |  |
| Fisuc_2540* | glutamyl-tRNA(Gln) amidotransferase subunit C | 209 | 10 | 449 | 27 | 6.37E-22 |  |
| Fisuc_2541 | 3-deoxy-manno-octulosonate cytidylyltransferase | 64 | 16 | 114 | 17 | 1.22E-13 |  |
| Fisuc_2543 | hypothetical protein | 634 | 48 | 448 | 35 | 6.19E-07 |  |
| Fisuc_2544 | hypothetical protein | 523 | 34 | 353 | 14 | 9.09E-06 |  |
| Fisuc_2545* | methyltransferase | 258 | 9 | 185 | 10 | 2.65E-04 |  |
| Fisuc_2548 | PfkB domain-containing protein | 201 | 32 | 328 | 17 | 9.22E-09 |  |
| Fisuc_2549 | hypothetical protein | 189 | 43 | 739 | 82 | 1.04E-105 |  |
| Fisuc_2552 | hypothetical protein | 469 | 40 | 721 | 45 | 3.28E-07 |  |
| Fisuc_2554* | inorganic diphosphatase | 477 | 41 | 1195 | 49 | 3.03E-24 |  |
| Fisuc_2555 | hypothetical protein | 705 | 39 | 1391 | 114 | 1.73E-09 |  |
| Fisuc_2556 | UDP-glucose 4-epimerase | 258 | 4 | 395 | 14 | 6.01E-05 |  |
| Fisuc_2557 | mechanosensitive ion channel MscS | 59 | 6 | 40 | 5 | 1.06E-07 |  |
| Fisuc_2562 | hypothetical protein | 156 | 24 | 111 | 7 | 3.94E-06 |  |
| Fisuc_2565 | Pectate lyase/Amb allergen | 22 | 5 | 8 | 1 | 6.47E-84 | YES |
| Fisuc_2567* | CMP/dCMP deaminase zinc-binding protein | 54 | 9 | 24 | 1 | 8.80E-58 |  |
| Fisuc_2569* | dihydrouridine synthase DuS | 50 | 4 | 70 | 1 | 5.14E-05 |  |
| Fisuc_2571 | TM2 domain-containing protein | 139 | 10 | 195 | 6 | 1.26E-03 |  |
| Fisuc_2574 | hypothetical protein | 63 | 15 | 44 | 3 | 6.82E-07 |  |
| Fisuc_2575 | hypothetical protein | 46 | 2 | 29 | 3 | 3.37E-09 |  |
| Fisuc_2576 | hypothetical protein | 119 | 21 | 40 | 11 | 1.24E-61 |  |
| Fisuc_2578 | hypothetical protein | 89 | 8 | 78 | 5 | 9.14E-03 |  |
| Fisuc_2579 | glycoside hydrolase family protein | 339 | 18 | 218 | 7 | 1.04E-06 | YES |
| Fisuc_2582 | hypothetical protein | 119 | 12 | 43 | 4 | 9.20E-54 |  |
| Fisuc_2583 | hypothetical protein | 9 | 1 | 9 | 1 | 4.77E-04 |  |
| Fisuc_2585 | hypothetical protein | 25 | 4 | 18 | 0 | 3.43E-09 |  |
| Fisuc_2586 | hypothetical protein | 43 | 5 | 20 | 2 | 7.32E-46 |  |
| Fisuc_2587 | hypothetical protein | 10 | 3 | 5 | 0 | 8.12E-25 |  |
| Fisuc_2593 | hypothetical protein | 36 | 2 | 74 | 13 | 3.78E-14 |  |
| Fisuc_2594 | hypothetical protein | 38 | 3 | 109 | 7 | 1.06E-56 |  |
| Fisuc_2598 | hypothetical protein | 73 | 9 | 113 | 14 | 4.56E-07 |  |
| Fisuc_2599* | metal dependent phosphohydrolase | 103 | 21 | 72 | 9 | 1.88E-05 |  |
| Fisuc_2600 | hypothetical protein | 20 | 2 | 10 | 1 | 2.14E-27 |  |
| Fisuc_2601 | hypothetical protein | 11 | 3 | 4 | 1 | 9.95E-60 |  |
| Fisuc_2602 | hypothetical protein | 2 | 1 | 1 | 1 | 2.45E-31 |  |
| Fisuc_2603 | hypothetical protein | 4 | 1 | 3 | 1 | 6.34E-10 |  |
| Fisuc_2604 | hypothetical protein | 4 | 1 | 2 | 1 | 7.07E-14 |  |
| Fisuc_2605 | hypothetical protein | 8 | 2 | 7 | 1 | 9.02E-03 |  |
| Fisuc_2606 | hypothetical protein | 11 | 2 | 9 | 0 | 3.74E-05 |  |
| Fisuc_2607* | Sua5/YciO/YrdC/YwlC family protein | 83 | 7 | 125 | 8 | 9.50E-07 |  |
| Fisuc_2609 | peptidase M23 | 207 | 18 | 304 | 13 | 4.06E-06 |  |
| Fisuc_2610 | hypothetical protein | 42 | 8 | 30 | 0 | 7.99E-12 |  |
| Fisuc_2614* | histidyl-tRNA synthetase | 182 | 16 | 239 | 9 | 1.25E-03 |  |
| Fisuc_2615* | malate dehydrogenase | 1043 | 114 | 2137 | 79 | 1.92E-04 |  |
| Fisuc_2616 | hypothetical protein | 74 | 14 | 9 | 1 | 0 |  |
| Fisuc_2617 | hypothetical protein | 68 | 13 | 44 | 6 | 2.99E-08 |  |
| Fisuc_2618 | hypothetical protein | 94 | 20 | 58 | 14 | 2.05E-09 |  |
| Fisuc_2619 | hypothetical protein | 70 | 13 | 44 | 5 | 2.29E-09 |  |
| Fisuc_2620 | hypothetical protein | 98 | 9 | 65 | 5 | 1.23E-06 |  |
| Fisuc_2621 | glycoside hydrolase family protein | 13 | 3 | 2 | 1 | 0 | YES |
| Fisuc_2622 | glycoside hydrolase family protein | 13 | 3 | 5 | 1 | 5.29E-86 | YES |
| Fisuc_2623 | glycoside hydrolase family protein | 3 | 1 | 1 | 0 | 1.16E-24 | YES |
| Fisuc_2626 | hypothetical protein | 119 | 15 | 158 | 8 | 6.65E-03 |  |
| Fisuc_2630 | hypothetical protein | 15 | 3 | 6 | 2 | 2.86E-30 |  |
| Fisuc_2635* | lysyl-tRNA synthetase | 146 | 8 | 219 | 5 | 1.43E-06 |  |
| Fisuc_2636 | S23 ribosomal protein | 75 | 14 | 151 | 13 | 8.79E-17 |  |
| Fisuc_2637 | hypothetical protein | 29 | 6 | 46 | 3 | 2.31E-07 |  |
| Fisuc_2640 | adenylate kinase | 1321 | 97 | 2131 | 52 | 2.84E-03 |  |
| Fisuc_2644* | 3-isopropylmalate dehydrogenase | 264 | 46 | 455 | 41 | 2.58E-05 |  |
| Fisuc_2652 | group 1 glycosyl transferase | 513 | 13 | 840 | 5 | 8.28E-03 | YES |
| Fisuc_2653 | group 1 glycosyl transferase | 378 | 27 | 604 | 11 | 1.73E-03 | YES |
| Fisuc_2654 | hypothetical protein | 241 | 15 | 346 | 9 | 7.55E-03 |  |
| Fisuc_2656 | family 2 glycosyl transferase | 348 | 34 | 566 | 32 | 2.33E-03 | YES |
| Fisuc_2658* | 4Fe-4S ferredoxin | 283 | 16 | 444 | 10 | 6.23E-06 |  |
| Fisuc_2662* | Fe-S type, tartrate/fumarate subfamily hydro-lyase subunit alpha | 31 | 8 | 47 | 6 | 9.42E-06 |  |
| Fisuc_2663 | hypothetical protein | 33 | 6 | 47 | 1 | 1.82E-04 |  |
| Fisuc_2664* | Fe-S type hydro-lyase tartrate/fumarate beta region | 61 | 5 | 89 | 5 | 8.44E-05 |  |
| Fisuc_2665 | MmgE/PrpD family protein | 36 | 8 | 64 | 8 | 7.77E-13 |  |
| Fisuc_2666 | capsule synthesis protein CapA | 51 | 8 | 78 | 5 | 1.39E-07 |  |
| Fisuc_2667 | hypothetical protein | 83 | 6 | 137 | 10 | 4.12E-06 |  |
| Fisuc_2668 | hypothetical protein | 206 | 12 | 336 | 20 | 5.37E-09 |  |
| Fisuc_2669 | phosphoribosylglycinamide synthetase | 213 | 18 | 413 | 15 | 3.66E-10 |  |
| Fisuc_2670 | peptidase | 183 | 49 | 389 | 29 | 4.04E-23 |  |
| Fisuc_2671 | DegT/DnrJ/EryC1/StrS aminotransferase | 166 | 16 | 360 | 12 | 1.17E-22 |  |
| Fisuc_2673 | hypothetical protein | 20 | 5 | 14 | 2 | 9.40E-09 |  |
| Fisuc_2674 | hypothetical protein | 14 | 3 | 10 | 1 | 3.01E-11 | YES |
| Fisuc_2676 | group 1 glycosyl transferase | 9 | 1 | 7 | 1 | 4.87E-03 | YES |
| Fisuc_2677 | hypothetical protein | 7 | 2 | 5 | 0 | 1.90E-04 |  |
| Fisuc_2681 | UDP-N-acetylglucosamine 2-epimerase | 9 | 2 | 16 | 2 | 2.49E-07 |  |
| Fisuc_2682 | dTDP-4-dehydrorhamnose reductase | 7 | 1 | 12 | 1 | 4.46E-07 |  |
| Fisuc_2683 | UDP-glucose 4-epimerase | 9 | 1 | 15 | 2 | 5.71E-09 |  |
| Fisuc_2684 | group 1 glycosyl transferase | 5 | 2 | 12 | 1 | 3.87E-26 | YES |
| Fisuc_2685 | NAD-dependent epimerase/dehydratase | 6 | 1 | 9 | 1 | 2.23E-05 |  |
| Fisuc_2687 | PglB protein | 5 | 1 | 7 | 1 | 7.15E-03 |  |
| Fisuc_2688 | hypothetical protein | 33 | 3 | 51 | 1 | 5.43E-04 |  |
| Fisuc_2690 | hypothetical protein | 37 | 6 | 26 | 1 | 3.53E-05 |  |
| Fisuc_2693 | UDP-glucose 4-epimerase | 186 | 11 | 288 | 12 | 1.85E-06 |  |
| Fisuc_2696 | hypothetical protein | 0 | 0 | 1 | 1 | 5.10E-04 |  |
| Fisuc_2698 | hypothetical protein | 569 | 41 | 403 | 55 | 9.20E-07 |  |
| Fisuc_2699 | hypothetical protein | 250 | 17 | 157 | 33 | 1.55E-15 |  |
| Fisuc_2700 | hypothetical protein | 175 | 26 | 140 | 6 | 1.07E-03 |  |
| Fisuc_2701 | hypothetical protein | 98 | 19 | 78 | 3 | 4.55E-05 |  |
| Fisuc_2702 | hypothetical protein | 44 | 10 | 39 | 6 | 5.99E-03 |  |
| Fisuc_2705 | hypothetical protein | 241 | 26 | 95 | 4 | 3.78E-72 |  |
| Fisuc_2706 | phosphoribosylglycinamide formyltransferase | 323 | 12 | 465 | 14 | 6.90E-04 |  |
| Fisuc_2707* | Ribonuclease H | 185 | 11 | 252 | 15 | 2.09E-03 |  |
| Fisuc_2709* | peptide chain release factor 3 | 45 | 8 | 112 | 6 | 3.07E-29 |  |
| Fisuc_2710 | hypothetical protein | 482 | 33 | 369 | 8 | 7.91E-05 |  |
| Fisuc_2711 | radical SAM protein | 132 | 4 | 69 | 4 | 2.10E-18 |  |
| Fisuc_2712 | proteophosphoglycan ppg4 | 118 | 5 | 52 | 2 | 1.02E-45 |  |
| Fisuc_2713 | hypothetical protein | 186 | 12 | 91 | 4 | 2.55E-26 |  |
| Fisuc_2714 | acetolactate synthase small subunit | 404 | 35 | 311 | 47 | 1.83E-04 |  |
| Fisuc_2715 | acetolactate synthase large subunit | 447 | 32 | 342 | 79 | 3.25E-09 |  |
| Fisuc_2716 | hypothetical protein | 383 | 28 | 326 | 3 | 3.96E-03 |  |
| Fisuc_2722 | hypothetical protein | 41 | 9 | 31 | 2 | 1.43E-05 |  |
| Fisuc_2724 | hypothetical protein | 76 | 9 | 60 | 4 | 7.63E-06 |  |
| Fisuc_2725 | hypothetical protein | 88 | 17 | 68 | 3 | 2.45E-04 |  |
| Fisuc_2728 | hypothetical protein | 30 | 7 | 25 | 2 | 3.06E-06 |  |
| Fisuc_2730 | 1-deoxy-D-xylulose-5-phosphate synthase | 73 | 19 | 56 | 3 | 5.60E-03 |  |
| Fisuc_2731 | Lysophospholipase-like protein | 190 | 7 | 128 | 11 | 4.74E-09 |  |
| Fisuc_2733 | riboflavin biosynthesis protein RibD | 208 | 3 | 166 | 4 | 6.05E-04 |  |
| Fisuc_2734 | hypothetical protein | 69 | 4 | 54 | 4 | 5.73E-04 |  |
| Fisuc_2735 | riboflavin synthase subunit alpha | 340 | 22 | 561 | 40 | 2.86E-09 |  |
| Fisuc_2737 | 6,7-dimethyl-8-ribityllumazine synthase | 214 | 23 | 334 | 25 | 1.79E-05 |  |
| Fisuc_2738* | NusB antitermination factor | 236 | 30 | 364 | 16 | 1.59E-05 |  |
| Fisuc_2740 | family 2 glycosyl transferase | 35 | 11 | 89 | 10 | 2.86E-37 | YES |
| Fisuc_2745 | twitching motility protein | 472 | 19 | 464 | 22 | 9.84E-03 |  |
| Fisuc_2746 | hypothetical protein | 61 | 19 | 14 | 1 | 4.16E-216 |  |
| Fisuc_2750* | Chromosomal replication initiator DnaA | 166 | 3 | 322 | 36 | 5.11E-14 |  |
| Fisuc_2751 | NAD-dependent epimerase/dehydratase | 67 | 3 | 151 | 8 | 1.53E-26 |  |
| Fisuc_2752 | hypothetical protein | 469 | 14 | 346 | 18 | 2.67E-08 |  |
| Fisuc_2754 | FKBP-type peptidylprolyl isomerase | 355 | 26 | 583 | 13 | 4.66E-09 |  |
| Fisuc_2755 | hypothetical protein | 366 | 23 | 76 | 11 | 7.88E-221 |  |
| Fisuc_2756 | hypothetical protein | 348 | 3 | 237 | 15 | 1.55E-09 |  |
| Fisuc_2758 | histidinol dehydrogenase | 103 | 27 | 178 | 11 | 3.38E-09 |  |
| Fisuc_2763 | hypothetical protein | 227 | 19 | 324 | 16 | 1.96E-05 |  |
| Fisuc_2764 | OmpA/MotB domain-containing protein | 94 | 5 | 174 | 12 | 4.78E-11 |  |
| Fisuc_2765 | hypothetical protein | 281 | 33 | 214 | 12 | 1.07E-03 |  |
| Fisuc_2766 | hypothetical protein | 245 | 56 | 99 | 5 | 8.34E-46 |  |
| Fisuc_2767 | ADP-ribosylation/Crystallin J1 | 276 | 88 | 103 | 8 | 2.06E-48 |  |
| Fisuc_2768 | lipoprotein | 37 | 5 | 26 | 5 | 8.14E-10 |  |
| Fisuc_2769 | hypothetical protein | 39 | 6 | 29 | 2 | 9.80E-10 |  |
| Fisuc_2770 | ABC transporter | 379 | 17 | 757 | 28 | 3.88E-06 |  |
| Fisuc_2771 | hypothetical protein | 92 | 6 | 57 | 1 | 1.45E-10 |  |
| Fisuc_2772 | polar amino acid ABC transporter inner membrane subunit | 174 | 9 | 133 | 15 | 1.61E-04 |  |
| Fisuc_2773 | ABC transporter | 129 | 5 | 106 | 6 | 3.02E-03 |  |
| Fisuc_2781 | hypothetical protein | 80 | 8 | 74 | 16 | 9.14E-03 |  |
| Fisuc_2783 | hypothetical protein | 49 | 5 | 77 | 18 | 2.98E-06 |  |
| Fisuc_2784 | hypothetical protein | 54 | 4 | 88 | 21 | 3.40E-06 |  |
| Fisuc_2786 | S-adenosylmethionine synthetase | 442 | 39 | 819 | 46 | 3.52E-05 |  |
| Fisuc_2787 | hypothetical protein | 249 | 23 | 107 | 25 | 1.13E-35 |  |
| Fisuc_2788 | peptidase M23 | 234 | 25 | 142 | 14 | 7.03E-14 |  |
| Fisuc_2789 | hypothetical protein | 7 | 2 | 3 | 0 | 2.91E-16 |  |
| Fisuc_2791 | hypothetical protein | 34 | 8 | 67 | 2 | 1.20E-16 |  |
| Fisuc_2792 | hypothetical protein | 886 | 39 | 6342 | 1530 | 0 |  |
| Fisuc_2793 | metal dependent phosphohydrolase | 234 | 28 | 129 | 10 | 1.21E-14 |  |
| Fisuc_2794 | PhoH family protein | 289 | 26 | 164 | 14 | 9.01E-16 |  |
| Fisuc_2795 | hypothetical protein | 1587 | 103 | 1280 | 79 | 2.05E-05 |  |
| Fisuc_2798 | hypothetical protein | 41 | 10 | 54 | 6 | 3.72E-03 |  |
| Fisuc_2799* | avirulence protein | 118 | 32 | 23 | 2 | 1.68E-279 |  |
| Fisuc_2800 | hypothetical protein | 37 | 14 | 6 | 1 | 0 | YES |
| Fisuc_2802 | inositol monophosphatase | 81 | 9 | 170 | 7 | 2.80E-17 |  |
| Fisuc_2803 | carboxyl transferase | 1300 | 171 | 3833 | 235 | 4.68E-07 |  |
| Fisuc_2804 | malonyl CoA-acyl carrier protein transacylase | 1388 | 168 | 4031 | 128 | 3.02E-10 |  |
| Fisuc_2805 | acyl-carrier protein | 852 | 18 | 1900 | 72 | 6.46E-22 |  |
| Fisuc_2806 | beta-ketoacyl synthase | 383 | 39 | 1106 | 55 | 3.34E-15 |  |
| Fisuc_2807 | short-chain dehydrogenase/reductase SDR | 247 | 29 | 524 | 40 | 1.53E-21 |  |
| Fisuc_2808 | beta-hydroxyacyl-(acyl-carrier-protein) dehydratase FabA/FabZ | 284 | 29 | 591 | 28 | 2.75E-17 |  |
| Fisuc_2809 | phospholipid/glycerol acyltransferase | 220 | 37 | 503 | 33 | 1.42E-27 |  |
| Fisuc_2810 | patatin | 217 | 10 | 492 | 35 | 1.77E-25 |  |
| Fisuc_2812 | diguanylate cyclase/phosphodiesterase | 280 | 45 | 28 | 3 | 0 |  |
| Fisuc_2813* | TatD family hydrolase | 35 | 6 | 24 | 3 | 2.71E-12 |  |
| Fisuc_2814* | GreA/GreB family elongation factor | 17 | 1 | 27 | 3 | 8.44E-05 |  |
| Fisuc_2815 | hypothetical protein | 38 | 5 | 53 | 2 | 2.07E-04 |  |
| Fisuc_2819 | peptidyl-prolyl isomerase | 945 | 106 | 727 | 57 | 1.29E-03 |  |
| Fisuc_2820 | histone protein | 575 | 105 | 490 | 10 | 1.12E-03 |  |
| Fisuc_2822* | LexA family transcriptional repressor | 290 | 23 | 217 | 6 | 1.23E-06 |  |
| Fisuc_2823 | hypothetical protein | 21 | 3 | 20 | 2 | 1.56E-03 |  |
| Fisuc_2824 | spore coat protein CotH | 17 | 3 | 3 | 0 | 0 |  |
| Fisuc_2825 | methyltransferase type 12 | 50 | 4 | 40 | 3 | 3.63E-04 |  |
| Fisuc_2827 | hypothetical protein | 0 | 1 | 1 | 1 | 1.77E-12 |  |
| Fisuc_2828 | hypothetical protein | 1 | 1 | 2 | 1 | 1.20E-30 |  |
| Fisuc_2829 | transglutaminase domain protein | 0 | 1 | 3 | 1 | 2.28E-81 |  |
| Fisuc_2830 | hypothetical protein | 1 | 0 | 3 | 1 | 2.39E-37 |  |
| Fisuc_2831 | acetolactate synthase | 11 | 1 | 18 | 7 | 1.17E-04 |  |
| Fisuc_2832 | hypothetical protein | 10 | 2 | 23 | 5 | 4.11E-21 |  |
| Fisuc_2834 | hypothetical protein | 105 | 15 | 70 | 6 | 7.52E-07 |  |
| Fisuc_2835 | hypothetical protein | 500 | 32 | 151 | 20 | 9.94E-81 |  |
| Fisuc_2836 | hypothetical protein | 241 | 21 | 68 | 10 | 6.07E-120 |  |
| Fisuc_2837* | V-type ATP synthase subunit A | 219 | 61 | 75 | 11 | 2.77E-79 |  |
| Fisuc_2838* | V-type ATP synthase subunit B | 193 | 75 | 66 | 6 | 4.71E-60 |  |
| Fisuc_2839* | V-type ATP synthase subunit D | 179 | 48 | 56 | 7 | 1.73E-91 |  |
| Fisuc_2840* | V-type ATPase 116 kDa subunit | 132 | 51 | 47 | 5 | 8.51E-48 |  |
| Fisuc_2841 | V-type ATP synthase subunit K | 94 | 50 | 41 | 7 | 2.72E-51 |  |
| Fisuc_2842 | V-type ATP synthase subunit K | 131 | 49 | 58 | 6 | 1.06E-45 |  |
| Fisuc_2843 | aminoglycoside phosphotransferase | 493 | 16 | 180 | 16 | 3.48E-53 |  |
| Fisuc_2844 | PTS transporter subunit IIA-like nitrogen-regulatory protein PtsN | 370 | 41 | 147 | 14 | 6.34E-46 |  |
| Fisuc_2845 | hypothetical protein | 104 | 10 | 83 | 3 | 2.00E-03 |  |
| Fisuc_2849 | lipoprotein | 35 | 4 | 26 | 1 | 4.28E-08 |  |
| Fisuc_2850 | hypothetical protein | 52 | 5 | 34 | 1 | 1.83E-14 |  |
| Fisuc_2851 | lipoprotein | 49 | 5 | 31 | 2 | 1.48E-10 |  |
| Fisuc_2852* | nitroreductase | 160 | 49 | 124 | 10 | 2.00E-04 |  |
| Fisuc_2853 | 5'-nucleotidase | 48 | 15 | 30 | 3 | 3.21E-10 |  |
| Fisuc_2855 | hypothetical protein | 68 | 7 | 34 | 3 | 8.57E-38 |  |
| Fisuc_2856 | aminoglycoside phosphotransferase | 74 | 7 | 9 | 1 | 0 |  |
| Fisuc_2860* | TatD-related deoxyribonuclease | 261 | 11 | 131 | 12 | 9.83E-18 |  |
| Fisuc_2861 | hypothetical protein | 338 | 38 | 93 | 6 | 1.49E-196 |  |
| Fisuc_2862 | hypothetical protein | 120 | 3 | 61 | 1 | 2.18E-16 |  |
| Fisuc_2863 | hypothetical protein | 844 | 27 | 529 | 4 | 9.32E-09 |  |
| Fisuc_2864 | hypothetical protein | 97 | 16 | 78 | 6 | 2.32E-03 |  |
| Fisuc_2865 | hypothetical protein | 104 | 17 | 68 | 2 | 3.39E-12 |  |
| Fisuc_2869* | Crp family transcriptional regulator | 55 | 18 | 76 | 9 | 2.13E-03 |  |
| Fisuc_2870* | hypothetical protein | 111 | 18 | 162 | 6 | 1.55E-04 |  |
| Fisuc_2871 | hypothetical protein | 312 | 32 | 500 | 25 | 7.03E-07 |  |
| Fisuc_2873 | hypothetical protein | 62 | 10 | 83 | 4 | 1.13E-03 |  |
| Fisuc_2874 | nitrogen-fixing NifU domain-containing protein | 251 | 4 | 477 | 19 | 4.05E-14 |  |
| Fisuc_2876 | cellulase | 38 | 6 | 9 | 1 | 1.36E-199 | YES |
| Fisuc_2879 | type II site-specific deoxyribonuclease | 265 | 28 | 213 | 15 | 7.00E-03 |  |
| Fisuc_2880 | hypothetical protein | 168 | 20 | 240 | 43 | 5.38E-04 |  |
| Fisuc_2882 | N-formylglutamate amidohydrolase | 44 | 2 | 84 | 9 | 2.98E-13 |  |
| Fisuc_2883 | hypothetical protein | 35 | 2 | 85 | 6 | 4.14E-30 |  |
| Fisuc_2884 | hypothetical protein | 47 | 10 | 41 | 2 | 5.60E-04 |  |
| Fisuc_2885 | hypothetical protein | 49 | 14 | 33 | 3 | 5.14E-13 |  |
| Fisuc_2886 | hypothetical protein | 72 | 20 | 20 | 1 | 1.25E-114 | YES |
| Fisuc_2887* | response regulator receiver modulated metal dependent phosphohydrolase | 291 | 13 | 136 | 12 | 2.53E-33 |  |
| Fisuc_2888 | multi-sensor hybrid histidine kinase | 94 | 11 | 53 | 3 | 1.03E-15 |  |
| Fisuc_2889 | hypothetical protein | 112 | 5 | 95 | 3 | 6.72E-05 |  |
| Fisuc_2890 | hypothetical protein | 69 | 4 | 43 | 4 | 9.21E-15 |  |
| Fisuc_2891 | fructose-bisphosphate aldolase | 2736 | 131 | 5163 | 68 | 7.33E-03 |  |
| Fisuc_2892 | OmpA/MotB domain-containing protein | 297 | 29 | 178 | 7 | 9.80E-13 |  |
| Fisuc_2893 | hypothetical protein | 247 | 19 | 171 | 10 | 2.98E-07 |  |
| Fisuc_2894 | hypothetical protein | 133 | 19 | 43 | 4 | 9.39E-76 |  |
| Fisuc_2895 | hypothetical protein | 2058 | 268 | 6237 | 1396 | 1.07E-23 |  |
| Fisuc_2896 | oligoendopeptidase | 578 | 27 | 399 | 14 | 6.22E-09 |  |
| Fisuc_2897 | hypothetical protein | 1055 | 36 | 484 | 31 | 1.58E-20 |  |
| Fisuc_2898 | integral membrane sensor hybrid histidine kinase | 160 | 20 | 43 | 9 | 9.99E-110 |  |
| Fisuc_2899* | transcriptional regulator, PadR-like family | 278 | 26 | 114 | 26 | 6.71E-39 |  |
| Fisuc_2901 | hypothetical protein | 54 | 10 | 26 | 2 | 2.88E-41 |  |
| Fisuc_2902 | hypothetical protein | 188 | 8 | 95 | 7 | 1.39E-22 |  |
| Fisuc_2903* | prolyl-tRNA synthetase | 178 | 22 | 265 | 2 | 7.65E-03 |  |
| Fisuc_2906 | tRNA uridine 5-carboxymethylaminomethyl modification protein GidA | 43 | 4 | 82 | 7 | 9.29E-12 |  |
| Fisuc_2907 | hypothetical protein | 872 | 95 | 2805 | 339 | 4.43E-52 |  |
| Fisuc_2911 | thiamine biosynthesis protein ThiH | 127 | 10 | 213 | 4 | 1.03E-07 |  |
| Fisuc_2912 | hypothetical protein | 438 | 50 | 39 | 14 | 0 |  |
| Fisuc_2913 | thiamine-phosphate pyrophosphorylase | 22 | 8 | 40 | 4 | 7.04E-10 |  |
| Fisuc_2915 | hypothetical protein | 162 | 22 | 102 | 6 | 2.45E-10 |  |
| Fisuc_2919 | O-glycosyl hydrolase-like protein | 82 | 12 | 8 | 1 | 0 | YES |
| Fisuc_2920 | NADPH-dependent FMN reductase | 29 | 8 | 11 | 2 | 1.58E-56 |  |
| Fisuc_2921 | chaperone protein DnaJ | 159 | 10 | 521 | 30 | 2.14E-68 |  |
| Fisuc_2922 | chaperone protein DnaK | 384 | 9 | 1273 | 218 | 7.91E-29 |  |
| Fisuc_2923 | hypothetical protein | 48 | 6 | 87 | 21 | 7.03E-11 |  |
| Fisuc_2925 | RelE/StbE family addiction module toxin | 121 | 17 | 95 | 14 | 2.72E-06 |  |
| Fisuc_2926 | hypothetical protein | 58 | 8 | 54 | 8 | 7.95E-04 |  |
| Fisuc_2927* | RelB/DinJ family addiction module antitoxin | 102 | 12 | 66 | 3 | 2.91E-14 |  |
| Fisuc_2928 | nucleic acid binding protein | 115 | 12 | 70 | 4 | 4.45E-17 |  |
| Fisuc_2929 | glycoside hydrolase family protein | 89 | 34 | 17 | 3 | 1.26E-282 | YES |
| Fisuc_2930 | GrpE protein HSP-70 cofactor | 139 | 15 | 393 | 17 | 2.43E-42 |  |
| Fisuc_2931 | hypothetical protein | 203 | 41 | 73 | 6 | 9.11E-56 |  |
| Fisuc_2932 | formate/nitrite transporter | 171 | 31 | 125 | 8 | 2.62E-05 |  |
| Fisuc_2935 | radical SAM protein | 24 | 3 | 37 | 3 | 7.69E-06 |  |
| Fisuc_2936 | hypothetical protein | 18 | 4 | 65 | 10 | 7.54E-80 |  |
| Fisuc_2937 | carbamoyl-phosphate synthase small subunit | 42 | 3 | 125 | 27 | 3.98E-64 |  |
| Fisuc_2938 | carbamoyl-phosphate synthase large subunit | 56 | 7 | 130 | 16 | 3.77E-26 |  |
| Fisuc_2940 | hypothetical protein | 39 | 8 | 23 | 2 | 3.34E-18 |  |
| Fisuc_2941 | hypothetical protein | 2 | 1 | 1 | 0 | 1.08E-21 |  |
| Fisuc_2943 | glucuronoarabinoxylan endo-1,4-beta-xylanase | 117 | 33 | 24 | 4 | 3.82E-241 | YES |
| Fisuc_2944* | SNF2-related protein | 27 | 2 | 51 | 13 | 8.61E-11 |  |
| Fisuc_2946 | alkyl sulfatase-like protein | 5 | 1 | 3 | 1 | 1.77E-22 |  |
| Fisuc_2948 | hypothetical protein | 83 | 10 | 21 | 2 | 1.42E-229 |  |
| Fisuc_2949* | phosphoenolpyruvate carboxykinase | 549 | 119 | 1344 | 148 | 4.12E-07 |  |
| Fisuc_2950 | hypothetical protein | 58 | 2 | 47 | 5 | 3.48E-03 |  |
| Fisuc_2951 | radical SAM protein | 62 | 8 | 84 | 3 | 1.73E-03 |  |
| Fisuc_2952 | 6-pyruvoyl-tetrahydropterin synthase | 49 | 14 | 82 | 2 | 3.11E-06 |  |
| Fisuc_2953 | exsB protein | 64 | 11 | 117 | 13 | 2.36E-14 |  |
| Fisuc_2954 | dethiobiotin synthase | 27 | 4 | 53 | 6 | 1.80E-14 |  |
| Fisuc_2955 | 8-amino-7-oxononanoate synthase | 44 | 7 | 79 | 7 | 1.91E-15 |  |
| Fisuc_2956 | 6-carboxyhexanoate--CoA ligase | 54 | 3 | 93 | 6 | 3.15E-11 |  |
| Fisuc_2957 | diguanylate cyclase | 162 | 17 | 79 | 2 | 3.80E-19 |  |
| Fisuc_2958 | adenosylmethionine-8-amino-7-oxononanoate aminotransferase | 75 | 4 | 130 | 2 | 2.95E-09 |  |
| Fisuc_2959 | NADPH-dependent FMN reductase | 118 | 26 | 165 | 12 | 6.11E-04 |  |
| Fisuc_2960 | hypothetical protein | 109 | 22 | 159 | 4 | 1.28E-05 |  |
| Fisuc_2961 | glycoside hydrolase family protein | 12 | 1 | 2 | 1 | 1.99E-228 | YES |
| Fisuc_2962* | single-strand binding protein | 654 | 4 | 1062 | 22 | 1.73E-03 |  |
| Fisuc_2964 | aspartate-semialdehyde dehydrogenase | 1063 | 79 | 2007 | 68 | 3.31E-03 |  |
| Fisuc_2967 | TonB family protein | 2 | 1 | 1 | 1 | 4.59E-09 |  |
| Fisuc_2968 | alanine racemase | 211 | 13 | 150 | 16 | 9.42E-06 |  |
| Fisuc_2970 | hypothetical protein | 337 | 35 | 675 | 68 | 1.71E-16 |  |
| Fisuc_2972 | hypothetical protein | 62 | 6 | 52 | 4 | 3.24E-05 |  |
| Fisuc_2977 | ribonuclease H | 97 | 10 | 135 | 7 | 2.26E-04 |  |
| Fisuc_2982 | hypothetical protein | 563 | 35 | 474 | 53 | 7.29E-05 |  |
| Fisuc_2985 | binding-protein-dependent transport system inner membrane protein | 337 | 10 | 151 | 3 | 2.57E-34 |  |
| Fisuc_2986 | dihydrodipicolinate reductase | 896 | 23 | 453 | 26 | 3.64E-17 |  |
| Fisuc_2987 | ankyrin | 1323 | 117 | 365 | 5 | 6.91E-125 |  |
| Fisuc_2988 | Lytic transglycosylase catalytic | 420 | 26 | 213 | 11 | 1.37E-30 | YES |
| Fisuc_2989 | hypothetical protein | 1223 | 63 | 714 | 58 | 8.04E-15 |  |
| Fisuc_2990 | Thrombospondin type 3 repeat protein | 144 | 15 | 104 | 4 | 6.29E-07 |  |
| Fisuc_2991 | hypothetical protein | 150 | 13 | 130 | 6 | 3.08E-04 |  |
| Fisuc_2992 | glycoside hydrolase family protein | 178 | 16 | 18 | 1 | 0 | YES |
| Fisuc_2993 | hypothetical protein | 13 | 4 | 6 | 1 | 7.37E-30 |  |
| Fisuc_2994 | hypothetical protein | 25 | 3 | 11 | 1 | 3.29E-47 |  |
| Fisuc_2995 | hypothetical protein | 26 | 5 | 12 | 1 | 7.76E-40 |  |
| Fisuc_2996 | hypothetical protein | 29 | 10 | 11 | 1 | 1.48E-57 |  |
| Fisuc_2997 | hypothetical protein | 182 | 8 | 154 | 10 | 1.33E-03 |  |
| Fisuc_2998 | adenine phosphoribosyltransferase | 169 | 9 | 108 | 9 | 3.15E-11 |  |
| Fisuc_3006 | Histidine ammonia-lyase | 44 | 5 | 69 | 2 | 5.30E-06 |  |
| Fisuc_3010 | phosphopantetheine-binding protein | 140 | 7 | 212 | 25 | 7.63E-06 |  |
| Fisuc_3011 | acyl carrier protein | 194 | 7 | 325 | 32 | 5.16E-11 |  |
| Fisuc_3012 | O-methyltransferase | 35 | 6 | 28 | 1 | 7.29E-05 |  |
| Fisuc_3013 | hypothetical protein | 43 | 2 | 35 | 2 | 3.33E-05 |  |
| Fisuc_3014 | phospholipid/glycerol acyltransferase | 20 | 2 | 18 | 2 | 2.24E-05 |  |
| Fisuc_3015 | hypothetical protein | 180 | 35 | 256 | 20 | 7.27E-04 |  |
| Fisuc_3016 | hypothetical protein | 149 | 26 | 218 | 12 | 1.22E-03 |  |
| Fisuc_3018 | hypothetical protein | 206 | 35 | 131 | 11 | 3.92E-09 |  |
| Fisuc_3020 | OmpA/MotB domain-containing protein | 112 | 20 | 151 | 8 | 3.23E-03 |  |
| Fisuc_3025* | CMP/dCMP deaminase zinc-binding protein | 60 | 6 | 101 | 6 | 7.90E-10 |  |
| Fisuc_3028 | 3-oxoacyl-(acyl-carrier-protein) synthase 2 | 259 | 9 | 201 | 19 | 5.30E-06 |  |
| Fisuc_3029 | hypothetical protein | 365 | 20 | 292 | 9 | 5.74E-03 |  |
| Fisuc_3032 | glutamate-1-semialdehyde-2,1-aminomutase | 50 | 6 | 96 | 5 | 7.96E-14 |  |
| Fisuc_3033 | mechanosensitive ion channel MscS | 68 | 5 | 110 | 6 | 1.69E-09 |  |
| Fisuc_3034 | porphobilinogen synthase | 52 | 7 | 77 | 6 | 3.88E-06 |  |
| Fisuc_3037 | CMP/dCMP deaminase zinc-binding protein | 387 | 25 | 297 | 17 | 7.74E-04 |  |
| Fisuc_3041 | radical SAM protein | 98 | 15 | 137 | 4 | 1.74E-03 |  |
| Fisuc_3042 | cell wall/surface repeat protein | 29 | 7 | 24 | 2 | 3.80E-03 |  |
| Fisuc_3046* | 50S ribosomal protein L27 | 3291 | 216 | 8296 | 654 | 1.63E-12 |  |
| Fisuc_3047* | 50S ribosomal protein L21 | 3522 | 284 | 9659 | 788 | 1.27E-06 |  |
| Fisuc_3048 | hypothetical protein | 1 | 1 | 5 | 2 | 9.82E-107 |  |
| Fisuc_3050 | gamma-glutamyl phosphate reductase | 185 | 28 | 242 | 10 | 1.21E-03 |  |
| Fisuc_3053 | hypothetical protein | 9 | 2 | 14 | 1 | 3.52E-04 |  |
| Fisuc_3055 | hypothetical protein | 42 | 2 | 74 | 10 | 2.05E-07 |  |
| Fisuc_3057 | hypothetical protein | 23 | 3 | 21 | 1 | 1.59E-03 |  |
| Fisuc_3058 | hypothetical protein | 65 | 5 | 52 | 2 | 4.34E-03 |  |
| Fisuc_3059 | hypothetical protein | 79 | 5 | 54 | 1 | 2.71E-05 |  |
| Fisuc_3064 | hypothetical protein | 34 | 4 | 17 | 3 | 2.88E-30 |  |
| Fisuc_3065 | GTP-binding proten HflX | 67 | 5 | 44 | 2 | 1.37E-09 |  |
| Fisuc_3066 | phosphatidylglycerophosphatase A | 44 | 6 | 28 | 1 | 1.07E-17 |  |
| Fisuc_3067 | hypothetical protein | 27 | 5 | 18 | 2 | 7.28E-15 |  |
| Fisuc_3071* | Exonuclease RNase T and DNA polymerase III | 70 | 6 | 111 | 9 | 3.74E-07 |  |
| Fisuc_3074 | hypothetical protein | 253 | 18 | 125 | 4 | 1.14E-23 |  |
| Fisuc_3076 | family 3 extracellular solute-binding protein | 289 | 44 | 156 | 21 | 7.56E-21 |  |
| Fisuc_3078 | hypothetical protein | 112 | 28 | 165 | 5 | 7.25E-04 |  |
| Fisuc_3079 | hypothetical protein | 251 | 14 | 94 | 5 | 6.11E-65 |  |
| Fisuc_3080 | hypothetical protein | 102 | 9 | 39 | 4 | 1.58E-51 |  |
| Fisuc_3081 | cellulase | 113 | 59 | 14 | 3 | 0 | YES |
| Fisuc_3083 | hypothetical protein | 14 | 3 | 22 | 1 | 1.93E-04 |  |
| Fisuc_3085 | hypothetical protein | 24 | 1 | 21 | 1 | 2.59E-03 |  |
| Fisuc_3087 | hypothetical protein | 68 | 3 | 142 | 13 | 2.00E-19 |  |
| Fisuc_3088 | hypothetical protein | 60 | 3 | 105 | 12 | 3.51E-11 |  |
| Fisuc_3089 | DNA-binding protein | 33 | 3 | 112 | 26 | 1.52E-79 |  |
| Fisuc_3090 | phosphoribosylformylglycinamidine synthase | 219 | 28 | 344 | 11 | 1.89E-03 |  |
| Fisuc_3094* | nucleotidyl transferase | 130 | 13 | 204 | 12 | 4.08E-06 |  |
| Fisuc_3095 | beta-hydroxyacyl-(acyl-carrier-protein) dehydratase FabA/FabZ | 178 | 32 | 259 | 7 | 4.15E-04 |  |
| Fisuc_3097 | UDP-glucose 4-epimerase | 31 | 13 | 45 | 8 | 2.05E-04 |  |
| Fisuc_3100* | Aldehyde Dehydrogenase | 195 | 26 | 98 | 12 | 1.55E-29 |  |
| Fisuc_3101* | iron-containing alcohol dehydrogenase | 154 | 7 | 92 | 2 | 1.64E-10 |  |
| Fisuc_3102 | thioesterase | 198 | 11 | 142 | 8 | 9.63E-07 |  |
| Fisuc_3103 | 1,4-alpha-glucan-branching protein | 308 | 13 | 259 | 9 | 8.72E-07 | YES |
| Fisuc_3106 | hypothetical protein | 25 | 5 | 23 | 1 | 5.13E-03 |  |
| Fisuc_3111 | carbohydrate-binding protein | 1088 | 172 | 818 | 21 | 7.20E-03 | YES |
| Fisuc_3112 | DSBA oxidoreductase | 923 | 21 | 558 | 7 | 1.29E-11 |  |
| Fisuc_3113 | homoserine O-acetyltransferase | 201 | 4 | 344 | 34 | 2.76E-05 |  |
| Fisuc_3114 | YicC domain-containing protein | 171 | 9 | 251 | 13 | 1.79E-04 |  |
| Fisuc_3116 | Mur ligase middle domain-containing protein | 81 | 11 | 132 | 5 | 1.61E-07 |  |
| Fisuc_3117* | RluA family pseudouridine synthase | 90 | 12 | 138 | 6 | 1.36E-05 |  |
| Fisuc_3118 | lipoprotein signal peptidase | 63 | 12 | 85 | 5 | 1.41E-03 |  |
| Fisuc_3119 | squalene/phytoene synthase | 69 | 18 | 109 | 7 | 7.26E-06 |  |

* Genes have shown differential expression in organisms other than *F. succinogenes* in response to growth rate differences.

Brauer MJ, Huttenhower C, Airoldi EM, Rosenstein R, Matese JC, Gresham D, Boer VM, Troyanskaya OG, Botstein D. Coordination of Growth Rate, Cell Cycle, Stress Response, and Metabolic Activity in Yeast. Fox T, ed. Molecular Biology of the Cell. 2008;19(1):352-367.

You C, Okano H, Hui S, Zhang Z, Kim M, Gunderson CW, Wang Y, Lenz P, Yan D, Hwa T . Coordination of bacterial proteome with metabolism by cyclic AMP signalling. Nature. 2013; 500(7462):301-306.
