# Supplementary material for: Evaluating Models of Cellulose Degradation by Fibrobacter succinogenes S85
Source: PLoS One. 2015 Dec 2;10(12):e0143809. doi: 10.1371/journal.pone.0143809 (PMC4668043; doi:10.1371/journal.pone.0143809)
Supplement: S2 Table — (DOCX) [file pone.0143809.s002.docx]

**Table S2**. Enrichment of *F. succinogenes* ORFs identified as differentially expressed when grown on cellulose relative to glucose compared to all ORFs in the genome according to COG category.

| **COG Category** | **Code** | **RNA-seq^1^** | **All ORFs^2^** |
| --- | --- | --- | --- |
| **Information storage and processing** | | | |
| Translation, ribosomal structure and biogenesis | J | 8 (1.3%)**#** | 158 (7.6%) |
| RNA processing and modification | A | 0 (0%) | 1 (0.05%) |
| Transcription | K | 44 (7.17%)***** | 113 (5.44%) |
| Replication, recombination and repair | L | 31 (5.05%)**#** | 131 (6.3%) |
| Chromatin structure and dynamics | B | 0 (0%) | 0 (0%) |
| **Cellular processes and signaling** | | | |
| Cell cycle control, cell division, chromosome partitioning | D | 4 (0.65%) | 29 (1.4%) |
| Nuclear structure | Y | 0 (0%) | 0 (0%) |
| Defense mechanisms | V | 9 (1.47%) | 35 (1.68%) |
| Signal transduction mechanisms | T | 66 (10.75%)***** | 104 (5%) |
| Cell wall/membrane/envelope biogenesis | M | 49 (7.98%)**#** | 208 (10.01%) |
| Cell motility | N | 15 (2.44%)***** | 20 (0.96%) |
| Cytoskeleton | Z | 0 (0%) | 0 (0%) |
| Extracellular structures | W | 0 (0%) | 0 (0%) |
| Intracellular trafficking, secretion, and vesicular transport | U | 26 (4.23%)* | 60 (2.89%) |
| Posttranslational modification, protein turnover, chaperones | O | 16 (2.61%) | 75 (3.61%) |
| **Metabolism** | | | |
| Energy production and conversion | C | 20 (3.26%)# | 93 (4.48%) |
| Carbohydrate transport and metabolism | G | 76 (12.38%)* | 148 (7.12%) |
| Amino acid transport and metabolism | E | 36 (5.86%)# | 169 (8.13%) |
| Nucleotide transport and metabolism | F | 10 (1.63%)# | 63 (3.03%) |
| Coenzyme transport and metabolism | H | 14 (2.28%)# | 95 (4.57%) |
| Lipid transport and metabolism | I | 19 (3.09%) | 66 (3.18%) |
| Inorganic ion transport and metabolism | P | 14 (2.28%)# | 71 (3.42%) |
| Secondary metabolites biosynthesis, transport and catabolism | Q | 14 (2.28%) | 38 (1.83%) |
| **Poorly characterized** | | | |
| General function prediction only | R | 89 (14.5%)* | 258 (12.42%) |
| Function unknown | S | 54 (8.79%) | 143 (6.88%) |
| **Total ORFs with COG Annotations** |  | **614** | **2078** |

^1^ Total counts of ORFs differentially expressed on cellulose, relative to glucose, are presented according to COG category. Categories that are over- enriched relative to all ORFs in the genome are denoted as (*) at a *P-*value < 0.05 and under-enriched categories are designated as (#) at a *P-*value < 0.05, as determined using a two-tailed Fisher’s Exact Test. These values include only those ORFs with a COG annotation.

^2^ Total counts of ORFs in the *F. succinogenes* genome according to COG category. These values indicate only those ORFs with a COG annotation.
