# Supplementary material for: Evaluating Models of Cellulose Degradation by Fibrobacter succinogenes S85
Source: PLoS One. 2015 Dec 2;10(12):e0143809. doi: 10.1371/journal.pone.0143809 (PMC4668043; doi:10.1371/journal.pone.0143809)
Supplement: S3 Table — (DOCX) [file pone.0143809.s003.docx]

| Protein ID | Description | Outer Membrane | Periplasm | Extracellular Media | CAZy | P-value |
| --- | --- | --- | --- | --- | --- | --- |
| Fisuc_0002 | diaminopimelate dehydrogenase |  | YES | YES |  | 0.05 |
| Fisuc_0003 | hypothetical protein |  | YES |  |  | Unique |
| Fisuc_0009 | hypothetical protein | YES |  | YES |  | 0.46 |
| Fisuc_0024 | ribose 5-phosphate isomerase |  | YES | YES |  | 0.05 |
| Fisuc_0026 | hypothetical protein | YES |  |  |  | Unique |
| Fisuc_0033 | hypothetical protein | YES |  |  |  | Unique |
| Fisuc_0059 | Nucleotidyl transferase |  | YES |  |  | Unique |
| Fisuc_0060 | chaperonin Cpn10 |  |  | YES |  | Unique |
| Fisuc_0061 | chaperonin GroEL | YES | YES | YES |  | 0.05 |
| Fisuc_0063 | hypothetical protein | YES |  | YES |  | 0.05 |
| Fisuc_0067 | 3-isopropylmalate dehydratase, large subunit |  | YES |  |  | Unique |
| Fisuc_0068 | 3-isopropylmalate dehydratase, small subunit |  | YES | YES |  | 0.04 |
| Fisuc_0102 | glyceraldehyde-3-phosphate dehydrogenase, type I | YES | YES | YES |  | 0.19 |
| Fisuc_0106 | peptidase M20 |  |  | YES |  | Unique |
| Fisuc_0116 | aminotransferase class I and II |  | YES | YES |  | 0.64 |
| Fisuc_0137 | Anthranilate synthase |  | YES | YES |  | 0.51 |
| Fisuc_0163 | SirA family protein |  | YES | YES |  | 0.03 |
| Fisuc_0166 | Mov34/MPN/PAD-1 family protein |  | YES |  |  | Unique |
| Fisuc_0171 | hypothetical protein |  |  | YES |  | Unique |
| Fisuc_0172 | cysteine desulfurase, SufS subfamily |  |  | YES |  | Unique |
| Fisuc_0179 | tryptophan synthase, beta subunit | YES | YES | YES |  | 0.05 |
| Fisuc_0181 | aconitate hydratase |  | YES | YES |  | 1.00 |
| Fisuc_0196 | hypothetical protein | YES |  |  |  | Unique |
| Fisuc_0202 | hypothetical protein | YES |  | YES |  | 1.00 |
| Fisuc_0204 | Deoxyribonuclease I |  | YES |  |  | Unique |
| Fisuc_0205 | ribosomal protein S4 | YES |  |  |  | Unique |
| Fisuc_0209 | hypothetical protein |  | YES |  |  | Unique |
| Fisuc_0220 | hypothetical protein | YES |  |  |  | Unique |
| Fisuc_0221 | ribosomal protein L31 |  | YES |  |  | Unique |
| Fisuc_0228 | 30S ribosomal protein S20 |  | YES |  |  | Unique |
| Fisuc_0240 | FAD-dependent pyridine nucleotide-disulphide oxidoreductase | | YES |  |  | Unique |
| Fisuc_0243 | iron-containing alcohol dehydrogenase |  |  | YES |  | Unique |
| Fisuc_0245 | arginine biosynthesis bifunctional protein ArgJ |  | YES |  |  | Unique |
| Fisuc_0246 | transcription elongation factor GreA |  | YES |  |  | Unique |
| Fisuc_0254 | dihydroorotase |  |  | YES |  | Unique |
| Fisuc_0257 | peptidyl-prolyl cis-trans isomerase cyclophilin type | | YES | YES |  | 0.04 |
| Fisuc_0258 | Peptidase M23 |  | YES |  |  | Unique |
| Fisuc_0283 | hypothetical protein |  |  | YES |  | Unique |
| Fisuc_0288 | outer membrane efflux protein |  |  | YES |  | Unique |
| Fisuc_0289 | efflux transporter, RND family, MFP subunit | YES |  |  |  | Unique |
| Fisuc_0301 | hypothetical protein |  | YES |  |  | Unique |
| Fisuc_0313 | ketol-acid reductoisomerase | YES | YES | YES |  | 0.06 |
| Fisuc_0323 | glycoside hydrolase family 44 domain protein |  |  | YES | YES | Unique |
| Fisuc_0331 | pentapeptide repeat protein | YES | YES | YES |  | 0.03 |
| Fisuc_0337 | thiopurine S-methyltransferase |  | YES |  |  | Unique |
| Fisuc_0339 | N-acetyl-gamma-glutamyl-phosphate reductase |  | YES | YES |  | 0.05 |
| Fisuc_0356 | protein of unknown function DUF28 |  | YES |  |  | Unique |
| Fisuc_0358 | phosphoglucomutase/phosphomannomutase alpha/beta/alpha domain I | | YES | YES |  | 1.00 |
| Fisuc_0360 | dihydrodipicolinate synthase |  | YES | YES |  | 0.05 |
| Fisuc_0362 | Endo-1,4-beta-xylanase |  |  | YES | YES | Unique |
| Fisuc_0372 | dihydroxy-acid dehydratase |  | YES | YES |  | 0.51 |
| Fisuc_0377 | fibro-slime family protein | YES |  | YES |  | 0.05 |
| Fisuc_0381 | Phosphoglycerate kinase |  | YES | YES |  | 0.51 |
| Fisuc_0382 | hypothetical protein |  |  | YES |  | Unique |
| Fisuc_0391 | hydro-lyase, Fe-S type, tartrate/fumarate subfamily, beta subunit | | YES | YES |  | 0.38 |
| Fisuc_0392 | mucin-associated surface protein (MASP) | YES | YES | YES |  | 0.03 |
| Fisuc_0393 | glycoside hydrolase family 9 | YES |  | YES | YES | 0.05 |
| Fisuc_0394 | glycoside hydrolase family 9 | YES |  | YES | YES | 0.05 |
| Fisuc_0400 | Transketolase |  | YES | YES |  | 0.82 |
| Fisuc_0426 | glutaminyl-tRNA synthetase |  |  | YES |  | Unique |
| Fisuc_0455 | hypothetical protein |  |  | YES |  | Unique |
| Fisuc_0463 | hypothetical protein |  | YES |  |  | Unique |
| Fisuc_0466 | orotate phosphoribosyltransferase |  |  | YES |  | Unique |
| Fisuc_0470 | methionine aminopeptidase, type I |  | YES | YES |  | 0.12 |
| Fisuc_0471 | glycoside hydrolase family 8 |  |  | YES | YES | Unique |
| Fisuc_0475 | translation elongation factor G |  | YES | YES |  | 0.05 |
| Fisuc_0481 | hypothetical protein | YES |  |  |  | Unique |
| Fisuc_0483 | hypothetical protein |  |  | YES |  | Unique |
| Fisuc_0494 | cysteine synthase A |  | YES |  |  | Unique |
| Fisuc_0495 | hypothetical protein |  |  | YES |  | Unique |
| Fisuc_0497 | Rubrerythrin |  | YES | YES |  | 0.05 |
| Fisuc_0500 | Saccharopine dehydrogenase |  | YES | YES |  | 0.05 |
| Fisuc_0518 | PpiC-type peptidyl-prolyl cis-trans isomerase |  | YES | YES |  | 0.03 |
| Fisuc_0540 | hypothetical protein |  | YES | YES |  | 0.05 |
| Fisuc_0550 | hypothetical protein |  | YES |  |  | Unique |
| Fisuc_0555 | cell wall/surface repeat protein |  |  | YES |  | Unique |
| Fisuc_0556 | phosphoribosylglycinamide formyltransferase 2 |  | YES |  |  | Unique |
| Fisuc_0557 | hypothetical protein |  |  | YES |  | Unique |
| Fisuc_0562 | cell division protein FtsZ | YES |  |  |  | Unique |
| Fisuc_0578 | hypothetical protein |  |  | YES |  | Unique |
| Fisuc_0579 | hypothetical protein |  |  | YES |  | Unique |
| Fisuc_0580 | GLUG domain protein |  |  | YES |  | Unique |
| Fisuc_0581 | Phosphoserine transaminase | YES | YES | YES |  | 0.06 |
| Fisuc_0591 | hypothetical protein |  |  | YES |  | Unique |
| Fisuc_0600 | hypothetical protein | YES |  | YES |  | 0.05 |
| Fisuc_0620 | ornithine carbamoyltransferase |  | YES | YES |  | 1.00 |
| Fisuc_0633 | hypothetical protein | YES |  | YES |  | 0.12 |
| Fisuc_0649 | branched-chain amino acid aminotransferase |  | YES | YES |  | 0.04 |
| Fisuc_0655 | aminotransferase class I and II |  |  | YES |  | Unique |
| Fisuc_0657 | hypothetical protein |  | YES | YES |  | 0.05 |
| Fisuc_0663 | toluene tolerance family protein |  | YES |  |  | Unique |
| Fisuc_0675 | isocitrate dehydrogenase, NADP-dependent |  | YES | YES |  | 0.10 |
| Fisuc_0679 | Pectinesterase |  |  | YES | YES | Unique |
| Fisuc_0688 | hypothetical protein |  |  | YES |  | Unique |
| Fisuc_0708 | beta-lactamase domain protein |  | YES |  |  | Unique |
| Fisuc_0719 | hypothetical protein | YES |  |  |  | Unique |
| Fisuc_0720 | hypothetical protein | YES |  |  |  | Unique |
| Fisuc_0721 | threonine synthase |  | YES |  |  | Unique |
| Fisuc_0727 | Mannan endo-1,4-beta-mannosidase |  |  | YES | YES | Unique |
| Fisuc_0728 | Mannan endo-1,4-beta-mannosidase |  |  | YES | YES | Unique |
| Fisuc_0729 | Mannan endo-1,4-beta-mannosidase |  |  | YES | YES | Unique |
| Fisuc_0730 | Mannan endo-1,4-beta-mannosidase |  |  | YES | YES | Unique |
| Fisuc_0741 | hypothetical protein | YES | YES | YES |  | 0.03 |
| Fisuc_0742 | hypothetical protein |  |  | YES |  | Unique |
| Fisuc_0749 | ribosomal protein S1 |  |  | YES |  | Unique |
| Fisuc_0752 | hypothetical protein | YES | YES | YES |  | 0.05 |
| Fisuc_0758 | thioredoxin |  |  | YES |  | Unique |
| Fisuc_0767 | hypothetical protein | YES |  | YES |  | 0.05 |
| Fisuc_0768 | phosphoglycerate mutase, 2,3-bisphosphoglycerate-independent | YES | YES | YES |  | 0.03 |
| Fisuc_0775 | polysaccharide export protein | YES |  |  |  | Unique |
| Fisuc_0777 | dTDP-4-dehydrorhamnose 3,5-epimerase |  | YES | YES |  | 0.18 |
| Fisuc_0786 | Cellulase | YES |  | YES | YES | 0.03 |
| Fisuc_0787 | hypothetical protein |  |  | YES |  | Unique |
| Fisuc_0788 | hypothetical protein | YES |  | YES |  | 0.46 |
| Fisuc_0798 | hypothetical protein |  |  | YES |  | Unique |
| Fisuc_0817 | FG-GAP repeat protein |  | YES |  |  | Unique |
| Fisuc_0830 | hypothetical protein |  |  | YES |  | Unique |
| Fisuc_0831 | hypothetical protein |  |  | YES |  | Unique |
| Fisuc_0838 | histone family protein DNA-binding protein | YES | YES |  |  | 0.04 |
| Fisuc_0844 | OmpA/MotB domain protein |  | YES |  |  | Unique |
| Fisuc_0845 | Pyruvate carboxylase | YES |  |  |  | Unique |
| Fisuc_0851 | carboxyl-terminal protease |  | YES |  |  | Unique |
| Fisuc_0860 | 4-alpha-glucanotransferase |  |  | YES | YES | Unique |
| Fisuc_0866 | hypothetical protein | YES |  | YES |  | 0.04 |
| Fisuc_0886 | hypothetical protein |  |  | YES |  | Unique |
| Fisuc_0888 | hypothetical protein | YES |  |  |  | Unique |
| Fisuc_0897 | Cellulase |  |  | YES | YES | Unique |
| Fisuc_0898 | glutamate synthase (NADPH), homotetrameric |  | YES | YES |  | 0.05 |
| Fisuc_0910 | peptidase C14 caspase catalytic subunit p20 |  |  | YES |  | Unique |
| Fisuc_0947 | translation elongation factor Ts |  | YES | YES |  | 0.05 |
| Fisuc_0949 | Electron transfer flavoprotein alpha/beta-subunit | YES | YES |  |  | 0.04 |
| Fisuc_0951 | acyl-CoA dehydrogenase domain protein | YES | YES |  |  | 0.05 |
| Fisuc_0974 | ribosomal protein S9 | YES |  |  |  | Unique |
| Fisuc_0990 | NAD-dependent epimerase/dehydratase |  | YES |  |  | Unique |
| Fisuc_1000 | Lytic transglycosylase catalytic |  | YES |  | YES | Unique |
| Fisuc_1004 | DNA-directed RNA polymerase, beta' subunit | YES |  |  |  | Unique |
| Fisuc_1013 | hypothetical protein | YES | YES | YES |  | 0.06 |
| Fisuc_1028 | hypothetical protein |  |  | YES |  | Unique |
| Fisuc_1031 | hypothetical protein |  |  | YES |  | Unique |
| Fisuc_1044 | formate acetyltransferase |  | YES | YES |  | 0.05 |
| Fisuc_1071 | O-acetylhomoserine/O-acetylserine sulfhydrylase | | YES | YES |  | 0.05 |
| Fisuc_1072 | cysteine synthase A | YES | YES | YES |  | 0.02 |
| Fisuc_1079 | Cystathionine gamma-synthase |  |  | YES |  | Unique |
| Fisuc_1107 | Glucokinase |  | YES | YES |  | 0.26 |
| Fisuc_1126 | O-acetylhomoserine/O-acetylserine sulfhydrylase | |  | YES |  | Unique |
| Fisuc_1142 | hypothetical protein |  |  | YES |  | Unique |
| Fisuc_1151 | OmpA/MotB domain protein |  |  | YES |  | Unique |
| Fisuc_1176 | hypothetical protein |  |  | YES |  | Unique |
| Fisuc_1202 | metal dependent phosphohydrolase |  | YES |  |  | Unique |
| Fisuc_1204 | Glucose-6-phosphate isomerase |  | YES | YES |  | 1.00 |
| Fisuc_1219 | glycoside hydrolase family 8 |  |  | YES | YES | Unique |
| Fisuc_1222 | hypothetical protein |  |  | YES |  | Unique |
| Fisuc_1223 | hypothetical protein | YES | YES | YES |  | 0.03 |
| Fisuc_1224 | Cellulase | YES |  | YES | YES | 0.05 |
| Fisuc_1230 | extracellular solute-binding protein family 5 | YES |  |  |  | Unique |
| Fisuc_1232 | glycosyl transferase, family 2 | YES |  | YES |  | 0.03 |
| Fisuc_1238 | sigma 54 modulation protein/ribosomal protein S30EA | | YES | YES |  | 0.05 |
| Fisuc_1244 | Indolepyruvate ferredoxin oxidoreductase |  | YES | YES |  | 0.08 |
| Fisuc_1247 | hypothetical protein |  |  | YES |  | Unique |
| Fisuc_1261 | enolase | YES | YES | YES |  | 0.06 |
| Fisuc_1268 | ribosomal protein S7 | YES |  |  |  | Unique |
| Fisuc_1270 | DNA-directed RNA polymerase, beta' subunit | YES |  |  |  | Unique |
| Fisuc_1271 | DNA-directed RNA polymerase, beta subunit | YES |  | YES |  | 0.49 |
| Fisuc_1272 | ribosomal protein L7/L12 |  | YES | YES |  | 0.05 |
| Fisuc_1275 | ribosomal protein L11 | YES |  |  |  | Unique |
| Fisuc_1279 | translation elongation factor Tu ; Duplicate proteins: 646369730 | YES | YES | YES |  | 0.05 |
| Fisuc_1284 | hypothetical protein |  |  | YES |  | Unique |
| Fisuc_1286 | adenosylhomocysteinase |  | YES | YES |  | 0.18 |
| Fisuc_1313 | hypothetical protein |  |  | YES |  | Unique |
| Fisuc_1314 | hypothetical protein |  |  | YES |  | Unique |
| Fisuc_1316 | PEGA domain protein | YES | YES | YES |  | 0.06 |
| Fisuc_1317 | hypothetical protein |  |  | YES |  | Unique |
| Fisuc_1319 | hypothetical protein | YES |  | YES |  | 0.05 |
| Fisuc_1320 | two component transcriptional regulator, winged helix family | |  | YES |  | Unique |
| Fisuc_1325 | phosphate acetyltransferase |  | YES | YES |  | 0.50 |
| Fisuc_1326 | fibro-slime family protein |  |  | YES |  | Unique |
| Fisuc_1327 | fibro-slime family protein |  |  | YES |  | Unique |
| Fisuc_1334 | DNA polymerase III, beta subunit |  |  | YES |  | Unique |
| Fisuc_1351 | 5- methyltetrahydropteroyltriglutamate/homocysteine S-methyltransferase | YES | YES | YES |  | 0.04 |
| Fisuc_1385 | hypothetical protein |  |  | YES |  | Unique |
| Fisuc_1390 | trigger factor |  | YES | YES |  | 0.13 |
| Fisuc_1396 | ribosomal protein L17 | YES |  | YES |  | 0.07 |
| Fisuc_1397 | DNA-directed RNA polymerase, alpha subunit |  |  | YES |  | Unique |
| Fisuc_1398 | 30S ribosomal protein S11 | YES | YES |  |  | 0.07 |
| Fisuc_1403 | ribosomal protein L15 | YES |  |  |  | Unique |
| Fisuc_1405 | ribosomal protein S5 | YES |  |  |  | Unique |
| Fisuc_1412 | ribosomal protein L14 | YES | YES |  |  | 0.05 |
| Fisuc_1417 | ribosomal protein L22 | YES | YES |  |  | 0.04 |
| Fisuc_1419 | ribosomal protein L2 | YES |  |  |  | Unique |
| Fisuc_1421 | ribosomal protein L4/L1e | YES |  |  |  | Unique |
| Fisuc_1423 | ribosomal protein S10 | YES | YES |  |  | 0.50 |
| Fisuc_1426 | Cellulase |  |  | YES | YES | Unique |
| Fisuc_1460 | histidine acid phosphatase |  | YES |  |  | Unique |
| Fisuc_1465 | Extracellular ligand-binding receptor | YES |  |  |  | Unique |
| Fisuc_1472 | phosphoribosylformylglycinamidine cyclo-ligase |  | YES |  |  | Unique |
| Fisuc_1473 | Cellulase | YES |  | YES | YES | 0.32 |
| Fisuc_1474 | fibro-slime family protein | YES |  | YES |  | 0.05 |
| Fisuc_1485 | hypothetical protein |  |  | YES |  | Unique |
| Fisuc_1486 | Rubrerythrin | YES |  | YES |  | 0.07 |
| Fisuc_1490 | hypothetical protein |  |  | YES |  | Unique |
| Fisuc_1494 | hypothetical protein |  |  | YES |  | Unique |
| Fisuc_1510 | hypothetical protein |  |  | YES |  | Unique |
| Fisuc_1515 | glycogen synthase |  | YES | YES | YES | 0.82 |
| Fisuc_1519 | hypothetical protein | YES |  |  |  | Unique |
| Fisuc_1523 | glycoside hydrolase family 5 | YES |  | YES | YES | 0.13 |
| Fisuc_1525 | hypothetical protein |  |  | YES | YES | Unique |
| Fisuc_1526 | hypothetical protein | YES |  | YES |  | 0.38 |
| Fisuc_1527 | hypothetical protein | YES |  | YES |  | 0.38 |
| Fisuc_1528 | hypothetical protein | YES |  | YES |  | 0.28 |
| Fisuc_1529 | hypothetical protein | YES |  |  |  | Unique |
| Fisuc_1530 | glycoside hydrolase family 18 | YES |  | YES | YES | 0.04 |
| Fisuc_1531 | Cellulase |  | YES |  | YES | Unique |
| Fisuc_1592 | OmpA/MotB domain protein | YES | YES | YES |  | 0.03 |
| Fisuc_1595 | Phosphoglycerate mutase |  |  | YES |  | Unique |
| Fisuc_1596 | Phosphoglycerate mutase |  |  | YES |  | Unique |
| Fisuc_1597 | hypothetical protein | YES |  |  |  | Unique |
| Fisuc_1627 | adenylosuccinate synthetase |  | YES | YES |  | 1.00 |
| Fisuc_1632 | FG-GAP repeat protein | YES |  | YES |  | 0.32 |
| Fisuc_1641 | hypothetical protein |  | YES |  | YES | Unique |
| Fisuc_1649 | orotidine 5'-phosphate decarboxylase |  | YES |  |  | Unique |
| Fisuc_1651 | hypothetical protein |  | YES |  |  | Unique |
| Fisuc_1660 | hypothetical protein | YES |  | YES |  | 0.05 |
| Fisuc_1747 | ribosomal protein L34 |  | YES |  |  | Unique |
| Fisuc_1754 | two component regulator propeller domain protein | YES |  |  |  | Unique |
| Fisuc_1757 | hypothetical protein |  |  | YES |  | Unique |
| Fisuc_1761 | hypothetical protein |  |  | YES |  | Unique |
| Fisuc_1762 | glycoside hydrolase family 43 |  | YES | YES | YES | 0.18 |
| Fisuc_1763 | Carbohydrate binding family 6 |  |  | YES | YES | Unique |
| Fisuc_1764 | Carbohydrate binding family 6 |  |  | YES | YES | Unique |
| Fisuc_1765 | Glucuronoarabinoxylan endo-1,4-beta-xylanase |  |  | YES | YES | Unique |
| Fisuc_1767 | Carbohydrate binding family 6 |  |  | YES | YES | Unique |
| Fisuc_1773 | Alpha-galactosidase |  |  | YES | YES | Unique |
| Fisuc_1788 | glycoside hydrolase family 2 TIM barrel |  |  | YES | YES | Unique |
| Fisuc_1790 | Carbohydrate binding family 6 |  |  | YES | YES | Unique |
| Fisuc_1791 | Carbohydrate binding family 6 | YES | YES | YES | YES | 0.51 |
| Fisuc_1793 | Carbohydrate binding family 6 |  |  | YES | YES | Unique |
| Fisuc_1802 | glycoside hydrolase family 8 | YES | YES | YES | YES | 0.02 |
| Fisuc_1850 | ribosome recycling factor |  | YES | YES |  | 0.20 |
| Fisuc_1859 | glycoside hydrolase family 9 | YES |  | YES | YES | 0.05 |
| Fisuc_1860 | glycoside hydrolase family 9 |  |  | YES | YES | Unique |
| Fisuc_1875 | hypothetical protein | YES |  | YES |  | 0.08 |
| Fisuc_1877 | hypothetical protein | YES |  |  |  | Unique |
| Fisuc_1891 | OmpA/MotB domain protein | YES | YES | YES |  | 0.05 |
| Fisuc_1892 | TPR repeat-containing protein | YES | YES | YES |  | 0.03 |
| Fisuc_1893 | TPR repeat-containing protein | YES | YES | YES |  | 0.04 |
| Fisuc_1894 | MotA/TolQ/ExbB proton channel | YES |  |  |  | Unique |
| Fisuc_1897 | TonB family protein |  | YES |  |  | Unique |
| Fisuc_1898 | hypothetical protein | YES |  | YES |  | 0.08 |
| Fisuc_1907 | Spore coat protein CotH | YES |  | YES |  | 0.05 |
| Fisuc_1931 | Carbohydrate-binding CenC domain protein |  |  | YES | YES | Unique |
| Fisuc_1965 | hypothetical protein |  |  | YES |  | Unique |
| Fisuc_1974 | polysaccharide deacetylase |  |  | YES |  | Unique |
| Fisuc_1979 | fibro-slime family protein | YES |  | YES |  | 0.05 |
| Fisuc_1991 | Pectate lyase-like protein |  |  | YES | YES | Unique |
| Fisuc_2011 | Cellulase |  |  | YES | YES | Unique |
| Fisuc_2012 | hypothetical protein |  |  | YES | YES | Unique |
| Fisuc_2015 | fatty acid/phospholipid synthesis protein PlsX |  | YES | YES |  | 0.04 |
| Fisuc_2016 | 3-oxoacyl-(acyl-carrier-protein) reductase | YES | YES |  |  | 0.05 |
| Fisuc_2017 | acyl carrier protein |  |  | YES |  | Unique |
| Fisuc_2022 | hypothetical protein |  |  | YES |  | Unique |
| Fisuc_2026 | hypothetical protein |  | YES |  |  | Unique |
| Fisuc_2030 | aldo/keto reductase |  | YES |  |  | Unique |
| Fisuc_2031 | fibro-slime family protein | YES | YES | YES |  | 0.03 |
| Fisuc_2032 | pyruvate carboxyltransferase |  | YES | YES |  | 0.05 |
| Fisuc_2033 | glycoside hydrolase family 9 |  |  | YES | YES | Unique |
| Fisuc_2041 | hypothetical protein | YES | YES | YES |  | 0.03 |
| Fisuc_2065 | glycoside hydrolase family 3 domain protein |  | YES |  | YES | Unique |
| Fisuc_2068 | hypothetical protein | YES |  | YES |  | 0.07 |
| Fisuc_2069 | hypothetical protein | YES |  | YES |  | 0.08 |
| Fisuc_2071 | hypothetical protein |  |  | YES |  | Unique |
| Fisuc_2111 | Phosphoglycerate mutase | YES |  | YES |  | 0.64 |
| Fisuc_2120 | outer membrane protein assembly complex, YaeT protein | YES |  | YES |  | 0.10 |
| Fisuc_2122 | geranylgeranyl reductase | YES |  |  |  | Unique |
| Fisuc_2124 | hypothetical protein |  |  | YES |  | Unique |
| Fisuc_2142 | GLUG domain protein |  |  | YES |  | Unique |
| Fisuc_2144 | hypothetical protein |  |  | YES |  | Unique |
| Fisuc_2145 | GLUG domain protein |  |  | YES |  | Unique |
| Fisuc_2203 | hypothetical protein |  | YES |  |  | Unique |
| Fisuc_2249 | hypothetical protein | YES | YES | YES |  | 0.04 |
| Fisuc_2250 | O-Glycosyl hydrolase-like protein | YES |  | YES | YES | 0.05 |
| Fisuc_2253 | Homoserine dehydrogenase |  |  | YES |  | Unique |
| Fisuc_2255 | nicotinate-nucleotide pyrophosphorylase |  | YES | YES |  | 0.25 |
| Fisuc_2261 | hypothetical protein | YES |  |  |  | Unique |
| Fisuc_2262 | hypothetical protein | YES |  | YES |  | 0.04 |
| Fisuc_2272 | hypothetical protein | YES |  | YES |  | 0.05 |
| Fisuc_2276 | peptidase U62 modulator of DNA gyrase |  | YES |  |  | Unique |
| Fisuc_2284 | hypothetical protein |  |  | YES |  | Unique |
| Fisuc_2285 | hypothetical protein | YES |  |  |  | Unique |
| Fisuc_2293 | fibro-slime family protein |  |  | YES |  | Unique |
| Fisuc_2296 | translation initiation factor IF-2 | YES |  |  |  | Unique |
| Fisuc_2308 | hypothetical protein | YES | YES | YES |  | 0.30 |
| Fisuc_2314 | hypothetical protein | YES |  | YES |  | 0.82 |
| Fisuc_2317 | endo-1,4-beta-glucanase/xyloglucanase, putative, gly74A | |  | YES | YES | Unique |
| Fisuc_2318 | hypothetical protein |  |  | YES |  | Unique |
| Fisuc_2326 | hypothetical protein | YES |  |  |  | Unique |
| Fisuc_2358 | pyruvate carboxyltransferase |  | YES |  |  | Unique |
| Fisuc_2362 | glycoside hydrolase family 9 | YES |  | YES | YES | 0.04 |
| Fisuc_2363 | Pectate lyase/Amb allergen |  |  | YES | YES | Unique |
| Fisuc_2364 | Cellulase | YES | YES | YES | YES | 0.03 |
| Fisuc_2367 | preprotein translocase, SecG subunit |  | YES | YES |  | 0.10 |
| Fisuc_2368 | triosephosphate isomerase | YES | YES | YES |  | 0.03 |
| Fisuc_2370 | hypothetical protein | YES |  | YES |  | 0.64 |
| Fisuc_2377 | Cadherin | YES |  | YES |  | 0.05 |
| Fisuc_2378 | Cadherin | YES | YES | YES |  | 0.06 |
| Fisuc_2380 | hypothetical protein | YES | YES |  |  | 0.49 |
| Fisuc_2384 | putative seryl-tRNA synthetase (serine--tRNA ligase) (SerRS) | YES |  |  |  | Unique |
| Fisuc_2388 | polyribonucleotide nucleotidyltransferase | YES | YES | YES |  | 0.03 |
| Fisuc_2392 | D-isomer specific 2-hydroxyacid dehydrogenase NAD-binding protein | | YES | YES |  | 0.08 |
| Fisuc_2395 | seryl-tRNA synthetase |  | YES |  |  | Unique |
| Fisuc_2403 | ribosomal protein L19 | YES | YES |  |  | 0.04 |
| Fisuc_2424 | glycoside hydrolase family 16 |  |  | YES | YES | Unique |
| Fisuc_2439 | ribosomal 5S rRNA E-loop binding protein Ctc/L25/TL5 | YES | YES |  |  | 1.00 |
| Fisuc_2442 | Endo-1,4-beta-xylanase |  |  | YES | YES | Unique |
| Fisuc_2477 | Carbohydrate binding family 6 |  |  | YES | YES | Unique |
| Fisuc_2478 | Carbohydrate binding family 6 |  |  | YES | YES | Unique |
| Fisuc_2479 | lipolytic protein G-D-S-L family |  |  | YES | YES | Unique |
| Fisuc_2482 | Glycine hydroxymethyltransferase | YES | YES | YES |  | 0.06 |
| Fisuc_2491 | Nucleoside-diphosphate kinase |  |  | YES |  | Unique |
| Fisuc_2492 | succinate dehydrogenase (or fumarate reductase) cytochrome b subunit, b558 family | YES |  |  |  | Unique |
| Fisuc_2494 | 4Fe-4S ferredoxin iron-sulfur binding domain protein | YES |  |  |  | Unique |
| Fisuc_2506 | hypothetical protein | YES |  | YES |  | 0.03 |
| Fisuc_2509 | OmpA/MotB domain protein | YES |  | YES |  | 0.05 |
| Fisuc_2524 | PDZ/DHR/GLGF domain protein |  |  | YES |  | Unique |
| Fisuc_2533 | translation elongation factor P |  | YES |  |  | Unique |
| Fisuc_2550 | hypothetical protein |  | YES |  |  | Unique |
| Fisuc_2555 | hypothetical protein | YES | YES |  |  | 0.05 |
| Fisuc_2556 | UDP-glucose 4-epimerase |  | YES |  |  | Unique |
| Fisuc_2577 | protein of unknown function DUF323 | YES |  |  |  | Unique |
| Fisuc_2579 | glycoside hydrolase family 8 | YES |  | YES | YES | 0.05 |
| Fisuc_2591 | peptidase U62 modulator of DNA gyrase |  | YES |  |  | Unique |
| Fisuc_2612 | hypothetical protein | YES | YES | YES |  | 0.20 |
| Fisuc_2615 | malate dehydrogenase, NAD-dependent | YES | YES | YES |  | 0.05 |
| Fisuc_2624 | Fibronectin type III domain protein | YES |  | YES |  | 0.05 |
| Fisuc_2640 | adenylate kinase |  | YES | YES |  | 0.82 |
| Fisuc_2644 | 3-isopropylmalate dehydrogenase |  | YES | YES |  | 0.27 |
| Fisuc_2648 | DegT/DnrJ/EryC1/StrS aminotransferase |  | YES | YES |  | 0.18 |
| Fisuc_2670 | conserved hypothetical peptidase | YES | YES | YES |  | 0.03 |
| Fisuc_2704 | Glucosylceramidase | YES | YES |  | YES | 0.82 |
| Fisuc_2716 | hypothetical protein |  | YES | YES |  | 0.25 |
| Fisuc_2718 | hypothetical protein |  |  | YES |  | Unique |
| Fisuc_2744 | Tetratricopeptide repeat protein |  |  | YES |  | Unique |
| Fisuc_2746 | hypothetical protein |  |  | YES |  | Unique |
| Fisuc_2752 | hypothetical protein |  |  | YES |  | Unique |
| Fisuc_2754 | peptidylprolyl isomerase FKBP-type |  |  | YES |  | Unique |
| Fisuc_2755 | hypothetical protein | YES |  | YES |  | 0.05 |
| Fisuc_2762 | PpiC-type peptidyl-prolyl cis-trans isomerase | YES |  |  |  | Unique |
| Fisuc_2764 | OmpA/MotB domain protein |  | YES |  |  | Unique |
| Fisuc_2780 | hypothetical protein |  |  | YES |  | Unique |
| Fisuc_2795 | hypothetical protein | YES | YES | YES |  | 0.03 |
| Fisuc_2799 | putative avirulence protein |  |  | YES |  | Unique |
| Fisuc_2803 | carboxyl transferase | YES |  |  |  | Unique |
| Fisuc_2804 | malonyl CoA-acyl carrier protein transacylase |  | YES | YES |  | 1.00 |
| Fisuc_2806 | Beta-ketoacyl synthase | YES | YES | YES |  | 0.06 |
| Fisuc_2811 | Glu/Leu/Phe/Val dehydrogenase |  | YES | YES |  | 0.05 |
| Fisuc_2816 | hypothetical protein |  | YES | YES |  | 0.83 |
| Fisuc_2819 | Peptidylprolyl isomerase |  |  | YES |  | Unique |
| Fisuc_2824 | Spore coat protein CotH |  |  | YES |  | Unique |
| Fisuc_2833 | aspartyl-tRNA synthetase |  |  | YES |  | Unique |
| Fisuc_2848 | methionyl-tRNA synthetase |  | YES |  |  | Unique |
| Fisuc_2852 | nitroreductase |  | YES | YES |  | 0.07 |
| Fisuc_2863 | hypothetical protein | YES | YES | YES |  | 0.06 |
| Fisuc_2866 | glutamyl-tRNA(Gln) amidotransferase, B subunit |  | YES |  |  | Unique |
| Fisuc_2868 | hypothetical protein |  |  | YES |  | Unique |
| Fisuc_2872 | IMP cyclohydrolase |  | YES | YES |  | 0.04 |
| Fisuc_2881 | pyruvate ferredoxin/flavodoxin oxidoreductase | YES | YES | YES |  | 0.06 |
| Fisuc_2891 | fructose-1,6-bisphosphate aldolase, class II | YES | YES | YES |  | 0.03 |
| Fisuc_2892 | OmpA/MotB domain protein | YES |  |  |  | Unique |
| Fisuc_2893 | hypothetical protein | YES |  | YES |  | 0.03 |
| Fisuc_2894 | hypothetical protein |  |  | YES |  | Unique |
| Fisuc_2895 | hypothetical protein | YES |  |  |  | Unique |
| Fisuc_2900 | cellodextrin-phosphorylase | YES | YES |  | YES | 0.05 |
| Fisuc_2915 | hypothetical protein | YES |  | YES |  | 0.25 |
| Fisuc_2917 | OmpA/MotB domain protein | YES | YES | YES |  | 0.03 |
| Fisuc_2919 | O-Glycosyl hydrolase-like protein |  |  | YES | YES | Unique |
| Fisuc_2922 | chaperone protein DnaK | YES | YES | YES |  | 0.06 |
| Fisuc_2945 | hypothetical protein |  |  | YES |  | Unique |
| Fisuc_2949 | Phosphoenolpyruvate carboxykinase (GTP) | YES | YES | YES |  | 0.03 |
| Fisuc_2959 | NADPH-dependent FMN reductase |  | YES | YES |  | 0.03 |
| Fisuc_2964 | aspartate-semialdehyde dehydrogenase | YES | YES | YES |  | 0.02 |
| Fisuc_2966 | peptidase M29, aminopeptidase II |  |  | YES |  | Unique |
| Fisuc_2971 | diphosphate/fructose-6-phosphate 1-phosphotransferase | YES | YES | YES |  | 0.04 |
| Fisuc_2974 | PpiC-type peptidyl-prolyl cis-trans isomerase |  | YES |  |  | Unique |
| Fisuc_2982 | hypothetical protein | YES |  | YES |  | 0.10 |
| Fisuc_2986 | dihydrodipicolinate reductase |  | YES | YES |  | 0.05 |
| Fisuc_2987 | Ankyrin | YES |  | YES |  | 0.18 |
| Fisuc_3015 | hypothetical protein |  |  | YES |  | Unique |
| Fisuc_3018 | hypothetical protein | YES |  | YES |  | 0.05 |
| Fisuc_3028 | 3-oxoacyl-(acyl-carrier-protein) synthase 2 |  | YES |  |  | Unique |
| Fisuc_3045 | pyridoxal-phosphate dependent TrpB-like enzyme | YES | YES | YES |  | 0.05 |
| Fisuc_3047 | ribosomal protein L21 | YES |  |  |  | Unique |
| Fisuc_3049 | Beta-galactosidase |  | YES |  | YES | Unique |
| Fisuc_3050 | gamma-glutamyl phosphate reductase |  |  | YES |  | Unique |
| Fisuc_3070 | 3-oxoacyl-(acyl-carrier-protein) synthase 2 | YES | YES | YES |  | 0.03 |
| Fisuc_3090 | phosphoribosylformylglycinamidine synthase |  | YES | YES |  | 0.05 |
| Fisuc_3095 | Beta-hydroxyacyl-(acyl-carrier-protein) dehydratase FabA/FabZ | | YES | YES |  | 0.12 |
| Fisuc_3103 | 1,4-alpha-glucan branching enzyme |  |  | YES | YES | Unique |
| Fisuc_3111 | Carbohydrate binding family 11 | YES | YES |  | YES | 0.05 |
| Fisuc_3115 | amidohydrolase |  | YES |  |  | Unique |
