# Supplementary material for: Evaluating Models of Cellulose Degradation by Fibrobacter succinogenes S85
Source: PLoS One. 2015 Dec 2;10(12):e0143809. doi: 10.1371/journal.pone.0143809 (PMC4668043; doi:10.1371/journal.pone.0143809)
Supplement: S4 Table — (DOCX) [file pone.0143809.s004.docx]

| Sample | Cellulose 1 | Cellulose 2 | Cellulose 3 | Glucose 1 | Glucose 2 | Glucose 3 |
| --- | --- | --- | --- | --- | --- | --- |
| Total Reads | 25564948 | 9793929 | 10260265 | 8392760 | 24822805 | 52063246 |
| Aligned Reads | 19328608 | 7718950 | 9313014 | 8006254 | 24031555 | 50963337 |
| Percent Aligned | 75.6 | 78.8 | 90.8 | 95.4 | 96.8 | 97.9 |
