# Supplementary material for: Evaluating Models of Cellulose Degradation by Fibrobacter succinogenes S85
Source: PLoS One. 2015 Dec 2;10(12):e0143809. doi: 10.1371/journal.pone.0143809 (PMC4668043; doi:10.1371/journal.pone.0143809)
Supplement: S5 Table — (DOCX) [file pone.0143809.s005.docx]

| Component | Concentration |
| --- | --- |
| Na_2_CO_3_ | 30.2 mM |
| NaCl | 14.1 mM |
| KH_2_PO_4_ | 6.61 mM |
| L-Cysteine-HCl | 6.35 mM |
| (NH_4_)_2_SO_4_ | 6.23 mM |
| Isobutyric acid | 681 μM |
| Isovaleric acid | 587 μM |
| Valeric acid | 587 μM |
| 2-methylbutyric acid | 587 μM |
| CaCl_2_ | 408 μM |
| MgCl_2_ | 381 μM |
| MnCl_2_ | 127 μM |
| FeSO_4_ | 65.7 μM |
| ZnCl_2_ | 63.2 μM |
| CoCl_2_ | 7.98 μM |
| Nicotinamide | 1.64 μM |
| Pyridoxine HCl | 973 nM |
| Thiamine HCl | 593 nM |
| Riboflavin | 531 nM |
| Ca-D-pantothenate | 420 nM |
| p-Aminobenzoic acid | 72.9 nM |
| Biotin | 20.5 nM |
| Folic acid | 2.83 nM |
| Tetrahydrofolic acid | 2.81 nM |
| Vitamin B12 | 1.48 nM |
